# Supplementary material for: Trends in weight gain recorded in English primary care before and during the Coronavirus-19 pandemic: An observational cohort study using the OpenSAFELY platform
Source: PLoS Med. 2024 Jun 24;21(6):e1004398. doi: 10.1371/journal.pmed.1004398 (PMC11249215; doi:10.1371/journal.pmed.1004398)
Supplement: S1 Protocol — (DOCX) [file pmed.1004398.s002.docx]

Has the COVID-19 pandemic impacted the need for weight management interventions amongst adults in the UK? An evaluation of pandemic-associated changes in Body Mass Index and metabolic parameters

Protocol Version 1.2: 25^th^ October 2021

LSHTM LEO project ID: 26536

**Chief Investigator**

Name: Dr Miriam Samuel

NIHR Funded ACF in Primary Care

Address: Centre for Primary Care

Wolfson Institute of Primary Care

Queen Mary University of London

Email: [m.samuel@qmul.ac.uk](mailto:m.samuel@qmul.ac.uk)

**Co-Investigators**

Name: Dr Rohini Mathur

Assistant Professor in Epidemiology and Public Health

Address: Room 248c LSHTM

Keppel Street

London

WC1E 7HT

Email: [rohini.mathur@lshtm.ac.uk](mailto:rohini.mathur@lshtm.ac.uk)

Name: Dr Sarah Finer

Clinical Senior Lecturer in Diabetes

Address: Centre for Primary Care

Wolfson Institute of Primary Care

Queen Mary University of London

Email: s.finer@qmul.ac.uk

Table of Contents

[Glossary of Abbreviations 5](#_Toc86165844)

[Study Summary 5](#_Toc86165845)

[INTRODUCTION 6](#_Toc86165846)

[STUDY OBJECTIVES 7](#_Toc86165847)

[Method 7](#_Toc86165848)

[Data Source: OpenSAFELY 7](#_Toc86165849)

[Study Population 8](#_Toc86165850)

[Study Measures 8](#_Toc86165851)

[Study Duration 9](#_Toc86165852)

[STATISTICs and data analysis 10](#_Toc86165853)

[Risks and Benefits 10](#_Toc86165854)

[Data security 11](#_Toc86165855)

[Confidentiality 11](#_Toc86165856)

[Risk of bias in the results 11](#_Toc86165857)

[regulatory issues including Ethics 11](#_Toc86165858)

[Confidentiality 12](#_Toc86165859)

[Funding 12](#_Toc86165860)

[PROTOCOL DEVELOPMENT 12](#_Toc86165861)

[Publication Policy 12](#_Toc86165862)

[References 13](#_Toc86165863)

# Glossary of Abbreviations

| COVID-19 | Coronavirus-19 |
| --- | --- |
| EHR | Electronic Health Records |
| EMIS | Egerton Medical Information System – A provider of GP EHRs |
| GP | General Practitioner |
| TPP | The Phoenix Partnership – A provider of GP EHRs |

# Study Summary

| **TITLE** | How has the COVID-19 pandemic impacted the need for targeted weight management interventions amongst adults in the UK? An evaluation of pandemic-associated changes in BMI and metabolic parameters. |
| --- | --- |
| **DESIGN** | Secondary analysis of routinely collected electronic health records |
| **AIMS** | To evaluate, amongst adults living in the UK, how trends in measures of healthy weight (such as Body Mass Index) and metabolic risk factors (such as blood pressure (BP), blood lipid profiles, chronic blood glucose levels measured by Haemoglobin A1c (HbA1c) and smoking status) have changed since the onset of the COVID-19 pandemic using data in routinely collected GP electronic health records (EHRs) |
| **OUTCOME MEASURES** | Data on the following outcomes will be evaluated to compare values and trends in the period before and after the onset of the COVID-19 pandemic.   1. Body Mass Index - (to be calculated from most height and weight and/or measures independently entered as BMI). 2. Height to derive BMI – most recent adult height will be used, even if this was recorded prior to the study period. 3. Weight – all weights during the study periods will be recorded. 4. Systolic and Diastolic Blood Pressure (Home and Clinic readings). 5. Lipid profile: Total cholesterol, LDL cholesterol, HDL cholesterol, triglycerides 6. HbA1c 7. Smoking status 8. Referral to a weight management programme |
| **POPULATION** | All adults living in the UK and registered with a GP using electronic health records provided by The Phoenix Partnership (TPP) or Egerton Medical Information System (EMIS) on the 1^st^ March 2020. |
| **duration** | Routine data will be collected over the following time periods:  Pre-COVID: 1st March 2015 – 1^st^ March 2020  Post-COVID: 1^st^ March 2020 to the point of data analysis |
|  |  |

# INTRODUCTION

This analysis will investigate how the coronavirus pandemic has impacted the weight and metabolic risk factors of adults living in the UK and which groups have experienced the greatest change. This will identify specific groups of people that should be targeted by NHS-provided weight loss interventions following the onset of the coronavirus pandemic.

Lockdowns, stay at home orders and social distancing guidelines have had an impact on the lifestyles of people living in the UK. Studies have shown a reduction in physical activity during the pandemic, using a variety of data sources such as smartphone apps (McCarthy, Potts et al. 2021), recordings from implantable cardiac devices (Taylor, Ndiaye et al. 2021) and surveys. (Hillyard, Sinclair et al. 2021) Some international surveys have reported that lifestyle changes during the pandemic have resulted in weight gain and increased Body Mass Index (BMI) (a measure of if you are a healthy weight for your height)(Urzeala, Duclos et al. 2021). In the UK people joining the NHS Diabetes Prevention Program, a targeted intervention for people at high risk of diabetes, in the year following the pandemic were on average heavier than those who joined the programme in the years before the pandemic.(Valabhji, Barron et al.) But there is a lack of data on how the weight (and consequently BMI) of the general population of adults living in the UK has changed since the onset of the pandemic.

Even prior to the pandemic, obesity was common in the UK. The 2019 Health Survey for England (NHS Digital Lifestyles Team 2020) demonstrated that 64% of adults were overweight or obese. A study looking at wights measured in routinely collected GP records between 1998 and 2016 showed that a large proportion of adults moved from a ‘healthy weight’ to ‘overweight’ over a ten year period. (Katsoulis, Lai et al.) Obesity has long been known to be a risk factor for the development of a range of conditions such as diabetes, heart disease and stroke. This can be through the effect of obesity on established markers of metabolic risk such as: blood pressure (BP), blood lipid profile, and chronic levels measured by Haemoglobin A1c (HbA1c). Furthermore, obesity, and conditions linked to obesity such as diabetes, have been shown to increase the risk of hospitalisation and/or death from COVID-19. (Williamson, Walker et al. 2020)

In response to these challenges the NHS has invested in a range of interventions to support weight loss. Such as the NHS England Digital Weight Management Programme (DWMP), which is currently available to help overweight and obese people living with diabetes and/or hypertension manage their weight. This analysis will look at how the number of people meeting the eligibility criteria for the DWMP has changed since the onset of the pandemic through assessing BMI changes in these groups. We will also describe, amongst all adults living in the UK, how BMI and metabolic markers have changed since the onset of the pandemic, in order to assess the need for weight loss interventions in the overall population.

We will further investigate whether some groups of people have, since the onset of the pandemic, experienced more weight gain (and resultant increase in BMI) and/or worsening of their metabolic markers than others. These groups will be identified based on personal characteristics (such as age, sex, ethnicity, deprivation and/or region of residence) and whether they have another co-existent medical condition, such as asthma or chronic kidney disease. This will demonstrate specific population groups that may most benefit from targeted weight loss interventions. We will specifically look at co-existing medical conditions that have been deemed common and important by the NHS Qualify Outcome Framework (QOF) - a tool that is used to assess the quality of care provided by GPs. (National Institute for Health and Care Excellence)

The findings of this analysis will help inform NHS England health care policy around the extent of need for specific weight loss interventions and which specific groups are at greatest need of these interventions. As such this study answers important questions about the impact of the COVID pandemic on the health of the population and provides important data that will guide NHS service recovery. We have worked closely with key stakeholders, including the NHS England Clinical Director for Diabetes and Obesity, to develop a research project that answers clinically relevant research questions that can inform evidence based planning of interventions.

There is, to our knowledge, no available data answering these question. The analysis will be conducted through the OpenSAFELY platform which allows near real time access to routine electronic health records (EHRs) across over 98% of adults registered with a GP in the UK (further details of this platform are provided elsewhere in this application). As such this platform allows a more robust and complete analysis of UK health data than is currently available through any other platform or research database.

# STUDY OBJECTIVES

Aim: To evaluate, amongst adults living in the UK, how measures of healthy weight (such as Body Mass Index) and metabolic risk factors (such as blood pressure (BP), blood lipid profiles, chronic blood glucose levels measured by Haemoglobin A1c (HbA1c) and smoking status) have changed since the onset of the COVID-19 pandemic using data in routinely collected GP electronic health records (EHRs)

Objectives:

**Objective 1:** To investigate whether some groups of people have, since the onset of the pandemic, experienced more weight gain (and resultant increase in BMI) and/or worsening of their metabolic risk factors than others based on:

1. their personal characteristics (such as age, sex, ethnicity, deprivation and/or region of residence)
2. whether they are living with a chronic disease such as asthma, diabetes or chronic kidney disease have experienced more weight gain and worsening of their metabolic markers. Specifically considering the chronic diseases deemed common and important in the NHS Quality Outcomes Framework – a tool used to assess the quality of care provided by GPs.

**Objective 2:** To evaluate how the COVID-19 pandemic has affected the number of adults living in the UK eligible for targeted weight loss interventions, such as the [NHS Digital Weight Management Programme](https://www.england.nhs.uk/digital-weight-management/) (which is currently accessible to individuals with Diabetes or Hypertension and a BMI> 30 (or BMI > 27.5 for Black, Asian or Ethnic Minority backgrounds due to their increased risk of disease at a lower BMI)).

**Objective 3:** To identify which populations, based on demographic data and comorbidities, have experienced the greatest increase in weight and/or have the highest prevalence of obesity and should thus be targeted for weight loss interventions in the initial period of service restoration.

**Objective 4:** To assess the number of referrals to established weight loss programmes and whether there is a difference between groups of people on whether they are likely to be referred based on (i) the personal characteristics listed in Objective 1 above; and (ii) whether they are living with the chronic diseases discussed in Objective 1 above.

# Method

This will be a population based cohort analysis. Patients will be followed from 1^st^ March 2015 (5 years before the onset of the pandemic) till the point of data analysis, to evaluate how trends in BMI and metabolic markers have changed since the onset of the COVID-19 pandemic (1^st^ March 2020).

## Data Source: OpenSAFELY

We will use data from general practice (GP) records, obtained from the Electronic Health Record Providers, The Phoenix Partnership (TPP) and Egerton Medical Information System (EMIS), who provide GPs with the software and platform to create and store EHRs. Over 98% of patients in the UK registered with a GP have their primary care records stored through one of these providers. Data from the Hospital Episode Statistics will also be used to get further information on ethnicity, which can be poorly recorded in the primary care record.

All data will be accessed, linked and analysed through the OpenSAFELY platform - a new data analytics platform created to address urgent questions related to the Coronavirus Pandemic.

OpenSAFELY software, in the form of OpenSAFELY-TPP and OpenSAFELY-EMIS is hosted in the secure data environments of TPP and EMIS respectively. OpenSAFELY provides a secure software interface that allows NHS records to be pseudonymised, linked and analysed in near real-time; the GP patient data held never leaves the secure data environment of TPP or EMIS; other datasets are linked to it. TPP and EMIS , are Tier 3 data centres, accredited to NHS Digital standards for centrally hosted clinical systems (ISO 27001 standard and IG Toolkit version 2).

The OpenSAFELY platform allows researchers to develop analytical code using dummy data that has the same structure as the real data, but does not contain any real patient information. Once code related to data management and analysis has been developed it is run by the OpenSAFELY team in the OpenSAFELY-EMIS and OpenSAFELY-TPP. The researcher is then able to access aggregate outputs in the form of tables and charts without having access the real patient data. All outputs are checked for disclosure risks prior to release from the OpenSAFELY platform to the wider research group.

The OpenSAFELY model, allows the research team to perform an analysis of routinely collected data, without any access to the original source data.

## Study Population

Population size: The OpenSAFELY platform provides access to all patients registered with a GP surgery using TPP or EMIS Electronic Health Record software. A recent analysis in the OpenSAFELY platform demonstrated that gave access to 58 million patient records.(Walker, MacKenna et al. 2021)

Inclusion Criteria

1. Registration with a GP surgery, on the 1^st^ March 2020, that uses EMIS or TPP software to record and store electronic health records
2. All adults: aged >18 and <110 on the 1^st^ March 2020
3. Recorded female or male sex on the 1^st^ March 2020

Exclusion Criteria

1. Patients will less than 12 months primary care data available on the 1^st^ March 2020

(new registrations in the 12 months prior to 1^st^ March 2020 will be excluded from the analysis as there are often delays in the transfer of health records between practices).

## Study Measures

**Study Outcomes**

This analysis will evaluate how the values and trends in the following outcomes changed since the onset of the COVID-19 pandemic:

1. Body Mass Index - (to be calculated from most height and weight and/or measures independently entered as BMI).
2. Height to derive BMI – most recent adult height will be used, even if this was recorded prior to the study period.
3. Weight – all weights during the study periods will be recorded.
4. Systolic and Diastolic Blood Pressure (Home and Clinic readings).
5. Lipid profile: Total cholesterol, LDL cholesterol, HDL cholesterol, triglycerides
6. HbA1c
7. Smoking status
8. Referral to a weight management programme

**Study Exposures**

This is a time series analysis that will compare values and trends pre-COVID to those following the onset of COVID (with the date 1^st^ March 2020 considered the onset of the COVID pandemic).

**Covariates**

Stratified analysis will be undertaken to assess the impact of the COVID pandemic based on variations in the following:

1. Age
2. Sex
3. Ethnicity recorded in primary care records or linked HES data.
4. Index of Multiple Deprivation (IMD)
5. Geographic Data
6. The presence of Comorbidities - existing and new diagnoses of Quality Outcomes Framework (QOF) conditions
   1. Atrial Fibrillation
   2. Coronary Heart Disease
   3. Heart Failure
   4. Hypertension
   5. Peripheral Arterial Disease
   6. Stroke and TIA
   7. Diabetes Mellitus
   8. Asthma
   9. Chronic Obstructive Pulmonary Disease
   10. Dementia
   11. Depression
   12. Mental Health
   13. Cancer
   14. Chronic Kidney Disease
   15. Epilepsy
   16. Learning Disabilities
   17. Osteoporosis
   18. Rheumatoid Arthritis
   19. Palliative Care
   20. Non diabetic hyperglycaemia
7. Date of registered live birth or still birth – to identify outcome data in the prenatal or postnatal periods which should be excluded from the analysis.

QOF conditions *have been included as a covariate, to identify whether patients living with specific comorbidities such have experienced greater need for targeted weight loss interventions. We have chosen to look at comorbidities covered by QOF as these have been benchmarked as common and/or important by NICE, and are likely to be well-coded in the EHR.*

## Study Duration

Outcome data will be collected over the following time periods:

Pre-covid period: 1^st^ March 2015- 1^st^ March 2020

This will be included to identify baseline levels of the outcomes in the population and underlying trends prior to the onset of the pandemic.

Post-covid period: 1^st^ March 2020 – point of data extraction

This will allow an assessment of how values and trends varied in the periods post covid compared to the pre-covid period

# STATISTICs and data analysis

1. Primary care data is entered by health care professionals into the Electronic Health Records (EHRs) as a combination of free text and clinical codes which specify specific diseases and disease processes. Best practice of record keeping is to ensure that clinical codes are entered in addition to free text so EHRs can be rapidly searched and reviewed for clinical, audit and research purposes. More than one code can be used to represent a single clinical condition or measurement, therefore ‘code lists’ are required to identify all the relevant information.
2. This analysis will be limited to data entered as clinical codes Specific code lists will be developed to identify data related to each of the study outcomes, exposures and covariates listed above. Where established code lists are available they will be reviewed and curated to ensure the data being collected are aligned with the specific analysis being performed. Such code lists may be available from previous studies researching the same conditions or aligned with care processes, such as the Quality Outcome Framework ( a national system of monitoring the quality of GP care processes in relation to common and important chronic diseases such as diabetes, hypertension and asthma).
3. Data will be analysed using STATA.
4. Descriptive statistics will be used to describe the numbers and character of the population who are having each of the outcome measures and how this varied in the period before and after the onset of the pandemic. This will help describe any bias introduced by different health care practices in the period before and after the pandemic in terms of which individuals were having the outcomes measures.
5. Changes in the values and trends of each of the outcomes listed above will be assess on a population level and individual level in time series analysis standardised for age and sex using an appropriate model based on the character of the data.
6. Further analyses will assess how population level trends in each of the outcomes have changed since the onset of the pandemic, after standardisation for age and sex, using an appropriate model based on the character of the data.
7. Multilevel modelling, accounting for clustering at the level of the Primary Care Network will be used to evaluate whether these outcomes varied by the demographic subgroups or comorbidities listed above.
8. Rates of referral to weight management programmes and variation by demographic groups and co-morbidity listed above will be assessed to identify current uptake of an intervention.
9. Missing exposure and covariate data (such as ethnicity) is likely to be Missing Not At Random. Missing data will be described and an assessment will be made whether to exclude individuals with missing for significant covariates such as ethnicity or IMD from the regression analyses, to consider those with missing values as a separate category or to use multiple imputation techniques.
10. To reduce the impact of pregnancy-associated changes on the analysis – outcome data entered in the six months before or after a live birth or still birth will be excluded from the analysis.

Data and all appropriate documentation will be stored for a minimum of 5 years after the completion of the study, including the follow-up period.

# Risks and Benefits

This is a secondary analysis of routinely entered in electronic health records. As such there is no change to routine treatment of patients. As will all analysis related to health records the main risks, related to data security and confidentiality, are discussed below.

## Data security

The analysis is using routinely collected Electronic Health Records. Therefore the greatest risk is ensuring data security is maintained and patient confidentiality is not breached during the process of analysis or in the outputs of analysis. This research will be undertaken using the OpenSAFELY platform which has unique features that help ensure data security. Many other research databases extract pseudo anonymised data from electronic health records which researchers are able to access for the purposes of specified analysis. OpenSAFELY has worked in collaboration with the two major GP EHR providers, EMIS and TPP, to develop OpenSAFELY-EMIS and OpenSAFELY-TPP. This software is run within the secure data environments of EMIS and TPP and allows the researcher to run specific statistical analyses which answer the research questions without ever accessing the raw data. In the OpenSAFELY platform, researchers develop their analytic code using ‘dummy data’ which is generated to match the structure of the ‘real data’ but does not contain any real patient information. When the code has been developed the OpenSAFELY platform allows this code to be run in the secure data warehouses (EMIS and TPP) that already holds the Electronic Health Records of over 98% of patients registered with a GP. The platform then produces outputs in the form of tables and graphs that do not contain unique identifiable information. All outputs are reviewed for possible disclosures (due to small cells) prior to being released from the OpenSAFELY platform to the wider research team. As such the analysis will be undertaken without the research teams every being able to directly access the raw data.

## Confidentiality

As described above the research is undertaken without the researcher ever having access to raw patient data. Furthermore before the outputs of analysis are released from the open safely platform they undergo a disclosure review, to check there are no tables containing any cells with a low number of participants or graphs with point estimates that could identify individual patients. As this analysis is looking at population trends of weight and metabolic risk factors across the whole of the UK, it is unlikely that any of the outputs generated will risk disclosing individual patient information. The primary researcher has undertaken safe researcher training by the UK data service, which has provided further training in the use of disclosure controls when working with big data.

## Risk of bias in the results

This analysis is using routinely collected data from electronic health record of patients attending their GP surgery. This is dependent on a patient being in contact with the GP surgery and the health care professional in the GP surgery measuring the outcome of interest (such as the patient’s BMI). As such, there are likely to be differences between people who have needed to attend their GP and have these outcomes measured compared to those who have not. Therefore we will need to be clear when we interpret and publish the data that the findings from this analysis may not represent that of the general population. Additionally we are assessing whether trends and values in the recorded BMI and metabolic parameters have changed since the onset of the pandemic, as access to care and care provision has changed during the pandemic, this could introduce further bias in terms of who is having these measures taken in the post pandemic period and whether changes seen in this group represent the wider population of patients attending their GP (but not having BMI or other metabolic parameters assessed). We will carefully explore these limitation in the conduct of the study (through assessing the numbers and characteristics of individuals who are having outcomes measured before and after the onset of the pandemic) and interpret the findings transparently to highlight the risk of any bias. However, this analysis will provide an indication for the need for weight loss interventions based on patients attending their GP. Furthermore, we hope the findings will inform further research.

# regulatory issues including Ethics

The OpenSAFELY platform was approved to perform research related to the COVID-19 pandemic by the Health Research Authority (REC reference 20/LO/0651) and by the LSHTM Ethics Board (reference 21863).

NHS England is the data controller; TPP and EMIS are the data processor; and the key researchers on OpenSAFELY are acting on behalf of NHS England. This implementation of OpenSAFELY is hosted within the TPP and EMIS environments which are accredited to the ISO 27001 information security standard and are NHS IG Toolkit compliant; patient data has been pseudonymised for analysis and linkage using industry standard cryptographic hashing techniques; all pseudonymised datasets transmitted for linkage onto OpenSAFELY are encrypted; access to the platform is via a virtual private network (VPN) connection, restricted to a small group of researchers, their specific machine and IP address; the researchers hold contracts with NHS England and only access the platform to initiate database queries and statistical models; all database activity is logged; only aggregate statistical outputs leave the platform environment following best practice for anonymisation of results such as statistical disclosure control for low cell counts. The OpenSAFELY research platform adheres to the data protection principles of the UK Data Protection Act 2018 and the EU General Data Protection Regulation (GDPR) 2016. In March 2020, the Secretary of State for Health and Social Care used powers under the UK Health Service (Control of Patient Information) Regulations 2002 (COPI) to require organisations to process confidential patient information for the purposes of protecting public health, providing healthcare services to the public and monitoring and managing the COVID-19 outbreak and incidents of exposure. Taken together, these provide the legal bases to link patient datasets on the OpenSAFELY platform

## Confidentiality

This analysis will be conducted through the OpenSAFELY platform. As discussed above this platform allows researchers to develop statistical code using dummy data (without accessing real patient information). The code is then run by the OpenSAFELY team in the secure data environments of EMIS and TPP to produce aggregate outputs for review by the research team, after disclosure controls have been introduced. As such, no patient identifiable information will be accessed by the research team.

## Funding

The analysis will be undertaken by MS – an NIHR-funded Academic Clinical Fellow in primary care., supported by the rest of the research team in their academic time There is no fee attached to the use of the OpenSAFELY platform.

## PROTOCOL DEVELOPMENT

This protocol was developed by the following investigators who are responsible for the development of, and agreeing to, the final protocol. Subsequent changes to the final protocol will require the agreement of the TSC. Dr Miriam Samuel, Dr Rohini Mathur, Dr Sarah Finer.

# Publication Policy

*All findings of the study will be communicated through publication in peer-reviewed journals. Authorship of the publications will reflect involvement in the study.*

# References

Hillyard, M., M. Sinclair, M. Murphy, K. Casson and C. Mulligan (2021). "The impact of COVID-19 on the physical activity and sedentary behaviour levels of pregnant women with gestational diabetes." PLoS One **16**(8): e0254364.

Katsoulis, M., A. G. Lai, K. Diaz-Ordaz, M. Gomes, L. Pasea, A. Banerjee, S. Denaxas, K. Tsilidis, P. Lagiou, G. Misirli, K. Bhaskaran, G. Wannamethee, R. Dobson, R. L. Batterham, D.-K. Kipourou, R. T. Lumbers, L. Wen, N. Wareham, C. Langenberg and H. Hemingway "Identifying adults at high-risk for change in weight and BMI in England: a longitudinal, large-scale, population-based cohort study using electronic health records." The Lancet Diabetes & Endocrinology: 681-694.

McCarthy, H., H. W. W. Potts and A. Fisher (2021). "Physical Activity Behavior Before, During, and After COVID-19 Restrictions: Longitudinal Smartphone-Tracking Study of Adults in the United Kingdom." J Med Internet Res **23**(2): e23701.

National Institute for Health and Care Excellence. "NICE Quality and Outcome Framework." Retrieved 7th September 2021, from <https://www.nice.org.uk/standards-and-indicators/qofindicators?categories=&page=1>.

NHS Digital Lifestyles Team. (2020). "Health Survey for England 2019." Retrieved 7th September 2021, from <https://digital.nhs.uk/data-and-information/publications/statistical/health-survey-for-england/2019>.

Taylor, J. K., H. Ndiaye, M. Daniels and F. Ahmed (2021). "Lockdown, slow down: impact of the COVID-19 pandemic on physical activity—an observational study." Open Heart **8**(1): e001600.

Urzeala, C., M. Duclos, U. Chris Ugbolue, A. Bota, M. Berthon, K. Kulik, D. Thivel, R. Bagheri, Y. Gu, J. S. Baker, N. Andant, B. Pereira, K. Rouffiac, M. Clinchamps and F. Dutheil (2021). "COVID-19 lockdown consequences on body mass index and perceived fragility related to physical activity: A worldwide cohort study." Health Expect.

Valabhji, J., E. Barron, D. Bradley, C. Bakhai, K. Khunti and S. Jebb "Effect of the COVID-19 pandemic on body weight in people at high risk of type 2 diabetes referred to the English NHS Diabetes Prevention Programme." The Lancet Diabetes & Endocrinology.

Walker, A. J., B. MacKenna, P. Inglesby, L. Tomlinson, C. T. Rentsch, H. J. Curtis, C. E. Morton, J. Morley, A. Mehrkar, S. Bacon, G. Hickman, C. Bates, R. Croker, D. Evans, T. Ward, J. Cockburn, S. Davy, K. Bhaskaran, A. Schultze, E. J. Williamson, W. J. Hulme, H. I. McDonald, R. Mathur, R. M. Eggo, K. Wing, A. Y. S. Wong, H. Forbes, J. Tazare, J. Parry, F. Hester, S. Harper, S. O’Hanlon, A. Eavis, R. Jarvis, D. Avramov, P. Griffiths, A. Fowles, N. Parkes, I. J. Douglas, S. J. W. Evans, L. Smeeth and B. Goldacre (2021). "Clinical coding of long COVID in English primary care: a federated analysis of 58 million patient records &lt;em&gt;in situ&lt;/em&gt; using OpenSAFELY." British Journal of General Practice: BJGP.2021.0301.

Williamson, E. J., A. J. Walker, K. Bhaskaran, S. Bacon, C. Bates, C. E. Morton, H. J. Curtis, A. Mehrkar, D. Evans, P. Inglesby, J. Cockburn, H. I. McDonald, B. MacKenna, L. Tomlinson, I. J. Douglas, C. T. Rentsch, R. Mathur, A. Y. S. Wong, R. Grieve, D. Harrison, H. Forbes, A. Schultze, R. Croker, J. Parry, F. Hester, S. Harper, R. Perera, S. J. W. Evans, L. Smeeth and B. Goldacre (2020). "Factors associated with COVID-19-related death using OpenSAFELY." Nature **584**(7821): 430-436.
