# Supplementary material for: Trends in weight gain recorded in English primary care before and during the Coronavirus-19 pandemic: An observational cohort study using the OpenSAFELY platform
Source: PLoS Med. 2024 Jun 24;21(6):e1004398. doi: 10.1371/journal.pmed.1004398 (PMC11249215; doi:10.1371/journal.pmed.1004398)
Supplement: S1 Appendix — (DOCX) [file pmed.1004398.s003.docx]

## S1 Appendix. Information Governance Statement

NHS England is the data controller for OpenSAFELY-TPP; TPP is the data processor; all study authors using OpenSAFELY have the approval of NHS England. This implementation of OpenSAFELY is hosted within the TPP environment which is accredited to the ISO 27001 information security standard and is NHS IG Toolkit compliant. Patient data has been pseudonymised for analysis and linkage using industry standard cryptographic hashing techniques; all pseudonymised datasets transmitted for linkage onto OpenSAFELY are encrypted; access to the platform is via a virtual private network (VPN) connection, restricted to a small group of researchers; the researchers hold contracts with NHS England and only access the platform to initiate database queries and statistical models; all database activity is logged; only aggregate statistical outputs leave the platform environment following best practice for anonymisation of results such as statistical disclosure control for low cell counts. The OpenSAFELY research platform adheres to the obligations of the UK General Data Protection Regulation (GDPR) and the Data Protection Act 2018. In March 2020, the Secretary of State for Health and Social Care used powers under the UK Health Service (Control of Patient Information) Regulations 2002 (COPI) to require organisations to process confidential patient information for the purposes of protecting public health, providing healthcare services to the public and monitoring and managing the COVID-19 outbreak and incidents of exposure; this sets aside the requirement for patient consent. This was extended in November 2022 for the NHS England OpenSAFELY COVID-19 research platform. In some cases of data sharing, the common law duty of confidence is met using, for example, patient consent or support from the Health Research Authority Confidentiality Advisory Group. Taken together, these provide the legal bases to link patient datasets on the OpenSAFELY platform. GP practices, from which the primary care data are obtained, are required to share relevant health information to support the public health response to the pandemic, and have been informed of the OpenSAFELY analytics platform. This study was supported by Dr Jonathan Valabhji, clinical director for diabetes and obesity at NHS England, as senior sponsor.

1. Data Security and Protection Toolkit - NHS Digital. NHS Digital. [**https://digital.nhs.uk/data-and-information/looking-after-information/data-security-and-information-governance/data-security-and-protection-toolkit**](https://digital.nhs.uk/data-and-information/looking-after-information/data-security-and-information-governance/data-security-and-protection-toolkit) (accessed 30 Apr 2020). [**↩︎**](https://www.opensafely.org/policies-for-researchers/#fnref:1)
2. ISB1523: Anonymisation Standard for Publishing Health and Social Care Data - NHS Digital. NHS Digital. [**https://digital.nhs.uk/data-and-information/information-standards/information-standards-and-data-collections-including-extractions/publications-and-notifications/standards-and-collections/isb1523-anonymisation-standard-for-publishing-health-and-social-care-data**](https://digital.nhs.uk/data-and-information/information-standards/information-standards-and-data-collections-including-extractions/publications-and-notifications/standards-and-collections/isb1523-anonymisation-standard-for-publishing-health-and-social-care-data) (accessed 30 Apr 2020). [**↩︎**](https://www.opensafely.org/policies-for-researchers/#fnref:2)
3. Secretary of State for Health and Social Care - UK Government. Coronavirus (COVID-19): notification to organisations to share information. 2020. [**https://web.archive.org/web/20200421171727/https://www.gov.uk/government/publications/covid-19-notification-to-gps-and-nhs-england-to-share-information**](https://web.archive.org/web/20200421171727/https://www.gov.uk/government/publications/covid-19-notification-to-gps-and-nhs-england-to-share-information) [**↩︎**](https://www.opensafely.org/policies-for-researchers/#fnref:3)
4. Secretary of State for Health and Social Care - UK Government. Coronavirus (COVID-19): notification to organisations to share information. 2022. [**https://www.gov.uk/government/publications/coronavirus-covid-19-notification-to-organisations-to-share-information/coronavirus-covid-19-notice-under-regulation-34-of-the-health-service-control-of-patient-information-regulations-2002**](https://www.gov.uk/government/publications/coronavirus-covid-19-notification-to-organisations-to-share-information/coronavirus-covid-19-notice-under-regulation-34-of-the-health-service-control-of-patient-information-regulations-2002) [**↩︎**](https://www.opensafely.org/policies-for-researchers/#fnref:4)
5. Confidentiality Advisory Group. Health Research Authority. [**https://www.hra.nhs.uk/about-us/committees-and-services/confidentiality-advisory-group/**](https://www.hra.nhs.uk/about-us/committees-and-services/confidentiality-advisory-group/) [**↩︎**](https://www.opensafely.org/policies-for-researchers/#fnref:5)
