# Supplementary material for: Trends in weight gain recorded in English primary care before and during the Coronavirus-19 pandemic: An observational cohort study using the OpenSAFELY platform
Source: PLoS Med. 2024 Jun 24;21(6):e1004398. doi: 10.1371/journal.pmed.1004398 (PMC11249215; doi:10.1371/journal.pmed.1004398)
Supplement: S2 Appendix — (DOCX) [file pmed.1004398.s004.docx]

## S2 Appendix Calculation of δ-prepandemic, δ-pandemic and δ-change

Body Mass Index (BMI) data were classified into time periods: period-1: March 2015 - February 2018; period-2: March 2018 - February 2020; and period-3 March 2020 - February 2022. For patients with more than one BMI measure from each time period, a random BMI measure was selected from each time period. Where data were available, BMI data from period 1 and period 2 were used to calculate δ-prepandemic (e.g. dummy patient 1), while BMI data from period 2 and period 3 were used to calculate δ-pandemic (e.g dummy patient 1 and dummy patient 2). Rate of BMI change/year was calculated between these time points assuming a linear trend [(Katsoulis, Lai, et al. 2021)](https://paperpile.com/c/nR0i9Z/fDXt7).

To assess the impact of the pandemic in rate of weight gain, we calculated delta change as the change in rate of weight gain between the prepandemic and pandemic periods (δ-change = δ-pandemic - δ-prepandemic). Patients therefore required both a δ-prepandemic and δ-pandemic value to contribute to the δ-change analysis (e.g., dummy patient 1). Patients with either a δ-prepandemic or δ-pandemic, but not both, contributed to the prepandemic or pandemic BMI trajectory analysis respectively, but not to the δ-change analysis (e.g., dummy patient 2). Patients without BMI data from period 2 were unable to contribute to any of these analyses (e.g. dummy patient 3).

For covariate categories that could change for an individual during the course of the study (age group, IMD quintile, long-term condition) individuals were assigned based on their characteristics at the following time points: δ-prepandemic = Mar 2015, δ-pandemic = March 2018, δ-change = February 2022.

## Figure. Demonstration of calculation of δ-prepandemic, δ-pandemic and δ-change using dummy patient data


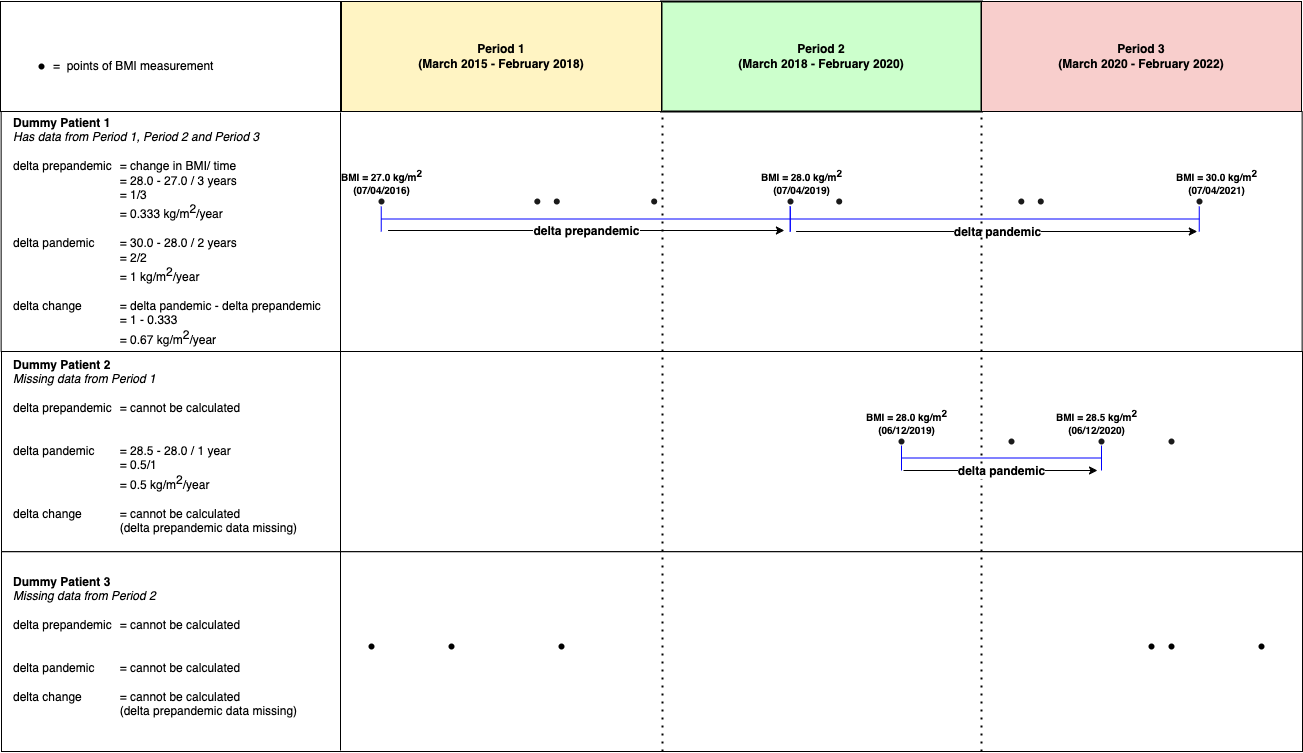


## 
