# Supplementary material for: Trends in weight gain recorded in English primary care before and during the Coronavirus-19 pandemic: An observational cohort study using the OpenSAFELY platform
Source: PLoS Med. 2024 Jun 24;21(6):e1004398. doi: 10.1371/journal.pmed.1004398 (PMC11249215; doi:10.1371/journal.pmed.1004398)
Supplement: S3 Appendix — (DOCX) [file pmed.1004398.s005.docx]

## S3 Appendix. Defining a population of extreme accelerators (distribution of δ-change)

We plotted the distribution of δ-change in the 2,768,695 individuals contributing to the analysis of change in rate of weight gain after the onset of the pandemic (Figure). We identified individuals with a δ-change ≥ 1.84 kg/m^2^/year as the ten percent of the population experiencing the most extreme acceleration in their rate of weight gain during the pandemic and explored the distribution of extreme accelerators in different subgroups of the study population.

As extreme accelerators were defined as the top-decile of estimated δ-change in the total study population, clinical and sociodemographic subgroups in which over ten percent of individuals were extreme accelerators had an increased prevalence of extreme acceleration, conversely subgroups in which less than ten percent were extreme accelerators had a reduced prevalence.

## Figure. Cumulative distribution of change in rate of weight gain (δ-change) amongst adults living in England before and after the onset of the COVID-19 pandemic.


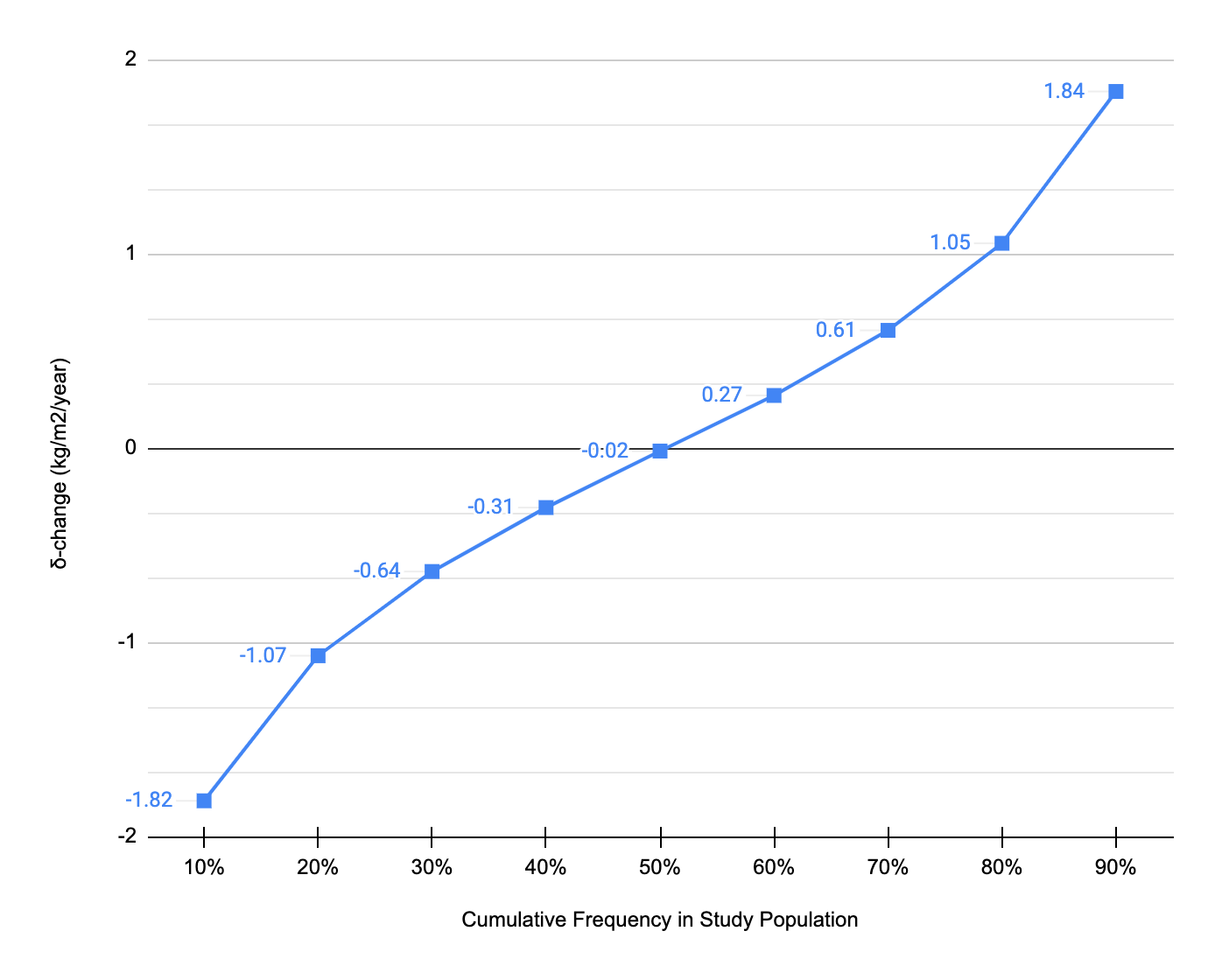


## 
