# Supplementary material for: Trends in weight gain recorded in English primary care before and during the Coronavirus-19 pandemic: An observational cohort study using the OpenSAFELY platform
Source: PLoS Med. 2024 Jun 24;21(6):e1004398. doi: 10.1371/journal.pmed.1004398 (PMC11249215; doi:10.1371/journal.pmed.1004398)
Supplement: S1 Table — (DOCX) [file pmed.1004398.s006.docx]

| S1 Table: Average Body Mass Index (BMI) recorded in the routine healthcare records of adults living in England before and after the onset of the pandemic. | | | | | | |
| --- | --- | --- | --- | --- | --- | --- |
|  | Year beginning March 2019  (Prepandemic Year) | | Year beginning March 2020  (Pandemic Year 1) | | Year beginning March 2021  (Pandemic Year 2) | |
|  | N (%) | Median BMI (IQR) | N (%) | Median BMI (IQR) | N (%) | Median BMI (IQR) |
| Total | 5,094,590 | 27.8 (24.3, 32.1) | 3,173,970 | 27.9 (24.2, 32.4) | 4,422,295 | 28.0 (24.4, 32.6) |
| Sex |  |  |  |  |  |  |
| Female | 2,943,330 (57.8) | 27.4 (23.6, 32.3) | 1,871,025 (58.9) | 27.5 (23.6, 32.7) | 2,591,225 (58.6) | 27.7 (23.8, 32.9) |
| Male | 2,151,260 (42.2) | 28.1 (25.1, 31.8) | 1,302,945 (41.1) | 28.3 (25.1, 32.1) | 1,831,065 (41.4) | 28.4 (25.2, 32.1) |
| Age Group (years) |  |  |  |  |  |  |
| 18-29 | 580,335 (11.4) | 25.2 (21.9, 30.2) | 409,920 (20.9) | 25.1 (21.9, 30.1) | 454,245 (10.3) | 25.4 (22.0, 30.8) |
| 30-39 | 579,610 (11.4) | 27.1 (23.5, 32.1) | 385,130 (19.6) | 27.3 (23.6, 32.5) | 469,950 (10.6) | 27.8 (23.9, 33.2) |
| 40-49 | 735,120 (14.4) | 28.2 (24.6, 32.9) | 417,080 (21.3) | 28.6 (24.8, 33.7) | 591,250 (13.4) | 28.7 (24.8, 33.6) |
| 50-59 | 958,860 (18.8) | 28.9 (25.4, 33.4) | 558,625 (28.5) | 29.4 (25.6, 34.1) | 823,905 (18.6) | 29.3 (25.6, 34.0) |
| 60-69 | 980,770 (19.3) | 28.5 (25.2, 32.6) | 578,445 (29.5) | 28.9 (25.4, 33.2) | 839,940 (19.0) | 28.9 (25.4, 33.2) |
| 70-79 | 885,645 (17.4) | 27.7 (24.7, 31.4) | 561,970 (28.6) | 27.8 (24.7, 31.6) | 836,005 (18.9) | 27.8 (24.6, 31.6) |
| 80-90 | 374,250 (7.3) | 26.3 (23.5, 29.6) | 262,795 (13.4) | 26.2 (23.3, 29.4) | 406,995 (9.2) | 26.2 (23.2, 29.6) |
| Patient IMD Quintile |  |  |  |  |  |  |
| 1 (most deprived) | 1,011,785 (20.2) | 28.7 (24.8, 33.5) | 636,765 (20.5) | 29.0 (24.9, 33.9) | 868,410 (20.1) | 29.1 (25.1, 34.1) |
| 5 (least deprived) | 905,350 (18.1) | 27.0 (23.9, 30.9) | 552,035 (17.8) | 27.0 (23.7, 31.0) | 777,720 (18.0) | 27.1 (23.9, 31.2) |
| Missing | 89,270 (1.8) | 27.8 (24.2, 32.2) | 65,980 (5.3) | 27.9 (24.2, 32.4) | 108,220 (2.4) | 27.9 (24.3, 32.5) |
| Ethnicity |  |  |  |  |  |  |
| White British | 4,017,955 (81.9) | 27.9 (24.4, 32.2) | 2,480,500 (81.1) | 28.0 (24.4, 32.6) | 3,446,610 (81.1) | 28.2 (24.5, 32.8) |
| White Irish | 27,760 (0.6) | 27.4 (24.0, 31.3) | 17,460 (0.6) | 27.4 (23.9, 31.6) | 24,460 (0.6) | 27.5 (24.0, 31.8) |
| Other White | 293,165 (6.0) | 27.2 (23.6, 31.6) | 184,800 (6.0) | 27.2 (23.5, 31.8) | 254,570 (6.0) | 27.6 (23.8, 32.2) |
| Indian | 139,620 (2.8) | 26.8 (23.9, 30.3) | 91,605 (3.0) | 26.9 (23.9, 30.4) | 129,905 (3.1) | 26.8 (23.9, 30.4) |
| Pakistani | 111,395 (2.3) | 28.2 (24.9, 32.1) | 70,880 (2.3) | 28.4 (25.1, 32.5) | 99,095 (2.3) | 28.4 (25.1, 32.5) |
| Bangladeshi | 25,350 (0.5) | 26.6 (23.9, 29.8) | 17,330 (0.6) | 26.6 (23.9, 30.0) | 23,755 (0.6) | 26.6 (24.0, 29.9) |
| Chinese | 15,860 (0.3) | 23.7 (21.2, 26.6) | 9,570 (0.3) | 23.5 (21.1, 26.6) | 12,910 (0.3) | 23.8 (21.4, 26.8) |
| Other Asian | 65,660 (1.3) | 26.8 (23.8, 30.1) | 44,760 (1.5) | 26.9 (23.9, 30.4) | 62,390 (1.5) | 26.9 (23.9, 30.4) |
| Black Caribbean | 32,595 (0.7) | 28.7 (25.2, 33.1) | 21,190 (0.7) | 29.0 (25.4, 33.5) | 29,520 (0.7) | 29.1 (25.4, 33.6) |
| Black African | 49,330 (1.0) | 28.8 (25.4, 32.9) | 33,185 (1.1) | 29.0 (25.5, 33.2) | 46,435 (1.1) | 29.2 (25.7, 33.5) |
| Other Black | 22,845 (0.5) | 28.5 (25.0, 32.9) | 14,490 (0.5) | 28.7 (25.1, 33.4) | 21,265 (0.5) | 29.0 (25.3, 33.5) |
| White & Black Caribbean | 13,065 (0.3) | 28.0 (24.1, 32.9) | 8,905 (0.3) | 28.3 (24.3, 33.2) | 11,655 (0.3) | 28.5 (24.5, 33.7) |
| White & Black African | 8,375 (0.2) | 28.6 (24.8, 32.8) | 5,660 (0.2) | 28.7 (24.9, 33.1) | 7,720 (0.2) | 29.0 (25.1, 33.5) |
| White & Asian | 9,240 (0.2) | 26.3 (23.0, 30.4) | 6,250 (0.2) | 26.3 (22.8, 30.5) | 8,530 (0.2) | 26.6 (23.1, 30.8) |
| Other Mixed | 17,055 (0.3) | 27.1 (23.5, 31.5) | 11,705 (0.4) | 27.1 (23.4, 31.7) | 15,515 (0.4) | 27.5 (23.8, 32.2) |
| Other | 57,620 (1.2) | 27.2 (23.9, 31.2) | 39,790 (1.3) | 27.4 (24.0, 31.5) | 53,955 (1.3) | 27.5 (24.1, 31.6) |
| Missing | 187,700 (3.7) | 27.5 (23.9, 31.7) | 115,900 (3.7) | 27.2 (23.7, 31.6) | 174,020 (3.9) | 27.5 (23.9, 31.9) |
| Long Term Condition |  |  |  |  |  |  |
| Hypertension | 1,837,260 (36.1) | 29.2 (25.8, 33.4) | 1,234,855 (38.9) | 29.2 (25.7, 33.6) | 2,673,785 (39.5) | 29.2 (25.7, 33.6) |
| Type 1 Diabetes | 59,420 (1.2) | 26.9 (23.8, 30.5) | 41,440 (1.3) | 26.9 (23.7, 30.7) | 55,640 (1.3) | 27.1 (23.8, 30.9) |
| Type 2 Diabetes | 867,735 (17.0) | 30.4 (26.8, 34.8) | 669,295 (21.1) | 30.3 (26.7, 34.8) | 887,870 (20.1) | 30.3 (26.6, 34.8) |
| Cardiovascular Disease | 588,970 (11.6) | 28.5 (25.3, 32.5) | 407,485 (12.8) | 28.5 (25.2, 32.6) | 578,685 (13.1) | 28.5 (25.1, 32.5) |
| Learning Disability | 61,110 (1.2) | 28.4 (24.0, 33.7) | 51,200 (1.6) | 28.3 (23.9, 33.7) | 65,265 (1.5) | 28.5 (24.0, 34.0) |
| Depression | 1,233,900 (24.2) | 28.7 (24.8, 33.5) | 806,460 (25.4) | 29.0 (24.9, 34.1) | 1,140,545 (25.8) | 29.1 (25.0, 34.2) |
| Dementia | 48,460 (1.0) | 26.4 (23.0, 30.2) | 40,295 (1.3) | 25.9 (22.4, 29.9) | 60,785 (1.4) | 25.8 (22.3, 29.7) |
| Serious Mental Illness | 116,055 (2.3) | 28.6 (24.7, 33.3) | 88,595 (2.8) | 28.8 (24.8, 33.5) | 118,010 (2.7) | 28.9 (24.9, 33.6) |
| COPD | 308,030 (6.0) | 27.7 (23.9, 32.0) | 181,610 (5.7) | 28.0 (24.1, 32.5) | 261,870 (5.9) | 27.9 (23.9, 32.4) |
| Asthma | 1,040,870 (20.4) | 28.4 (24.6, 33.1) | 634,885 (20.0) | 28.6 (24.6, 33.7) | 871,240 (19.7) | 28.8 (24.8, 33.9) |
| Stroke and TIA | 226,455 (4.4) | 27.8 (24.6, 31.6) | 157,285 (5.0) | 27.7 (24.5, 31.7) | 229,915 (5.2) | 27.7 (24.4, 31.6) |
| Cancer | 348,585 (6.8) | 27.6 (24.4, 31.5) | 232,940 (7.3) | 27.7 (24.4, 31.6) | 348,225 (7.9) | 27.7 (24.3, 31.7) |
| BMI: measured in weight in kilograms/height in meters squared (kg/m^2^). N(%): total number (percentage) of population subgroup with a BMI recorded. IQR: Interquartile range. IMD: Index of Multiple Deprivation. COPD: Chronic Obstructive Pulmonary Disease. TIA: Transient Ischaemic Attack | | | | | | |
