# Supplementary material for: Trends in weight gain recorded in English primary care before and during the Coronavirus-19 pandemic: An observational cohort study using the OpenSAFELY platform
Source: PLoS Med. 2024 Jun 24;21(6):e1004398. doi: 10.1371/journal.pmed.1004398 (PMC11249215; doi:10.1371/journal.pmed.1004398)
Supplement: S2 Table — (DOCX) [file pmed.1004398.s007.docx]

## S2 Table: Proportion of adults living in England with a Body Mass Index (BMI) value recorded in their routine health care record in the period before and after the onset of the COVID-19 pandemic.

|  | Year beginning March 2019  (Prepandemic Year) | | | Year beginning March 2020  (Pandemic Year 1) | | | Year beginning March 2021  (Pandemic Year 2) | | |
| --- | --- | --- | --- | --- | --- | --- | --- | --- | --- |
|  | N | BMI recorded | | N | BMI recorded | | N | BMI recorded | |
|  |  | n | % (95% CI) |  | n | % (95% CI) |  | n | % (95% CI) |
| Total | 16,301,350 | 5,094,590 | 31·30 (31·28, 31·33) | 17,037,385 | 3,173,970 | 18·69 (18·67, 18·70) | 17,742,365 | 4,422,295 | 24·99 (24·97, 25·01) |
| Age Group (years) |  |  |  |  |  |  |  |  |  |
| 18-29 | 2,822,850 | 580,335 | 20·66 (20·61, 20·71) | 2,956,395 | 409,920 | 13·94 (13·90, 13·98) | 3,062,705 | 454,245 | 14·90 (14·86, 14·94) |
| 30-39 | 2,889,440 | 579,610 | 20·11 (20·06, 20·15) | 3,018,435 | 385,130 | 12·81 (12·77, 12·85) | 3,138,645 | 469,950 | 15·03 (14·99, 15·07) |
| 40-49 | 2,826,860 | 735,120 | 26·04 (25·99, 26·09) | 2,893,890 | 417,080 | 14·46 (14·42, 14·50) | 2,953,105 | 591,250 | 20·08 (20·03, 20·12) |
| 50-59 | 2,987,265 | 958,860 | 32·14 (32·08, 32·19) | 3,082,985 | 558,625 | 18·16 (18·12, 18·21) | 3,176,680 | 823,905 | 26·00 (25·95, 26·04) |
| 60-69 | 2,302,715 | 980,770 | 42·63 (42·56, 42·69) | 2,386,965 | 578,445 | 24·28 (24·23, 24·34) | 2,495,010 | 839,940 | 33·73 (33·67, 33·78) |
| 70-79 | 1,756,870 | 885,645 | 50·45 (50·37, 50·52) | 1,878,465 | 561,970 | 29·98 (29·91, 30·04) | 1,997,575 | 836,005 | 41·92 (41·85, 41·99) |
| 80-90 | 715,345 | 374,250 | 52·36 (52·25, 52·48) | 820,250 | 262,795 | 32·12 (32·02, 32·22) | 918,640 | 406,995 | 44·41 (44·31, 44·52) |
| Sex |  |  |  |  |  |  |  |  |  |
| Female | 8,180,085 | 2,943,330 | 36·04 (36·01, 36·07) | 8,538,025 | 1,871,025 | 21·98 (21·95, 22·01) | 8,883,720 | 2,591,225 | 29·25 (29·22, 29·28) |
| Male | 8,121,265 | 2,151,260 | 26·53 (26·50, 26·56) | 8,499,360 | 1,302,945 | 15·37 (15·35, 15·40) | 8,858,645 | 1,831,065 | 20·71 (20·69, 20·74) |
| Patient IMD Quintile |  |  |  |  |  |  |  |  |  |
| 1 (most deprived) | 3,102,370 | 1,011,785 | 32·67 (32·62, 32·72) | 3,240,870 | 636,765 | 19·72 (19·67, 19·76) | 3,356,720 | 868,410 | 25·95 (25·90, 26·00) |
| 5 (least deprived) | 3,029,530 | 905,350 | 29·93 (29·88, 29·98) | 3,133,315 | 552,035 | 17·67 (17·62, 17·71) | 3,240,120 | 777,720 | 24·05 (24·01, 24·10) |
| Missing | 273,985 | 89,270 | 32·62 (32·45, 32·80) | 345,395 | 65,980 | 19·16 (19·02, 19·29) | 431,235 | 108,220 | 25·16 (25·03, 25·29) |
| Ethnicity |  |  |  |  |  |  |  |  |  |
| White British | 11,590,555 | 4,017,955 | 34·72 (34·70, 34·75) | 11,944,365 | 2,480,500 | 20·83 (20·81, 20·86) | 12,299,145 | 3,446,610 | 28·10 (28·07, 28·12) |
| White Irish | 84,305 | 27,760 | 32·96 (32·64, 33·28) | 88,600 | 17,460 | 19·76 (19·50, 20·03) | 93,035 | 24,460 | 26·36 (26·07, 26·64) |
| Other White | 1,316,480 | 293,165 | 22·30 (22·23, 22·37) | 1,437,855 | 184,800 | 12·89 (12·83, 12·94) | 1,540,215 | 254,570 | 16·57 (16·51, 16·63) |
| Indian | 434,445 | 139,620 | 32·17 (32·03, 32·30) | 469,700 | 91,605 | 19·54 (19·43, 19·66) | 501,025 | 129,905 | 25·99 (25·87, 26·12) |
| Pakistani | 319,705 | 111,395 | 34·89 (34·72, 35·05) | 340,615 | 70,880 | 20·85 (20·72, 20·99) | 358,585 | 99,095 | 27·69 (27·55, 27·84) |
| Bangladeshi | 72,560 | 25,350 | 34·94 (34·59, 35·28) | 78,085 | 17,330 | 22·24 (21·95, 22·53) | 83,300 | 23,755 | 28·56 (28·26, 28·87) |
| Chinese | 93,050 | 15,860 | 17·08 (16·83, 17·32) | 106,645 | 9,570 | 9·00 (8·83, 9·17) | 115,825 | 12,910 | 11·17 (10·99, 11·35) |
| Other Asian | 223,795 | 65,660 | 29·35 (29·16, 29·54) | 245,040 | 44,760 | 18·29 (18·14, 18·44) | 264,080 | 62,390 | 23·67 (23·50, 23·83) |
| Black Caribbean | 86,680 | 32,595 | 37·62 (37·30, 37·95) | 91,260 | 21,190 | 23·24 (22·96, 23·51) | 95,140 | 29,520 | 31·09 (30·80, 31·39) |
| Black African | 183,060 | 49,330 | 26·97 (26·77, 27·17) | 202,735 | 33,185 | 16·40 (16·24, 16·56) | 219,880 | 46,435 | 21·16 (20·99, 21·33) |
| Other Black | 75,545 | 22,845 | 30·26 (29·94, 30·59) | 82,465 | 14,490 | 17·63 (17·37, 17·89) | 89,040 | 21,265 | 23·95 (23·67, 24·23) |
| White & Black Caribbean | 43,290 | 13,065 | 30·22 (29·78, 30·65) | 46,580 | 8,905 | 19·19 (18·83, 19·55) | 49,930 | 11,655 | 23·42 (23·04, 23·79) |
| White & Black African | 32,370 | 8,375 | 25·91 (25·43, 26·38) | 35,720 | 5,660 | 15·90 (15·52, 16·28) | 39,065 | 7,720 | 19·80 (19·41, 20·20) |
| White & Asian | 35,450 | 9,240 | 26·14 (25·68, 26·59) | 39,050 | 6,250 | 16·07 (15·70, 16·43) | 42,740 | 8,530 | 19·99 (19·62, 20·37) |
| Other Mixed | 69,245 | 17,055 | 24·68 (24·36, 25·01) | 76,315 | 11,705 | 15·39 (15·13, 15·64) | 82,940 | 15,515 | 18·76 (18·50, 19·03) |
| Other | 234,955 | 57,620 | 24·55 (24·38, 24·73) | 261,295 | 39,790 | 15·25 (15·12, 15·39) | 286,925 | 53,955 | 18·84 (18·70, 18·98) |
| Missing | 1,405,850 | 187,700 | 13·38 (13·33, 13·44) | 1,491,055 | 115,900 | 7·80 (7·76, 7·84) | 1,581,510 | 174,020 | 11·04 (10·99, 11·09) |
| Long Term Condition |  |  |  |  |  |  |  |  |  |
| Hypertension | 3,230,230 | 1,837,260 | 56·92 (56·86, 56·97) | 3,430,595 | 1,234,855 | 36·07 (36·02, 36·12) | 3,558,405 | 2,673,785 | 49·22 (49·17, 49·27) |
| Type 2 Diabetes | 1,051,605 | 867,735 | 82·54 (82·46, 82·61) | 1,151,500 | 669,295 | 58·21 (58·12, 58·30) | 1,231,455 | 887,870 | 72·18 (72·10, 72·26) |
| Type 1 Diabetes | 83,455 | 59,420 | 71·25 (70·94, 71·56) | 87,905 | 41,440 | 47·25 (46·92, 47·58) | 93,505 | 55,640 | 59·59 (59·28, 59·90) |
| Learning Disability | 93,420 | 61,110 | 65·65 (65·34, 65·95) | 99,295 | 51,200 | 51·87 (51·56, 52·18) | 105,855 | 65,265 | 62·03 (61·74, 62·32) |
| Depression | 3,197,730 | 1,233,900 | 38·66 (38·60, 38·71) | 3,415,085 | 806,460 | 23·70 (23·65, 23·74) | 3,592,080 | 1,140,545 | 31·85 (31·80, 31·89) |
| Dementia | 90,895 | 48,460 | 53·45 (53·13, 53·78) | 114,135 | 40,295 | 35·50 (35·22, 35·78) | 134,080 | 60,785 | 45·57 (45·31, 45·84) |
| Serious Mental Illness | 169,995 | 116,055 | 68·38 (68·16, 68·60) | 182,630 | 88,595 | 48·65 (48·42, 48·88) | 195,450 | 118,010 | 60·56 (60·34, 60·77) |
| COPD | 458,960 | 308,030 | 67·25 (67·12, 67·39) | 506,180 | 181,610 | 36·04 (35·91, 36·17) | 531,475 | 261,870 | 49·49 (49·36, 49·63) |
| Asthma | 2,660,330 | 1,040,870 | 39·20 (39·14, 39·25) | 2,801,825 | 634,885 | 22·74 (22·69, 22·79) | 2,930,645 | 871,240 | 29·82 (29·76, 29·87) |
| Cardiovascular Disease | 940,115 | 588,970 | 62·69 (62·59, 62·78) | 1,021,155 | 407,485 | 39·98 (39·89, 40·08) | 1,087,320 | 578,685 | 53·31 (53·22, 53·40) |
| Stroke and TIA | 387,020 | 226,455 | 58·56 (58·41, 58·72) | 428,365 | 157,285 | 36·81 (36·66, 36·95) | 465,645 | 229,915 | 49·49 (49·35, 49·64) |
| Cancer | 766,395 | 348,585 | 45·52 (45·41, 45·63) | 839,895 | 232,940 | 27·80 (27·70, 27·89) | 905,475 | 348,225 | 38·54 (38·44, 38·64) |

BMI: measured in weight in kilograms/height in meters squared (kg/m^2^). N: Total number of the population with data extracted. n: total number of the population with a BMI value recorded. % (95% CI): percentage (95% Confidence Interval) of the population with a BMI recorded in each year. IMD: Index of Multiple Deprivation. Serious Mental Illness includes bipolar disorder and psychosis. COPD: Chronic Obstructive Pulmonary Disease. TIA: Transient Ischaemic Attack.
