# Supplementary material for: Trends in weight gain recorded in English primary care before and during the Coronavirus-19 pandemic: An observational cohort study using the OpenSAFELY platform
Source: PLoS Med. 2024 Jun 24;21(6):e1004398. doi: 10.1371/journal.pmed.1004398 (PMC11249215; doi:10.1371/journal.pmed.1004398)
Supplement: S3 Table — (DOCX) [file pmed.1004398.s008.docx]

S3 Table: Rate of weight gain of adults living in England before and after the onset of the COVID-19 pandemic calculated from measures of weight recorded in the routine healthcare record.

|  | Rate of Weight Gain (kg/m^2^/year). | | | |
| --- | --- | --- | --- | --- |
|  | Pandemic | | Prepandemic | |
|  | N (%) | Mean (SD) | N (%) | Mean (SD) |
| Total Population | 3,214,155 | 0·06 (1·20) | 3,966,495 | 0·08 (1·05) |
| Sex |  |  |  |  |
| Female | 1,898,510 (59·1) | 0·13 (1·27) | 2,381,345 (60·0) | 0·12 (1·12) |
| Male | 1,315,645 (40·9) | -0·03 (1·08) | 1,585,155 (40·0) | 0·02 (0·94) |
| Age Group (years) |  |  |  |  |
| 18-29 | 267,605 (8·3) | 0·40 (1·49) | 274,540 (6·9) | 0·45 (1·36) |
| 30-39 | 341,870 (10·6) | 0·30 (1·40) | 466,285 (11·8) | 0·28 (1·23) |
| 40-49 | 415,805 (12·9) | 0·17 (1·27) | 545,760 (13·8) | 0·17 (1·12) |
| 50-59 | 603,670 (18·8) | 0·07 (1·19) | 757,830 (19·1) | 0·08 (1·05) |
| 60-69 | 642,120 (20·0) | -0·00 (1·09) | 771,890 (19·5) | -0·00 (0·96) |
| 70-79 | 635,175 (19·8) | -0·08 (1·01) | 757,405 (19·1) | -0·06 (0·87) |
| 80-90 | 307,910 (9·6) | -0·21 (1·04) | 392,785 (9·9) | -0·15 (0·84) |
| Ethnicity |  |  |  |  |
| White British | 2,631,235 (81·9) | 0·07 (1·21) | 3,275,985 (82·6) | 0·07 (1·06) |
| White Irish | 17,590 (0·5) | -0·01 (1·18) | 21,500 (0·5) | 0·02 (1·02) |
| Other White | 172,260 (5·4) | 0·10 (1·24) | 211,145 (5·3) | 0·12 (1·08) |
| Indian | 99,750 (3·1) | -0·03 (1·02) | 115,585 (2·9) | 0·03 (0·92) |
| Pakistani | 80,400 (2·5) | 0·05 (1·09) | 99,045 (2·5) | 0·10 (0·98) |
| Bangladeshi | 18,965 (0·6) | 0·01 (1·03) | 21,230 (0·5) | 0·10 (0·94) |
| Chinese | 8,245 (0·3) | -0·04 (0·91) | 10,185 (0·3) | 0·04 (0·80) |
| Other Asian | 46,590 (1·4) | 0·02 (1·04) | 51,865 (1·3) | 0·08 (0·94) |
| Black African | 32,875 (1·0) | 0·14 (1·21) | 37,745 (1·0) | 0·13 (1·07) |
| Black Caribbean | 23,050 (0·7) | 0·06 (1·19) | 27,170 (0·7) | 0·03 (1·03) |
| Other Black | 15,020 (0·5) | 0·12 (1·23) | 17,295 (0·4) | 0·10 (1·10) |
| White & Black Caribbean | 8,840 (0·3) | 0·16 (1·35) | 10,610 (0·3) | 0·15 (1·18) |
| White & Black African | 5,460 (0·2) | 0·15 (1·27) | 6,400 (0·2) | 0·15 (1·07) |
| White & Asian | 5,945 (0·2) | 0·08 (1·19) | 7,055 (0·2) | 0·13 (1·08) |
| Other Mixed | 10,795 (0·3) | 0·14 (1·27) | 12,525 (0·3) | 0·16 (1·13) |
| Other | 37,130 (1·2) | 0·06 (1·17) | 41,155 (1·0) | 0·10 (1·06) |
| Patient IMD Quintile |  |  |  |  |
| 1 (most deprived) | 686,100 (21·3) | 0·12 (1·29) | 836,480 (21·1) | 0·11 (1·14) |
| 5 (least deprived) | 549,495 (17·1) | 0·01 (1·11) | 692,065 (17·4) | 0·06 (0·97) |
| Long Term Condition |  |  |  |  |
| Hypertension | 1,436,070 (44·7) | -0·04 (1·11) | 1,637,165 (41·3) | -0·03 (0·96) |
| Type 1 Diabetes | 51,250 (1·6) | 0·18 (1·11) | 55,810 (1·4) | 0·13 (0·96) |
| Type 2 Diabetes | 793,770 (24·7) | -0·17 (1·12) | 820,390 (20·7) | -0·13 (0·99) |
| Cardiovascular Disease | 487,915 (15·2) | -0·08 (1·11) | 560,030 (14·1) | -0·05 (0·94) |
| Learning Difficulties | 55,750 (1·7) | 0·15 (1·41) | 54,580 (1·4) | 0·15 (1·23) |
| Depression | 887,240 (27·6) | 0·12 (1·34) | 1,105,540 (27·9) | 0·12 (1·17) |
| Dementia | 47,225 (1·5) | -0·23 (1·39) | 58,670 (1·5) | -0·15 (1·09) |
| Serious Mental Illness | 101,395 (3·2) | 0·13 (1·47) | 100,025 (2·5) | 0·11 (1·26) |
| Asthma | 709,730 (22·1) | 0·09 (1·29) | 939,925 (23·7) | 0·10 (1·11) |
| COPD | 227,935 (7·1) | -0·05 (1·21) | 299,095 (7·5) | 0·01 (1·01) |
| Stroke and TIA | 187,580 (5·8) | -0·09 (1·14) | 219,360 (5·5) | -0·06 (0·96) |

Rate of weight gain in kilograms/meter squared/year (kg/m^2^/year). N(%): total number (percentage) of each population subgroup that contributed data to the analysis. SD: Standard Deviation. IMD: Index of Multiple Deprivation. COPD: Chronic Obstructive Pulmonary Disease. TIA: Transient Ischaemic Attack
