# Supplementary material for: Trends in weight gain recorded in English primary care before and during the Coronavirus-19 pandemic: An observational cohort study using the OpenSAFELY platform
Source: PLoS Med. 2024 Jun 24;21(6):e1004398. doi: 10.1371/journal.pmed.1004398 (PMC11249215; doi:10.1371/journal.pmed.1004398)
Supplement: S4 Table — (DOCX) [file pmed.1004398.s009.docx]

S4 Table: Estimated associations between sociodemographic and clinical characteristics and odds of rapid weight gain during the prepandemic and pandemic period

|  | Prepandemic | | | | | Pandemic | | | | |
| --- | --- | --- | --- | --- | --- | --- | --- | --- | --- | --- |
|  | N (%) | Rapid Weight Gain | | | | N (%) | Rapid Weight Gain | | | |
|  |  | n | % | aOR (95% CI) | p |  | n | % | aOR (95% CI) | p |
| Total | 3,966,500 | 1,067,825 | 26.92 |  |  | 3,214,155 | 938,495 | 29.20 |  |  |
| Sex |  |  |  |  |  |  |  |  |  |  |
| Female | 2,381,345 (60.0) | 717,030 | 30.11 | 1 |  | 1,898,510 (59.1) | 624,095 | 32.87 | 1 |  |
| Male | 1,585,155 (40.0) | 350,795 | 22.13 | 0.80 (0.79,0.80) | <0.001 | 1,315,645 (40.9) | 314,400 | 23.90 | 0.76 (0.76,0.76) | <0.001 |
| Age Group (years) |  |  |  |  |  |  |  |  |  |  |
| 18-29 | 274,540 (6.9) | 126,290 | 46.00 | 1 |  | 267,605 (8.3) | 118,240 | 44.18 |  |  |
| 30-39 | 466,285 (11.8) | 180,445 | 38.70 | 0.76 (0.75,0.76) | <0.001 | 341,870 (10.6) | 139,405 | 40.78 | 0.90 (0.89,0.91) | <0.001 |
| 40-49 | 545,760 (13.8) | 176,160 | 32.28 | 0.60 (0.59,0.60) | <0.001 | 415,805 (12.9) | 143,805 | 34.58 | 0.73 (0.72,0.74) | <0.001 |
| 50-59 | 757,830 (19.1) | 209,740 | 27.68 | 0.49 (0.48,0.49) | <0.001 | 603,670 (18.8) | 181,610 | 30.08 | 0.60 (0.60,0.61) | <0.001 |
| 60-69 | 771,890 (19.5) | 175,080 | 22.68 | 0.38 (0.37,0.38) | <0.001 | 642,120 (20.0) | 164,025 | 25.54 | 0.49 (0.48,0.49) | <0.001 |
| 70-79 | 757,405 (19.1) | 139,565 | 18.43 | 0.29 (0.29,0.29) | <0.001 | 635,175 (19.8) | 135,185 | 21.28 | 0.38 (0.38,0.39) | <0.001 |
| 80-90 | 392,785 (9.9) | 60,545 | 15.41 | 0.23 (0.23,0.23) | <0.001 | 307,910 (9.6) | 56,225 | 18.26 | 0.31 (0.31,0.31) | <0.001 |
| Patient IMD Quintile |  |  |  |  |  |  |  |  |  |  |
| 1 (Most Deprived) | 836,480 (21.1) | 249,800 | 29.86 | 1 |  | 686,100 (21.3) | 223,755 | 32.61 | 1 |  |
| 5 (Least Deprived) | 692,065 (17.4) | 168,825 | 24.39 | 0.82 (0.82,0.83) | <0.001 | 549,495 (17.1) | 143,275 | 26.07 | 0.77 (0.77,0.78) | <0.001 |
| Ethnicity |  |  |  |  |  |  |  |  |  |  |
| White British | 3,275,985 (82.6) | 882,610 | 26.94 | 1 |  | 2,631,235 (81.9) | 774,230 | 29.42 | 1 |  |
| White Irish | 21,500 (0.5) | 5,190 | 24.14 | 0.97 (0.94,1.00) | 0.072 | 17,590 (0.5) | 4,655 | 26.46 | 0.94 (0.91,0.97) | 0.001 |
| Other White | 211,145 (5.3) | 61,595 | 29.17 | 0.98 (0.97,0.99) | <0.001 | 172,260 (5.4) | 53,390 | 30.99 | 0.94 (0.93,0.95) | <0.001 |
| Indian | 115,585 (2.9) | 26,155 | 22.63 | 0.74 (0.73,0.76) | <0.001 | 99,750 (3.1) | 23,060 | 23.12 | 0.68 (0.67,0.69) | <0.001 |
| Pakistani | 99,045 (2.5) | 26,785 | 27.04 | 0.79 (0.78,0.80) | <0.001 | 80,400 (2.5) | 22,160 | 27.56 | 0.72 (0.71,0.74) | <0.001 |
| Bangladeshi | 21,230 (0.5) | 5,340 | 25.15 | 0.70 (0.68,0.72) | <0.001 | 18,965 (0.6) | 4,675 | 24.65 | 0.60 (0.58,0.62) | <0.001 |
| Chinese | 10,185 (0.3) | 2,025 | 19.88 | 0.62 (0.59,0.66) | <0.001 | 8,245 (0.3) | 1,660 | 20.13 | 0.56 (0.53,0.59) | <0.001 |
| Other Asian | 51,865 (1.3) | 12,810 | 24.70 | 0.77 (0.75,0.78) | <0.001 | 46,590 (1.5) | 11,810 | 25.35 | 0.70 (0.69,0.72) | <0.001 |
| Black Caribbean | 37,745 (0.9) | 11,055 | 29.29 | 0.85 (0.83,0.87) | <0.001 | 32,875 (1.0) | 10,655 | 32.41 | 0.91 (0.89,0.94) | <0.001 |
| Black African | 27,170 (0.7) | 6,685 | 24.60 | 0.89 (0.87,0.91) | <0.001 | 23,050 (0.7) | 6,635 | 28.79 | 0.91 (0.89,0.93) | <0.001 |
| Other Black | 17,295 (0.4) | 4,920 | 28.45 | 0.89 (0.86,0.92) | <0.001 | 15,020 (0.5) | 4,790 | 31.89 | 0.92 (0.89,0.95) | <0.001 |
| White/Asian | 10,610 (0.3) | 3,405 | 32.09 | 0.87 (0.82,0.91) | <0.001 | 8,840 (0.3) | 3,100 | 35.07 | 0.78 (0.74,0.83) | <0.001 |
| White/Black Caribbean | 6,400 (0.2) | 1,970 | 30.78 | 0.94 (0.90,0.98) | 0.004 | 5,460 (0.2) | 1,810 | 33.15 | 0.97 (0.93,1.01) | 0.159 |
| White/Black African | 7,055 (0.2) | 2,040 | 28.92 | 0.95 (0.90,1.00) | 0.050 | 5,945 (0.2) | 1,735 | 29.18 | 0.94 (0.88,0.99) | 0.022 |
| Other Mixed | 12,525 (0.3) | 3,805 | 30.38 | 0.92 (0.89,0.96) | <0.001 | 10,795 (0.3) | 3,490 | 32.33 | 0.90 (0.87,0.94) | <0.001 |
| Other | 41,155 (1.0) | 11,430 | 27.77 | 0.88 (0.86,0.90) | <0.001 | 37,130 (1.2) | 10,640 | 28.66 | 0.82 (0.80,0.84) | <0.001 |
| Long Term Condition |  |  |  |  |  |  |  |  |  |  |
| Hypertension | 1,637,165 (41.3) | 357,645 | 21.85 | 1.04 (1.03,1.04) | <0.001 | 1,436,070 (44.7) | 355,240 | 24.74 | 1.03 (1.03,1.04) | <0.001 |
| Type 1 Diabetes | 55,810 (1.4) | 15,475 | 27.73 | 0.93 (0.91,0.94) | <0.001 | 51,250 (1.6) | 16,690 | 32.57 | 1.06 (1.04,1.08) | <0.001 |
| Type 2 Diabetes | 820,390 (20.7) | 157,920 | 19.25 | 0.79 (0.79,0.80) | <0.001 | 793,770 (24.7) | 164,565 | 20.73 | 0.71 (0.71,0.72) | <0.001 |
| Cardiovascular Disease | 560,030 (14.1) | 116,860 | 20.87 | 1.07 (1.06,1.08) | <0.001 | 487,915 (15.2) | 115,800 | 23.73 | 1.07 (1.06,1.08) | <0.001 |
| Stroke and TIA | 219,360 (5.5) | 45,775 | 20.87 | 1.06 (1.05,1.08) | <0.001 | 187,580 (5.8) | 44,485 | 23.72 | 1.05 (1.04,1.07) | <0.001 |
| Learning Difficulties | 54,580 (1.4) | 18,555 | 34.00 | 1.08 (1.07,1.10) | <0.001 | 55,750 (1.7) | 20,260 | 36.34 | 1.10 (1.08,1.12) | <0.001 |
| Depression | 1,105,540 (27.9) | 341,600 | 30.90 | 1.19 (1.18,1.19) | <0.001 | 887,240 (27.6) | 296,010 | 33.36 | 1.18 (1.17,1.18) | <0.001 |
| Serious Mental Illness | 100,025 (2.5) | 31,880 | 31.87 | 1.20 (1.18,1.22) | <0.001 | 101,395 (3.2) | 35,655 | 35.16 | 1.24 (1.22,1.27) | <0.001 |
| Dementia | 58,670 (1.5) | 12,180 | 20.76 | 1.20 (1.18,1.23) | <0.001 | 47,225 (1.5) | 11,660 | 24.69 | 1.23 (1.21,1.24) | <0.001 |
| Asthma | 939,925 (23.7) | 271,460 | 28.88 | 1.03 (1.03,1.04) | <0.001 | 709,730 (22.1) | 222,290 | 31.32 | 1.03 (1.03,1.04) | <0.001 |
| COPD | 299,095 (7.5) | 73,105 | 24.44 | 1.22 (1.21,1.23) | <0.001 | 227,935 (7.1) | 59,700 | 26.19 | 1.12 (1.10,1.13) | <0.001 |

Rapid weight gain is defined as a rate of weight gain ≥ 0.5 kg/m^2^/year. N(%): the total number (and percentage) of individuals within population subgroups. n: number within each population subgroup that experienced rapid weight gain. %: percentage within each subgroup that experienced rapid weight gain. aOR: adjusted Odds Ratio adjusted for age, sex, ethnicity, and Index of Multiple Deprivation (IMD). aOR for long term conditions presented in comparison to a reference group without the condition. CI: confidence interval, IMD: Index of Multiple Deprivation, COPD: Chronic Obstructive Pulmonary Disease. TIA: Transient Ischaemic Attack.
