# Supplementary material for: Trends in weight gain recorded in English primary care before and during the Coronavirus-19 pandemic: An observational cohort study using the OpenSAFELY platform
Source: PLoS Med. 2024 Jun 24;21(6):e1004398. doi: 10.1371/journal.pmed.1004398 (PMC11249215; doi:10.1371/journal.pmed.1004398)
Supplement: S5 Table — (DOCX) [file pmed.1004398.s010.docx]

S5 Table. Estimated associations between sociodemographic and clinical characteristics and odds of extreme acceleration in rate of weight gain between the prepandemic and pandemic period.

|  | N (%) | Extreme Acceleration (%) | | | |
| --- | --- | --- | --- | --- | --- |
|  |  | n | % | aOR (95% CI) | p |
| Sex |  |  |  |  |  |
| Female | 1,612,850 (58.2) | 186,835 | 11.58 | 1 |  |
| Male | 1,155,845 (41.8) | 90,030 | 7.79 | 0.72 (0.72, 0.73) | <0.001 |
| Age Group (years) |  |  |  |  |  |
| 18-29 | 161,655 (5.8) | 24,025 | 14.86 | 1 |  |
| 30-39 | 253,415 (9.2) | 36,640 | 14.46 | 1.00 (0.98, 1.02) | 0.916 |
| 40-49 | 310,045 (11.2) | 36,735 | 11.85 | 0.85 (0.84, 0.87) | <0.001 |
| 50-59 | 479,655 (17.3) | 51,775 | 10.79 | 0.79 (0.78, 0.80) | <0.001 |
| 60-69 | 573,030 (20.7) | 53,190 | 9.28 | 0.68 (0.67, 0.69) | <0.001 |
| 70-79 | 649,920 (23.5) | 49,900 | 7.68 | 0.55 (0.54, 0.56) | <0.001 |
| 80-90 | 340,975 (12.3) | 24,600 | 7.21 | 0.51 (0.50, 0.52) | <0.001 |
| Patient IMD Quintile |  |  |  |  |  |
| 1 (most deprived) | 592,805 (21.4) | 69,165 | 11.67 | 1 | <0.001 |
| 5 (least deprived) | 472,800 (17.1) | 39,990 | 8.46 | 0.72 (0.71, 0.73) | <0.001 |
| Ethnicity |  |  |  |  |  |
| White British | 2,306,865 (83.3) | 235,375 | 10.20 | 1 |  |
| White Irish | 15,475 (0.6) | 1,455 | 9.40 | 0.98 (0.93, 1.03) | 0.425 |
| Other White | 131,960 (4.8) | 13,495 | 10.23 | 0.92 (0.90, 0.94) | <0.001 |
| Indian | 82,245 (3.0) | 5,795 | 7.05 | 0.63 (0.62, 0.65) | <0.001 |
| Pakistani | 68,000 (2.5) | 5,665 | 8.33 | 0.65 (0.63, 0.67) | <0.001 |
| Bangladeshi | 15,025 (0.5) | 1,085 | 7.22 | 0.55 (0.51, 0.58) | <0.001 |
| Chinese | 6,395 (0.2) | 335 | 5.24 | 0.47 (0.42, 0.52) | <0.001 |
| Other Asian | 36,420 (1.3) | 2,675 | 7.34 | 0.62 (0.60, 0.65) | <0.001 |
| Black Caribbean | 20,330 (0.7) | 2,090 | 10.28 | 0.93 (0.89, 0.97) | 0.002 |
| Black African | 23,970 (0.9) | 2,500 | 10.43 | 0.84 (0.81, 0.88) | <0.001 |
| Other Black | 11,300 (0.4) | 1,260 | 11.15 | 0.93 (0.87, 0.98) | 0.012 |
| Mixed White/Black Caribbean | 6,960 (0.2) | 870 | 12.50 | 1.00 (0.93, 1.07) | 0.977 |
| Mixed White/Black African | 3,935 (0.1) | 440 | 11.18 | 0.92 (0.84, 1.02) | 0.121 |
| Mixed White/Asian | 4,445 (0.2) | 405 | 9.11 | 0.75 (0.68, 0.83) | <0.001 |
| Other Mixed | 7,950 (0.3) | 855 | 10.75 | 0.90 (0.83, 0.96) | 0.002 |
| Other | 27,420 (1.0) | 2,565 | 9.35 | 0.81 (0.77, 0.84) | <0.001 |
| Long Term Condition |  |  |  |  |  |
| Hypertension | 1,399,300 (50.5) | 124,630 | 8.91 | 1.07 (1.06, 1.08) | <0.001 |
| Type 1 Diabetes | 47,910 (1.7) | 4,495 | 9.38 | 0.87 (0.85, 0.90) | <0.001 |
| Type 2 Diabetes | 805,315 (29.1) | 72,995 | 9.06 | 1.07 (1.06, 1.08) | <0.001 |
| Cardiovascular Disease | 498,305 (18.0) | 43,165 | 8.66 | 1.10 (1.08, 1.11) | <0.001 |
| Stroke and TIA | 190,500 (6.9) | 17,240 | 9.05 | 1.12 (1.10, 1.14) | <0.001 |
| Learning Difficulties | 49,865 (1.8) | 6,925 | 13.89 | 1.25 (1.21, 1.28) | <0.001 |
| Depression | 777,355 (28.1) | 97,095 | 12.49 | 1.28 (1.27, 1.29) | <0.001 |
| Serious Mental Illness | 88,375 (3.2) | 12,960 | 14.66 | 1.47 (1.44, 1.50) | <0.001 |
| Dementia | 48,415 (1.8) | 5,940 | 12.27 | 1.68 (1.64, 1.73) | <0.001 |
| Asthma | 638,485 (23.1) | 71,680 | 11.23 | 1.09 (1.08, 1.10) | <0.001 |
| COPD | 245,510 (8.9) | 24,165 | 9.84 | 1.14 (1.12, 1.16) | <0.001 |

Extreme acceleration in rate of weight gain is defined as δ-change ≥ 1·84 kilograms (kg)/metre squared(m^2^)/year. δ-change refers to the change (δ) in rate of weight gain between the prepandemic (δ-prepandemic) and pandemic (δ-pandemic) periods: δ-change = δ-pandemic - δ-prepandemic. N (%): Number (and percentage) of individuals within population subgroups. n: number within each population subgroup that experienced extreme acceleration in rate of weight gain. %: percentage of each group that experienced extreme acceleration. aOR: adjusted Odds Ratio of extreme acceleration in rate of weight gain adjusted for age, sex, ethnicity, and Index of Multiple Deprivation. aOR for long term conditions presented in comparison to a reference group without the condition. CI: confidence interval, IMD: Index of Multiple Deprivation, COPD: Chronic Obstructive Pulmonary Disease. TIA: Transient Ischaemic Attack.
