# Supplementary material for: Trends in weight gain recorded in English primary care before and during the Coronavirus-19 pandemic: An observational cohort study using the OpenSAFELY platform
Source: PLoS Med. 2024 Jun 24;21(6):e1004398. doi: 10.1371/journal.pmed.1004398 (PMC11249215; doi:10.1371/journal.pmed.1004398)
Supplement: S6 Table — (DOCX) [file pmed.1004398.s011.docx]

S6 Table: Associations between sociodemographic and clinical characteristics and odds of extreme acceleration in rate of weight gain during the pandemic in analyses stratified by age group.

|  |  | Age Group: 18 – 39 Years | | | |  | Age Group: 40 - 59 Years | | | |  | Age Group: 60 - 79 Years | | | |
| --- | --- | --- | --- | --- | --- | --- | --- | --- | --- | --- | --- | --- | --- | --- | --- |
|  |  | Extreme Acceleration | | | |  |  | Extreme Acceleration | | |  |  | Extreme Acceleration | | |
|  | N (%) |  | % | aOR (95% CI) | p | N (%) | n | % | aOR (95% CI) | p | n | N (%) | % | aOR (95% CI) | p |
| Sex |  |  |  |  |  |  |  |  |  |  |  |  |  |  |  |
| Female | 361,125 (87.00) | 53,335 | 14.77 | 1 |  | 478,495 (60.59) | 58,775 | 12.28 | 1 |  | 59,140 | 589,805 (48.23) | 10.03 | 1 |  |
| Male | 53,950 (13.00) | 7,335 | 13.60 | 0.91 (0.89,0.94) | <0.001 | 311,205 (39.41) | 29,735 | 9.55 | 0.76 (0.75,0.77) | <0.001 | 43,950 | 633,150 (51.77) | 6.94 | 0.67 (0.66,0.68) | <0.001 |
| Ethnicity |  |  |  |  |  |  |  |  |  |  |  |  |  |  |  |
| White | 365,300 (88.01) |  | 14.84 | 1 |  | 650,425 (82.36) | 76,820 | 11.81 | 1 |  |  | 1,116,720 (91.31) | 8.60 | 1 |  |
| Black | 7,530 (1.81) | 54,220 | 15.47 | 0.99 (0.93,1.05) | 0.686 | 26,930 (3.41) | 2,890 | 10.73 | 0.84 (0.81,0.88) | <0.001 | 96,070 | 16,785 (1.37) | 8.58 | 0.90 (0.85,0.95) | <0.001 |
| South Asian | 25,240 (6.08) | 1,165 | 12.08 | 0.74 (0.72,0.77) | <0.001 | 71,565 (9.06) | 5,300 | 7.41 | 0.57 (0.55,0.59) | <0.001 | 1,440 | 58,635 (4.79) | 5.98 | 0.63 (0.61,0.65) | <0.001 |
| Mixed | 6,450 (1.55) | 3,050 | 14.42 | 0.94 (0.87,1.00) | 0.065 | 9,520 (1.21) | 1,020 | 10.71 | 0.87 (0.81,0.93) | <0.001 | 3,505 | 6,220 (0.51) | 8.52 | 0.94 (0.86,1.03) | 0.178 |
| Chinese/Other | 10,555 (2.54) | 930 | 12.41 | 0.79 (0.74,0.83) | <0.001 | 31,245 (3.96) | 2,470 | 7.91 | 0.63 (0.60,0.65) | <0.001 | 530 | 24,595 (2.01) | 6.32 | 0.69 (0.65,0.72) | <0.001 |
| Patient IMD Quintile |  |  |  |  |  |  |  |  |  |  |  |  |  |  |  |
| 1 (most deprived) | 107,900 (26.00) | 17,760 | 16.46 | 1 |  | 200,040 (25.33) | 24,705 | 12.35 | 1 |  | 22,410 | 233,525 (19.10) | 9.60 | 1 |  |
| 5 (least deprived) | 59,650 (14.37) | 7,430 | 12.46 | 0.70 (0.68,0.72) | <0.001 | 117,490 (14.88) | 11,210 | 9.54 | 0.69 (0.67,0.71) | <0.001 | 16,595 | 222,965 (18.23) | 7.44 | 0.74 (0.73,0.76) | <0.001 |
| Long Term Condition |  |  |  |  |  |  |  |  |  |  |  |  |  |  |  |
| Hypertension |  |  |  |  |  |  |  |  |  |  |  |  |  |  |  |
| Absent | 394,430 (95.03) | 57,520 | 14.58 | 1 |  | 504,485 (63.88) | 56,340 | 11.17 | 1 |  | 33,450 | 400,210 (32.73) | 8.36 | 1 |  |
| Present | 20,640 (4.97) | 3,150 | 15.26 | 1.07 (1.03,1.12) | <0.001 | 285,215 (36.12) | 32,170 | 11.28 | 1.06 (1.04,1.08) | <0.001 | 69,640 | 822,740 (67.27) | 8.46 | 1.03 (1.01,1.04) | <0.001 |
| Type 1 Diabetes |  |  |  |  |  |  |  |  |  |  |  |  |  |  |  |
| Absent | 403,630 (97.24) | 59,210 | 14.67 | 1 |  | 769,935 (97.50) | 86,655 | 11.25 | 1 |  | 102,055 | 1,208,365 (98.81) | 8.45 | 1 |  |
| Present | 11,445 (2.76) | 1,460 | 12.76 | 0.88 (0.83,0.93) | <0.001 | 19,765 (2.50) | 1,860 | 9.41 | 0.84 (0.80,0.88) | <0.001 | 1,035 | 14,585 (1.19) | 7.10 | 0.84 (0.79,0.89) | <0.001 |
| Type 2 Diabetes |  |  |  |  |  |  |  |  |  |  |  |  |  |  |  |
| Absent | 397,320 (95.72) | 58,240 | 14.66 | 1 |  | 584,335 (74.00) | 66,700 | 11.41 | 1 |  | 63,700 | 766,865 (62.71) | 8.31 | 1 |  |
| Present | 17,755 (4.28) | 2,430 | 13.69 | 0.95 (0.91,1.00) | 0.042 | 205,360 (26.00) | 21,810 | 10.62 | 1.02 (1.00,1.03) | 0.066 | 39,390 | 456,085 (37.29) | 8.64 | 1.11 (1.09,1.12) | <0.001 |
| Cardiovascular Disease | |  |  |  |  |  |  |  |  |  |  |  |  |  |  |
| Absent | 409,100 (98.56) | 59,765 | 14.61 | 1 |  | 725,700 (91.90) | 80,990 | 11.16 | 1 |  | 78,190 | 925,885 (75.71) | 8.44 | 1 |  |
| Present | 5,970 (1.44) | 900 | 15.08 | 1.05 (0.98,1.13) | 0.166 | 63,995 (8.10) | 7,520 | 11.75 | 1.12 (1.09,1.15) | <0.001 | 24,900 | 297,065 (24.29) | 8.38 | 1.06 (1.04,1.07) | <0.001 |
| Learning Disability |  |  |  |  |  |  |  |  |  |  |  |  |  |  |  |
| Absent | 395,630 (95.32) | 57,845 | 14.62 | 1 |  | 769,390 (97.43) | 85,825 | 11.15 | 1 |  | 101,750 | 1,213,315 (99.21) | 8.39 | 1 |  |
| Present | 19,445 (4.68) | 2,825 | 14.53 | 1.02 (0.98,1.07) | 0.304 | 20,310 (2.57) | 2,685 | 13.22 | 1.19 (1.14,1.24) | <0.001 | 1,340 | 9,635 (0.79) | 13.91 | 1.71 (1.61,1.81) | <0.001 |
| Depression |  |  |  |  |  |  |  |  |  |  |  |  |  |  |  |
| Absent | 292,005 (70.35) | 39,000 | 13.36 | 1 |  | 511,055 (64.72) | 50,750 | 9.93 | 1 |  | 70,585 | 905,980 (74.08) | 7.79 | 1 |  |
| Present | 123,070 (29.65) | 21,670 | 17.61 | 1.34 (1.32,1.37) | <0.001 | 278,645 (35.28) | 37,760 | 13.55 | 1.31 (1.29,1.33) | <0.001 | 32,505 | 316,970 (25.92) | 10.25 | 1.24 (1.23,1.26) | <0.001 |
| Dementia |  |  |  |  |  |  |  |  |  |  |  |  |  |  |  |
| Absent | 414,955 (99.97) | 60,650 | 14.62 | 1 |  | 787,345 (99.70) | 88,210 | 11.20 | 1 |  | 100,490 | 1,201,495 (98.25) | 8.36 | 1 |  |
| Present | 120 (0.03) | 20 | 16.67 | 1.11 (0.66,1.77) | 0.671 | 2,350 (0.30) | 300 | 12.77 | 1.15 (1.02,1.30) | 0.021 | 2,600 | 21,460 (1.75) | 12.12 | 1.51 (1.44,1.57) | <0.001 |
| Serious Mental Illness | |  |  |  |  |  |  |  |  |  |  |  |  |  |  |
| Absent | 400,270 (96.43) | 57,935 | 14.47 | 1 |  | 749,590 (94.92) | 82,545 | 11.01 | 1 |  | 99,315 | 1,193,430 (97.59) | 8.32 | 1 |  |
| Present | 14,805 (3.57) | 2,735 | 18.47 | 1.38 (1.32,1.44) | <0.001 | 40,105 (5.08) | 5,965 | 14.87 | 1.41 (1.37,1.45) | <0.001 | 3,775 | 29,520 (2.41) | 12.79 | 1.54 (1.49,1.60) | <0.001 |
| Asthma |  |  |  |  |  |  |  |  |  |  |  |  |  |  |  |
| Absent | 296,605 (71.46) | 42,150 | 14.21 | 1 |  | 587,935 (74.45) | 64,035 | 10.89 | 1 |  | 79,375 | 967,685 (79.13) | 8.20 | 1 |  |
| Present | 118,465 (28.54) | 18,515 | 15.63 | 1.11 (1.09,1.13) | <0.001 | 201,760 (25.55) | 24,475 | 12.13 | 1.08 (1.07,1.10) | <0.001 | 23,715 | 255,270 (20.87) | 9.29 | 1.09 (1.07,1.11) | <0.001 |
| COPD |  |  |  |  |  |  |  |  |  |  |  |  |  |  |  |
| Absent | 413,490 (99.62) | 60,440 | 14.62 | 1 |  | 753,610 (95.43) | 83,625 | 11.10 | 1 |  | 87,950 | 1,063,920 (87.00) | 8.27 | 1 |  |
| Present | 1,585 (0.38) | 230 | 14.51 | 0.96 (0.83,1.10) | 0.571 | 36,085 (4.57) | 4,885 | 13.54 | 1.16 (1.13,1.20) | <0.001 | 15,140 | 159,030 (13.00) | 9.52 | 1.11 (1.09,1.13) | <0.001 |
| Stroke and TIA |  |  |  |  |  |  |  |  |  |  |  |  |  |  |  |
| Absent | 413,265 (99.56) | 60,375 | 14.61 | 1 |  | 767,285 (97.16) | 85,720 | 11.17 | 1 |  | 93,440 | 1,115,200 (91.19) | 8.38 | 1 |  |
| Present | 1,810 (0.44) | 290 | 16.02 | 1.12 (0.99,1.27) | 0.069 | 22,415 (2.84) | 2,790 | 12.45 | 1.13 (1.08,1.17) | <0.001 | 9,650 | 107,755 (8.81) | 8.96 | 1.09 (1.07,1.12) | <0.001 |

Extreme acceleration in rate of weight gain is defined as δ-change ≥ 1.84 kilograms (kg)/metre squared (m^2^)/year. δ-change refers to the change (δ) in rate of weight gain between the prepandemic (δ-prepandemic) and pandemic (δ-pandemic) periods: δ-change = δ-pandemic - δ-prepandemic. N (%): Number (and percentage) of individuals within population subgroups. n: number within each population subgroup that experienced extreme acceleration in rate of weight gain. %: percentage of each group that experienced extreme acceleration. aOR: adjusted Odds Ratio of extreme acceleration in rate of weight gain adjusted for sex, ethnicity and IMD. aOR for long term conditions presented in comparison to a reference group without the condition. CI: confidence interval, IMD: Index of Multiple Deprivation, COPD: Chronic Obstructive Pulmonary Disease. TIA: Transient Ischaemic Attack.
