# Supplementary material for: Trends in weight gain recorded in English primary care before and during the Coronavirus-19 pandemic: An observational cohort study using the OpenSAFELY platform
Source: PLoS Med. 2024 Jun 24;21(6):e1004398. doi: 10.1371/journal.pmed.1004398 (PMC11249215; doi:10.1371/journal.pmed.1004398)
Supplement: S7 Table — (DOCX) [file pmed.1004398.s012.docx]

S7 Table. Associations between sociodemographic and clinical characteristics and odds of extreme acceleration in rate of weight gain during the pandemic in analyses stratified by sex.

|  | Female | | | | | Male | | | | |
| --- | --- | --- | --- | --- | --- | --- | --- | --- | --- | --- |
|  | N (%) | Extreme Acceleration | | | | N (%) | Extreme Acceleration | | | |
|  |  | n | % | aOR | p |  | n | % | aOR | p |
| Age Group (in years) |  |  |  |  |  |  |  |  |  |  |
| 18-29 | 145,605 (9.0) | 21,640 | 14.86 | 1 |  | 16,050 (1.4) | 2,390 | 14.89 | 1 |  |
| 30-39 | 215,520 (13.4) | 31,695 | 14.71 | 1.00 (0.98,1.02) | 0.982 | 37,895 (3.3) | 4,945 | 13.05 | 0.87 (0.83,0.92) | <0.001 |
| 40-49 | 214,595 (13.3) | 26,845 | 12.51 | 0.85 (0.83,0.86) | <0.001 | 95,450 (8.3) | 9,890 | 10.36 | 0.69 (0.66,0.72) | <0.001 |
| 50-59 | 263,900 (16.4) | 31,930 | 12.10 | 0.80 (0.79,0.82) | <0.001 | 215,755 (18.7) | 19,845 | 9.20 | 0.58 (0.56,0.61) | <0.001 |
| 60-69 | 273,820 (17.0) | 30,125 | 11.00 | 0.72 (0.71,0.74) | <0.001 | 299,215 (25.9) | 23,065 | 7.71 | 0.48 (0.46,0.50) | <0.001 |
| 70-79 | 315,985 (19.6) | 29,015 | 9.18 | 0.59 (0.58,0.60) | <0.001 | 333,935 (28.9) | 20,885 | 6.25 | 0.38 (0.36,0.40) | <0.001 |
| 80-90 | 183,430 (11.4) | 15,590 | 8.50 | 0.54 (0.53,0.56) | <0.001 | 157,545 (13.6) | 9,010 | 5.72 | 0.35 (0.33,0.37) | <0.001 |
| Ethnicity |  |  |  |  |  |  |  |  |  |  |
| White | 1,432,010 (88.8) | 169,280 | 11.82 | 1 |  | 1,022,295 (88.4) | 81,050 | 7.93 | 1 |  |
| Black | 33,525 (2.1) | 4,060 | 12.11 | 0.92 (0.89,0.95) | <0.001 | 22,080 (1.9) | 1,790 | 8.11 | 0.83 (0.79,0.87) | <0.001 |
| South Asian | 92,185 (5.7) | 8,085 | 8.77 | 0.64 (0.63,0.66) | <0.001 | 73,080 (6.3) | 4,455 | 6.10 | 0.60 (0.58,0.62) | <0.001 |
| Mixed | 15,240 (0.9) | 1,865 | 12.24 | 0.91 (0.86,0.95) | <0.001 | 8,050 (0.7) | 710 | 6.69 | 0.90 (0.83,0.97) | 0.009 |
| Chinese/Other | 39,890 (2.5) | 3,545 | 8.89 | 0.67 (0.65,0.70) | <0.001 | 30,345 (2.6) | 2,030 | 8.82 | 0.69 (0.65,0.72) | <0.001 |
| Patient IMD Quintile |  |  |  |  |  |  |  |  |  |  |
| 1 (most deprived) | 355,210 (22.0) | 47,510 | 13.38 | 1 |  | 237,595 (20.6) | 21,655 | 9.11 | 1 |  |
| 5 (least deprived) | 270,215 (16.8) | 26,570 | 9.83 | 0.71 (0.70,0.73) | <0.001 | 202,585 (17.5) | 13,420 | 6.62 | 0.75 (0.73,0.77) | <0.001 |
| Long Term Condition |  |  |  |  |  |  |  |  |  |  |
| Hypertension |  |  |  |  |  |  |  |  |  |  |
| Absent | 925,485 (57.4) | 114,805 | 12.40 | 1 |  | 443,910 (38.4) | 37,435 | 8.43 | 1 |  |
| Present | 687,365 (42.6) | 72,035 | 10.48 | 1.08 (1.07,1.10) | <0.001 | 711,935 (61.6) | 52,595 | 7.39 | 1.03 (1.02,1.05) | <0.001 |
| Type 1 Diabetes |  |  |  |  |  |  |  |  |  |  |
| Absent | 1,591,355 (98.7) | 184,340 | 11.58 | 1 |  | 1,129,435 (97.7) | 88,030 | 7.79 | 1 |  |
| Present | 21,495 (1.3) | 2,495 | 11.61 | 0.94 (0.91,0.98) | 0.008 | 26,410 (2.3) | 2,000 | 7.57 | 0.74 (0.71,0.78) | <0.001 |
| Type 2 Diabetes |  |  |  |  |  |  |  |  |  |  |
| Absent | 1,257,425 (78.0) | 148,190 | 11.79 | 1 |  | 705,950 (61.1) | 55,685 | 7.89 | 1 |  |
| Present | 355,425 (22.0) | 38,650 | 10.87 | 1.08 (1.07,1.10) | <0.001 | 449,895 (38.9) | 34,345 | 7.63 | 1.06 (1.04,1.07) | <0.001 |
| Cardiovascular Disease |  |  |  |  |  |  |  |  |  |  |
| Absent | 1,424,260 (88.3) | 166,500 | 11.69 | 1 |  | 846,130 (73.2) | 67,200 | 7.94 | 1 |  |
| Present | 188,590 (11.7) | 20,335 | 10.78 | 1.13 (1.11,1.14) | <0.001 | 309,715 (26.8) | 22,830 | 7.37 | 1.09 (1.07,1.11) | <0.001 |
| Learning Disability |  |  |  |  |  |  |  |  |  |  |
| Absent | 1,590,870 (98.6) | 183,400 | 11.53 | 1 |  | 1,127,955 (97.6) | 86,545 | 7.67 | 1 |  |
| Present | 21,980 (1.4) | 3,440 | 15.65 | 1.22 (1.18,1.27) | <0.001 | 27,890 (2.4) | 3,490 | 12.51 | 1.15 (1.10,1.19) | <0.001 |
| Depression |  |  |  |  |  |  |  |  |  |  |
| Absent | 1,081,410 (67.0) | 114,010 | 10.54 | 1 |  | 909,935 (78.7) | 65,760 | 7.23 | 1 |  |
| Present | 531,440 (33.0) | 72,825 | 13.70 | 1.28 (1.27,1.30) | <0.001 | 245,910 (21.3) | 24,270 | 9.87 | 1.26 (1.24,1.28) | <0.001 |
| Dementia |  |  |  |  |  |  |  |  |  |  |
| Absent | 1,586,340 (98.4) | 182,955 | 11.53 | 1 |  | 1,133,945 (98.1) | 87,975 | 7.76 | 1 |  |
| Present | 26,510 (1.6) | 3,885 | 14.65 | 1.76 (1.70,1.83) | <0.001 | 21,900 (1.9) | 2,055 | 9.38 | 1.55 (1.48,1.62) | <0.001 |
| Serious Mental Illness |  |  |  |  |  |  |  |  |  |  |
| Absent | 1,565,370 (97.1) | 179,195 | 11.45 | 1 |  | 1,114,950 (96.5) | 84,715 | 7.60 | 1 |  |
| Present | 47,480 (2.9) | 7,645 | 16.10 | 1.44 (1.40,1.48) | <0.001 | 40,895 (3.5) | 5,315 | 13.00 | 1.45 (1.41,1.49) | <0.001 |
| Asthma |  |  |  |  |  |  |  |  |  |  |
| Absent | 1,201,175 (74.5) | 134,335 | 11.18 | 1 |  | 929,035 (80.4) | 70,855 | 7.63 | 1 |  |
| Present | 411,675 (25.5) | 52,505 | 12.75 | 1.11 (1.10,1.13) | <0.001 | 226,810 (19.6) | 19,180 | 8.46 | 1.03 (1.01,1.05) | <0.001 |
| COPD |  |  |  |  |  |  |  |  |  |  |
| Absent | 1,494,310 (92.7) | 172,995 | 11.58 | 1 |  | 1,028,875 (89.0) | 79,710 | 7.75 | 1 |  |
| Present | 118,540 (7.3) | 13,840 | 11.68 | 1.13 (1.11,1.15) | <0.001 | 126,970 (11.0) | 10,320 | 8.13 | 1.16 (1.13,1.18) | <0.001 |
| Stroke and TIA |  |  |  |  |  |  |  |  |  |  |
| Absent | 1,526,200 (94.6) | 177,410 | 11.62 | 1 |  | 1,051,995 (91.0) | 82,215 | 7.82 | 1 |  |
| Present | 86,650 (5.4) | 9,425 | 10.88 | 1.13 (1.10,1.16) | <0.001 | 103,850 (9.0) | 7,815 | 7.53 | 1.12 (1.09,1.14) | <0.001 |

Extreme acceleration in rate of weight gain is defined as δ-change ≥ 1.84 kilograms (kg)/meter squared(m^2^)/year. δ-change refers to the change (δ) in rate of weight gain between the prepandemic (δ-prepandemic) and pandemic (δ-pandemic) periods: δ-change = δ-pandemic - δ-prepandemic. N (%): Number (and percentage) of individuals within population subgroups. n: number within each population subgroup that experienced extreme acceleration in rate of weight gain. %: percentage of each group that experienced extreme acceleration. aOR: adjusted Odds Ratio of extreme acceleration in rate of weight gain adjusted for age, IMD and ethnicity. aOR for long term conditions presented in comparison to a reference group without the condition. CI: confidence interval, IMD: Index of Multiple Deprivation, COPD: Chronic Obstructive Pulmonary Disease. TIA: Transient Ischaemic Attack.
