# Supplementary material for: Trends in weight gain recorded in English primary care before and during the Coronavirus-19 pandemic: An observational cohort study using the OpenSAFELY platform
Source: PLoS Med. 2024 Jun 24;21(6):e1004398. doi: 10.1371/journal.pmed.1004398 (PMC11249215; doi:10.1371/journal.pmed.1004398)
Supplement: S8 Table — (DOCX) [file pmed.1004398.s013.docx]

S8 Table. Associations between sociodemographic and clinical characteristics and odds of extreme acceleration in rate of weight gain during the pandemic in analyses stratified by ethnicity

|  | South Asian | | | | | Black | | | | |
| --- | --- | --- | --- | --- | --- | --- | --- | --- | --- | --- |
|  | N (%) | Extreme Acceleration | | | | N (%) | Extreme Acceleration | | | |
|  |  | n | % | aOR (95% CI) | p |  | n | % | aOR (95% CI) | p |
| Sex |  |  |  |  |  |  |  |  |  |  |
| Female | 92,185 (55.8) | 8,085 | 8.8 | 1 |  | 33,525 (60.3) | 4,060 | 12.1 | 1 |  |
| Male | 73,080 (44.2) | 4,455 | 6.1 | 0.73 (0.71,0.76) | <0.001 | 22,080 (39.7) | 1,790 | 8.1 | 0.69 (0.65,0.73) | <0.001 |
| Age Group (years) |  |  |  |  |  |  |  |  |  |  |
| 18-29 | 6,055 (3.7) | 830 | 13.7 | 1 |  | 2,040 (3.7) | 345 | 16.9 | 1 |  |
| 30-39 | 19,185 (11.6) | 2,220 | 11.6 | 0.83 (0.76,0.90) | <0.001 | 5,495 (9.9) | 820 | 14.9 | 0.86 (0.75,0.99) | 0.030 |
| 40-49 | 35,395 (21.4) | 2,875 | 8.1 | 0.59 (0.54,0.64) | <0.001 | 10,625 (19.1) | 1,260 | 11.9 | 0.69 (0.61,0.79) | <0.001 |
| 50-59 | 36,170 (21.9) | 2,425 | 6.7 | 0.49 (0.45,0.53) | <0.001 | 16,305 (29.3) | 1,630 | 10.0 | 0.59 (0.52,0.67) | <0.001 |
| 60-69 | 37,785 (22.9) | 2,270 | 6.0 | 0.44 (0.40,0.47) | <0.001 | 11,060 (19.9) | 960 | 8.7 | 0.51 (0.45,0.58) | <0.001 |
| 70-79 | 20,850 (12.6) | 1,230 | 5.9 | 0.42 (0.39,0.47) | <0.001 | 5,725 (10.3) | 475 | 8.3 | 0.48 (0.41,0.55) | <0.001 |
| 80-90 | 9,830 (5.9) | 685 | 7.0 | 0.51 (0.46,0.57) | <0.001 | 4,355 (7.8) | 355 | 8.2 | 0.48 (0.41,0.56) | <0.001 |
| Patient IMD Quintile |  |  |  |  |  |  |  |  |  |  |
| 1(most deprived) | 62,560 (37.9) | 5,100 | 8.2 | 1 |  | 23,530 (42.3) | 2,580 | 11.0 | 1 |  |
| 5(least deprived) | 10,545 (6.4) | 715 | 6.8 | 0.89 (0.82,0.96) | 0.005 | 2,910 (5.2) | 320 | 11.0 | 1.04 (0.92,1.18) | 0.496 |
| Long Term Condition |  |  |  |  |  |  |  |  |  |  |
| Hypertension |  |  |  |  |  |  |  |  |  |  |
| Absent | 89,415 (54.1) | 7,520 | 8.4 | 1 |  | 26,685 (48.0) | 3,115 | 11.7 | 1 |  |
| Present | 75,855 (45.9) | 5,020 | 6.6 | 1.00 (0.96,1.05) | 0.831 | 28,915 (52.0) | 2,735 | 9.5 | 1.00 (0.94,1.07) | 0.991 |
| Type 1 Diabetes |  |  |  |  |  |  |  |  |  |  |
| Absent | 163,960 (99.2) | 12,435 | 7.6 | 1 |  | 54,775 (98.5) | 5,770 | 10.5 | 1 |  |
| Present | 1,310 (0.8) | 105 | 8.0 | 0.95 (0.77,1.15) | 0.602 | 825 (1.5) | 80 | 9.7 | 0.90 (0.70,1.13) | 0.705 |
| Type 2 Diabetes |  |  |  |  |  |  |  |  |  |  |
| Absent | 81,205 (49.1) | 7,270 | 9.0 | 1 |  | 33,200 (59.7) | 3,875 | 11.7 | 1 |  |
| Present | 84,060 (50.9) | 5,270 | 6.3 | 0.84 (0.80,0.87) | <0.001 | 22,400 (40.3) | 1,975 | 8.8 | 0.88 (0.83,0.94) | <0.001 |
| Cardiovascular Disease |  |  |  |  |  |  |  |  |  |  |
| Absent | 139,690 (84.5) | 10,780 | 7.7 | 1 |  | 50,115 (90.1) | 5,295 | 10.6 | 1 |  |
| Present | 25,575 (15.5) | 1,760 | 6.9 | 1.16 (1.10,1.23) | <0.001 | 5,485 (9.9) | 550 | 10.0 | 1.19 (1.08,1.31) | <0.001 |
| Learning Disability |  |  |  |  |  |  |  |  |  |  |
| Absent | 162,845 (98.5) | 12,270 | 7.5 | 1 |  | 54,840 (98.6) | 5,755 | 10.5 | 1 |  |
| Present | 2,425 (1.5) | 270 | 11.1 | 1.16 (1.01,1.32) | 0.031 | 760 (1.4) | 95 | 12.5 | 1.06 (0.84,1.31) | 0.600 |
| Depression |  |  |  |  |  |  |  |  |  |  |
| Absent | 133,545 (80.8) | 9,635 | 7.2 | 1 |  | 44,725 (80.4) | 4,485 | 10.0 | 1 |  |
| Present | 31,725 (19.2) | 2,905 | 9.2 | 1.26 (1.21,1.32) | <0.001 | 10,875 (19.6) | 1,360 | 12.5 | 1.22 (1.14,1.30) | <0.001 |
| Dementia |  |  |  |  |  |  |  |  |  |  |
| Absent | 162,655 (98.4) | 12,330 | 7.6 | 1 |  | 54,625 (98.2) | 5,735 | 10.5 | 1 |  |
| Present | 2,610 (1.6) | 210 | 8.0 | 1.27 (1.10,1.47) | 0.001 | 975 (1.8) | 115 | 11.8 | 1.48 (1.20,1.81) | <0.001 |
| Serious Mental Illness |  |  |  |  |  |  |  |  |  |  |
| Absent | 158,655 (96.0) | 11,730 | 7.4 | 1 |  | 51,835 (93.2) | 5,260 | 10.1 | 1 |  |
| Present | 6,610 (4.0) | 810 | 12.3 | 1.68 (1.55,1.81) | <0.001 | 3,765 (6.8) | 590 | 15.7 | 1.63 (1.48,1.79) | <0.001 |
| Asthma |  |  |  |  |  |  |  |  |  |  |
| Absent | 131,465 (79.5) | 9,790 | 7.4 | 1 |  | 45,075 (81.1) | 4,650 | 10.3 | 1 |  |
| Present | 33,805 (20.5) | 2,750 | 8.1 | 1.09 (1.04,1.14) | <0.001 | 10,525 (18.9) | 1,200 | 11.4 | 1.08 (1.01,1.16) | 0.018 |
| COPD |  |  |  |  |  |  |  |  |  |  |
| Absent | 160,075 (96.9) | 12,165 | 7.6 | 1 |  | 53,955 (97.0) | 5,680 | 10.5 | 1 |  |
| Present | 5,190 (3.1) | 375 | 7.2 | 1.19 (1.07,1.33) | 0.001 | 1,645 (3.0) | 170 | 10.3 | 1.22 (1.04,1.44) | 0.017 |
| Stroke and TIA |  |  |  |  |  |  |  |  |  |  |
| Absent | 157,710 (95.4) | 11,945 | 7.6 | 1 |  | 53,085 (95.5) | 5,605 | 10.6 | 1 |  |
| Present | 7,560 (4.6) | 595 | 7.9 | 1.28 (1.18,1.40) | <0.001 | 2,520 (4.5) | 240 | 9.5 | 1.09 (0.94,1.25) | 0.247 |

Extreme acceleration in rate of weight gain is defined as δ-change ≥ 1.84 kilograms (kg)/metre squared(m^2^)/year. δ-change refers to the change (δ) in rate of weight gain between the prepandemic (δ-prepandemic) and pandemic (δ-pandemic) periods: δ-change = δ-pandemic - δ-prepandemic. N (%): Number (and percentage) of individuals within population subgroups. n: number within each population subgroup that experienced extreme acceleration in rate of weight gain. %: percentage of each group that experienced extreme acceleration. aOR: adjusted Odds Ratio of extreme acceleration in rate of weight gain adjusted for age, sex and IMD. aOR for long term conditions presented in comparison to a reference group without the condition. CI: confidence interval, IMD: Index of Multiple Deprivation, COPD: Chronic Obstructive Pulmonary Disease. TIA: Transient Ischaemic Attack.
