# Supplementary material for: Trends in weight gain recorded in English primary care before and during the Coronavirus-19 pandemic: An observational cohort study using the OpenSAFELY platform
Source: PLoS Med. 2024 Jun 24;21(6):e1004398. doi: 10.1371/journal.pmed.1004398 (PMC11249215; doi:10.1371/journal.pmed.1004398)
Supplement: S9 Table — (DOCX) [file pmed.1004398.s014.docx]

S9 Table. Associations between sociodemographic and clinical characteristics and odds of extreme acceleration in rate of weight gain during the pandemic in analysis stratified by Index of Multiple Deprivation (IMD)

|  | IMD1 (Most Deprived) | | | | | IMD5 (Least Deprived) | | | | |
| --- | --- | --- | --- | --- | --- | --- | --- | --- | --- | --- |
|  | N (%) | Extreme Acceleration | | | | N (%) | Extreme Acceleration | | | |
|  |  | n | % | aOR | p |  | n | % | aOR | p |
| Sex |  |  |  |  |  |  |  |  |  |  |
| Female | 355,210 (59.9) | 47,510 | 13.4 | 1 |  | 270,215 (57.2) | 26,570 | 9.8 | 1 |  |
| Male | 237,595 (40.1) | 21,655 | 9.1 | 0.73 (0.71,0.74) | <0.001 | 202,585 (42.8) | 13,420 | 6.6 | 0.72 (0.70,0.74) | <0.001 |
| Age Group (in years) |  |  |  |  |  |  |  |  |  |  |
| 18-29 | 39,950 (6.7) | 6,975 | 17.5 | 1 |  | 24,620 (5.2) | 2,970 | 12.1 | 1 |  |
| 30-39 | 67,950 (11.5) | 10,785 | 15.9 | 0.94 (0.91,0.97) | <0.001 | 35,030 (7.4) | 4,465 | 12.7 | 1.08 (1.03,1.14) | 0.002 |
| 40-49 | 81,210 (13.7) | 10,590 | 13.0 | 0.81 (0.78,0.84) | <0.001 | 45,505 (9.6) | 4,535 | 10.0 | 0.86 (0.82,0.90) | <0.001 |
| 50-59 | 118,835 (20.0) | 14,115 | 11.9 | 0.73 (0.70,0.75) | <0.001 | 71,985 (15.2) | 6,675 | 9.3 | 0.84 (0.80,0.88) | <0.001 |
| 60-69 | 124,975 (21.1) | 12,830 | 10.3 | 0.62 (0.60,0.64) | <0.001 | 94,455 (20.0) | 7,755 | 8.2 | 0.76 (0.72,0.79) | <0.001 |
| 70-79 | 108,550 (18.3) | 9,580 | 8.8 | 0.51 (0.49,0.52) | <0.001 | 128,515 (27.2) | 8,840 | 6.9 | 0.62 (0.59,0.65) | <0.001 |
| 80-90 | 51,340 (8.7) | 4,290 | 8.4 | 0.47 (0.45,0.49) | <0.001 | 72,695 (15.4) | 4,760 | 6.5 | 0.58 (0.55,0.61) | <0.001 |
| Ethnicity |  |  |  |  |  |  |  |  |  |  |
| White | 480,580 (81.1) | 58,840 | 12.2 | 1 |  | 449,885 (95.2) | 38,275 | 8.5 | 1 |  |
| Black | 23,530 (4.0) | 2,580 | 11.0 | 0.84 (0.81,0.88) | <0.001 | 2,910 (0.6) | 320 | 11.0 | 1.24 (1.10,1.39) | <0.001 |
| South Asian | 62,560 (10.6) | 5,100 | 8.2 | 0.60 (0.58,0.62) | <0.001 | 10,545 (2.2) | 715 | 6.8 | 0.75 (0.69,0.81) | <0.001 |
| Mixed | 7,175 (1.2) | 900 | 12.5 | 0.90 (0.84,0.96) | 0.003 | 2,505 (0.5) | 215 | 8.6 | 0.87 (0.75,1.00) | 0.055 |
| Chinese/Other | 18,960 (3.2) | 1,745 | 9.2 | 0.69 (0.66,0.73) | <0.001 | 6,955 (1.5) | 470 | 6.8 | 0.73 (0.67,0.81) | <0.001 |
| Long Term Condition | |  |  |  |  |  |  |  |  |  |
| Hypertension |  |  |  |  |  |  |  |  |  |  |
| Absent | 310,530 (52.4) | 40,180 | 12.9 | 1 |  | 226,165 (47.8) | 20,850 | 9.2 | 1 |  |
| Present | 282,275 (47.6) | 28,985 | 10.3 | 1.04 (1.02,1.06) | <0.001 | 246,635 (52.2) | 19,145 | 7.8 | 1.12 (1.09,1.15) | <0.001 |
| Type 1 Diabetes |  |  |  |  |  |  |  |  |  |  |
| Absent | 584,060 (98.5) | 68,130 | 11.7 | 1 |  | 463,390 (98.0) | 39,235 | 8.5 | 1 |  |
| Present | 8,745 (1.5) | 1,035 | 11.8 | 0.94 (0.88,1.00) | 0.059 | 9,410 (2.0) | 755 | 8.0 | 0.90 (0.84,0.97) | 0.007 |
| Type 2 Diabetes |  |  |  |  |  |  |  |  |  |  |
| Absent | 402,955 (68.0) | 50,455 | 12.5 | 1 |  | 350,905 (74.2) | 30,065 | 8.6 | 1 |  |
| Present | 189,850 (32.0) | 18,710 | 9.9 | 0.97 (0.95,0.99) | 0.006 | 121,895 (25.8) | 9,925 | 8.1 | 1.17 (1.15,1.20) | <0.001 |
| Cardiovascular Disease | |  |  |  |  |  |  |  |  |  |
| Absent | 487,765 (82.3) | 58,615 | 12.0 | 1 |  | 386,645 (81.8) | 33,640 | 8.7 | 1 |  |
| Present | 105,040 (17.7) | 10,550 | 10.0 | 1.08 (1.05,1.10) | <0.001 | 86,155 (18.2) | 6,355 | 7.4 | 1.09 (1.06,1.13) | <0.001 |
| Learning Disability |  |  |  |  |  |  |  |  |  |  |
| Absent | 577,975 (97.5) | 67,035 | 11.6 | 1 |  | 467,665 (98.9) | 39,380 | 8.4 | 1 |  |
| Present | 14,830 (2.5) | 2,130 | 14.4 | 1.13 (1.08,1.19) | <0.001 | 5,135 (1.1) | 610 | 11.9 | 1.28 (1.17,1.39) | <0.001 |
| Depression |  |  |  |  |  |  |  |  |  |  |
| Absent | 393,500 (66.4) | 41,505 | 10.5 | 1 |  | 361,765 (76.5) | 27,900 | 7.7 | 1 |  |
| Present | 199,305 (33.6) | 27,660 | 13.9 | 1.23 (1.21,1.25) | <0.001 | 111,035 (23.5) | 12,090 | 10.9 | 1.36 (1.33,1.39) | <0.001 |
| Dementia |  |  |  |  |  |  |  |  |  |  |
| Absent | 582,520 (98.3) | 67,790 | 11.6 | 1 |  | 464,200 (98.2) | 39,025 | 8.4 | 1 |  |
| Present | 10,285 (1.7) | 1,375 | 13.4 | 1.59 (1.50,1.69) | <0.001 | 8,600 (1.8) | 965 | 11.2 | 1.75 (1.63,1.88) | <0.001 |
| Serious Mental Illness | |  |  |  |  |  |  |  |  |  |
| Absent | 565,585 (95.4) | 64,950 | 11.5 | 1 |  | 463,300 (98.0) | 38,730 | 8.4 | 1 |  |
| Present | 27,220 (4.6) | 4,215 | 15.5 | 1.40 (1.35,1.45) | <0.001 | 9,500 (2.0) | 1,260 | 13.3 | 1.60 (1.50,1.69) | <0.001 |
| Asthma |  |  |  |  |  |  |  |  |  |  |
| Absent | 442,175 (74.6) | 49,415 | 11.2 | 1 |  | 370,555 (78.4) | 30,500 | 8.2 | 1 |  |
| Present | 150,630 (25.4) | 19,750 | 13.1 | 1.12 (1.10,1.14) | <0.001 | 102,245 (21.6) | 9,495 | 9.3 | 1.07 (1.05,1.10) | <0.001 |
| COPD |  |  |  |  |  |  |  |  |  |  |
| Absent | 520,565 (87.8) | 61,320 | 11.8 | 1 |  | 443,150 (93.7) | 37,440 | 8.4 | 1 |  |
| Present | 72,240 (12.2) | 7,845 | 10.9 | 1.09 (1.06,1.12) | <0.001 | 29,650 (6.3) | 2,550 | 8.6 | 1.24 (1.18,1.29) | <0.001 |
| Stroke and TIA |  |  |  |  |  |  |  |  |  |  |
| Absent | 553,750 (93.4) | 65,000 | 11.7 | 1 |  | 439,290 (92.9) | 37,330 | 8.5 | 1 |  |
| Present | 39,055 (6.6) | 4,165 | 10.7 | 1.13 (1.09,1.16) | <0.001 | 33,510 (7.1) | 2,660 | 7.9 | 1.16 (1.11,1.21) | <0.001 |

Extreme acceleration in rate of weight gain is defined as δ-change ≥ 1.84 kilograms (kg)/metre squared(m^2^)/year. δ-change refers to the change (δ) in rate of weight gain between the prepandemic (δ-prepandemic) and pandemic (δ-pandemic) periods: δ-change = δ-pandemic - δ-prepandemic. N (%): Number (and percentage) of individuals within population subgroups. n: number within each population subgroup that experienced extreme acceleration in rate of weight gain. %: percentage of each group that experienced extreme acceleration. aOR: adjusted Odds Ratio of extreme acceleration in rate of weight gain adjusted for age, sex, and ethnicity. aOR for long term conditions presented in comparison to a reference group without the condition. CI: confidence interval, IMD: Index of Multiple Deprivation, COPD: Chronic Obstructive Pulmonary Disease. TIA: Transient Ischaemic Attack.
