# Supplementary material for: Trends in weight gain recorded in English primary care before and during the Coronavirus-19 pandemic: An observational cohort study using the OpenSAFELY platform
Source: PLoS Med. 2024 Jun 24;21(6):e1004398. doi: 10.1371/journal.pmed.1004398 (PMC11249215; doi:10.1371/journal.pmed.1004398)
Supplement: Attachment — Submitted filename: Comments (PMEDICINE-D-23-01514).pdf [file pmed.1004398.s015.pdf]

Comments (PMEDICINE-D-23-01514R1)

It's a pleasure to review this manuscript. Overall, it's an interesting study with a well written description and explanation. Several comments to improve the manuscript:

**Section**	**Subsection**	**Line**	**Comment**
Background		11	What does it mean 'living in deprivation'?
Method	Study population		Any exclusion criteria for this study?
	Study outcomes	3	Kindly explain how the BMI was recorded opportunistically.
	Individual level BMI trajectories in pre-pandemic and pandemic periods	3	How did you select BMI measures randomly?
		14	The full name for IMD abbreviation should be introduced here.
	Defining a population of extreme accelerators	5	Please clarify<br>"if patients were losing weight prepandemic, the rate of weight loss slowed."
Results	Rapid weight gain (> 0·5kg/m2 /year) in prepandemic and pandemic time periods	Title	Suggestion to choose either time or periods in the subsection title as they depict the same meaning.
Conclusion			Suggestion to put the discussion (under discussion section) separated from the conclusion.
