# Supplementary material for: Trends in weight gain recorded in English primary care before and during the Coronavirus-19 pandemic: An observational cohort study using the OpenSAFELY platform
Source: PLoS Med. 2024 Jun 24;21(6):e1004398. doi: 10.1371/journal.pmed.1004398 (PMC11249215; doi:10.1371/journal.pmed.1004398)
Supplement: Attachment — Submitted filename: samuel.pdf [file pmed.1004398.s017.pdf]

This paper describes an analysis of weight gain during the period of the COVID-19 outbreak using an existing English cohort.

I was asked for a statistical report and I interpret that to include all aspects of the design and conduct of the study. I note that this has already gone through one round of review but I have not seen it before. I have reviewed it from scratch without viewing the previous comments and changes. I have then appended any comments relating to previous reviews.

1 Review masked to previous reviews and rebuttal

1.1 Points of detail

Page 2 What is TPP?

Page 2 Strictly speaking multivariable is meant here (Hidalgo and Goodman, 2013). Multivariate means multiple variables on the left hand side. I do not think that is what the authors did.

Page 4 For readers not familiar with the platform the data source section is rather scant. Where did all this primary care data come from? Is it from one of the existing primary care databases? What validation has been performed on it?

Page 5 I have only looked at a few of the files on GitHub and, obviously, not run them but they do appear appropriately commented.

Page 5 How many were excluded as BMI outliers? This could go in Figure 1. Apologies if I have missed it somewhere else but it does belong in the flowchart.

Page 6 A citation to the package used (dplyr) would be appropriate. It is the only credit authors get (disclaimer: I am a package author but not of any packages involved here).

Page 6 Why was it felt necessary to categorise age? Categorising an essentially continuous variable wastes information (Altman and Royston, 2006; Royston et al., 2006) and leads to models which are often implausible as they predict the effect remaining flat within categories and then jumping to a new value at the category boundary. From the plots a simple function of age would have been sufficient.

Page 6 What does chronic cardiac conditions cover?

Page 6 I do not understand why the authors used two different ways of assigning age. My experience is that researchers usually use age at cohort entry but age at study exit can be justified if it makes the interpretation more sensible.

Page 7 Where does the ethnic coding come from? Do general practitioners actually use the census categories? There is no mention of the database being linked to ONS census data. The White population of England seems to have been 85% in 2011 and 81% in 2021 both somewhat higher than that given here. Why is that?

Page 10 Why present decide to present a comparison of 18–29 year olds specifically versus 50-59?

Page 11 In the caption to Figure 4 it is unnecessary to spell out kg or m.

Page 11 I could not find the sensitivity analysis explained in the methods and I can only understand this if there were in fact two analyses as I cannot otherwise grasp how the results for Black people could be modified by including people with missing ethnicity.

Page 12 The authors claim that previous studies have used non–representative samples. For various reasons outlined by the authors themselves on page 13 the fact that people had to have their weight recorded must undermine any very strong claim to representativeness. Their sample may well approximate a random sample of adults living in England more closely than other studies. I am not blaming the authors here, when using routine data we can only analyse what other people have recorded not what we would have liked them to record.

Figure 1 It would be helpful to add the numbers still included in all the boxes down the main path to provide a basis for the importance of the exclusions.

Table S6 The column widths need adjusting here. Splitting the confidence interval over two table rows may be unavoidable but splitting in the middle of a number is super–ugly. Tables S7 to S9 do not share this problem.

I found the use of δ for change confusing as Δ is almost universal in mathematics for change.

1.2 Point of more substance

It might be helpful to on page 5 to clarify what original models the minimally adjusted models replaced. It would have seemed strange to present an

analysis like this without adjusting for age and sex. What model underlies the results on, for example, page 10. Is each just adjusted for age and sex or for all the others in the table? The captions seem to suggests that each is only adjusted for age and sex but not for the others. That seems a strange decision to me. If we just take the long term conditions it is now generally accepted to people with SMI have poor physical health and reduced life expectancy. So any coefficient for SMI also represents the effect of other long term conditions.

2 Comments on previous reviews and rebuttal

After completing the above review I looked at the previous reviews and the rebuttal.

2.1 Adjustment of the covariates

I clearly disagree with some of the reviewers' comments over minor matters but the major point of issue is the decision to move from a fully adjusted model to one just using age and sex as covariates. If you have a number of potential covariates which are associated then presenting each unadjusted for the others means that you are potentially reporting an inflated number of associations. There is also a danger that if you collapse over important covariates you run the risk of Simpson's paradox (Simpson, 1951).

The issue seems particularly important with the long term conditions. As I mentioned in my original comments reporting the coefficient for SMI unadjusted is also mixing in associations with other illnesses. The same is probably true for other conditions.

I imagine similar concerns could be raised about area deprivation and ethnicity.

2.2 Baseline BMI

I see it has been suggested that the models should include baseline BMI. I agree that this would not be helpful. If we denote the ith measurement of BMI as Y_i and denote the covariates by X then, ignoring error terms and subscripts, the authors' current model for change in weight gain is

$$Y_1 - 2Y_2 + Y_3 = BX \qquad (1)$$

inserting baseline BMI on the righthand side as suggested

$$Y_1 - 2Y_2 + Y_3 = b_y Y_1 + BX \tag{2}$$

changing sides and simplifying

$$(1 - b_y)Y_1 - 2Y_2 + Y_3 = BX \tag{3}$$

I find it hard to see equation 3 as corresponding to a useful scientific or clinical hypothesis. Note that equation 1 models the quadratic association between time and BMI since (1, -2, 1) are the coefficients of the orthogonal polynomial corresponding to a quadratic (orthogonal to the linear (-1, 0, 1)).

It would be perfectly possible to have a model incorporating baseline BMI to predict post–pandemic BMI gain.

$$Y_3 - Y_2 = b_Y Y_1 + BX \tag{4}$$

which would relate to the question "Was pre–pandemic overweight/obesity associated with gaining or losing BMI during the pandemic" but that is not, I think, the authors' concern here.

3 Summary

Some food for thought.

Michael Dewey

References

D G Altman and P Royston. The cost of dichotomising continuous variables. *British Medical Journal*, 332:1080, 2006.

B Hidalgo and M Goodman. Multivariate or multivariable regression. *American Journal of Public Health*, 103:39–40, 2013.

P Royston, D G Altman, and W Sauerbrei. Dichotomizing continuous predictors in multiple regression: a bad idea. *Statistics in Medicine*, 25:127–141, 2006.

E H Simpson. The interpretation of interaction in contingency tables. *Journal of the Royal Statistical Society — Series B*, pages 238–241, 1951.
