# Supplementary material for: Trends in weight gain recorded in English primary care before and during the Coronavirus-19 pandemic: An observational cohort study using the OpenSAFELY platform
Source: PLoS Med. 2024 Jun 24;21(6):e1004398. doi: 10.1371/journal.pmed.1004398 (PMC11249215; doi:10.1371/journal.pmed.1004398)
Supplement: Attachment — Submitted filename: Responses to Request from editors.docx [file pmed.1004398.s016.docx]

Dear PLOS Medicine Editorial team,

Thank you for giving us the opportunity to submit a revised manuscript. We truly appreciate the positive comments and helpful suggestions from the reviewers and editorial team. We strongly believe that thanks to the questions, comments, and suggestions we have greatly enhanced this revised manuscript to make it clearer and more concise.

We have addressed the specific request and comments made by the reviewers and editors in this response letter and edited the manuscript, tables, figures, and supplementary information files accordingly. We have also pointed out the changes we have made in the manuscript and indicated the location where the changes can be found (including line numbers).

You will appreciate that we have reflected on the comments made by the academic editor and agree that presenting minimally adjusted models is more in keeping with the descriptive focus of this study. We have therefore updated the manuscript to include the results of logistics regression models that are only adjusted for age and sex. This has led to numerous changes in the adjusted Odds Ratios (aORs) presented in the results, figures, and supplementary tables. Notably, although specific aORs and corresponding confidence intervals have varied from those presented in the original manuscript, there was little impact on the size or direction of effect. Therefore our summary findings and conclusions have remained consistent between the original and revised manuscript.

Another notable change is the increase in supplementary material we have submitted. In order to comply with PLOS figure guidelines, we had to reduce the data presented in the figs 2-4, to prevent the figures from being too long. All data removed from these figures has been moved to additional supplementary tables.

A minor change in the manuscript (and in the manuscript data) not specifically referred to below is a change in affiliation of Professor Valabhji from ‘Imperial College Healthcare’ Trust to ‘Imperial College London’.

Thank you very much again for giving us the opportunity to respond to reviewer comments. We do believe the process has improved our work and look forward to hearing from you at your earliest convenience.

Your Sincerely,

Dr Miriam Samuel

**ACADEMIC EDITOR COMMENTS**

**Comment 1**

**While the authors state that "this is a descriptive study," one could argue that there is a causal tinge to the analysis. Some of the comments by Reviewer 2 and 3 are getting to this point in the questioning of covariates and requesting to include baseline BMI in the models.**

**Would the paper be better framed descriptively by prioritizing simpler unadjusted or age-adjusted or or stratified models, rather than fully multivariable models? What do the fully adjusted models add? For example, what are readers to make of models that adjust for both ethnicity and deprivation? This might diminish the extend of disparities that are observed from a descriptive perspective.

It seems hard to interpret each individual odds ratio in any way that is not casually influenced and susceptible to the Table 2 fallacy.**

Thank you for this comment which has been a very helpful in providing a focus to how the results have been presented. As suggested. we have updated the manuscript to present minimally adjusted statistical models adjusted only for age and sex in all individual level BMI trajectory analyses (rather than the age, sex, Index of Multiple Deprivation (IMD), and ethnicity adjusted models we previously presented). We have described that we have made this change in response to peer review in the methods (line 178 – 179) and updated the statistical methods (line 286-287). Changes in the results can be seen in the following results sections: Rapid weight gain (> 0.5kg/m^2^/year) in prepandemic and pandemic periods, extreme acceleration in rate of weight gain between the prepandemic and pandemic and sensitivity and subgroup analyses (lines 334 – 406). Figure 3 and 4 have also been updated, as have supplementary tables S4-9. Notably, there was little difference in the effect estimates between the models adjusted for age and sex presented in the current version of the manuscript and the models adjusted for age, sex, IMD and ethnicity presented in the original manuscript.

**EDITORIAL COMMENTS**

**Comment 1**

**In your discussion, you refer to post-pandemic research, policies and interventions targeting BMI, and we suggest that you expand your discussion to include future pre-pandemic planning and preventive interventions.**

Thank you for this useful suggestion. We have added reference to the utility of these results in future pandemic planning in the abstract (lines 84-86), non-technical summary (lines 114 - 116) and the discussion (lines 500 - 502).

**Requests from editors**

**Request 1**

**Please include page numbers and line numbers in the manuscript file. Use continuous line numbers (do not restart the numbering on each page).**

Thank you. We have revised the manuscript to include line numbers as suggested.

**Request 2**

**Please cite the reference numbers in square brackets (e.g., “We used the techniques developed by our colleagues [19] to analyze the data”). Citations should be preceding punctuation.**

Thank you. We have changed the citation style in keeping with PLOS guidelines as suggested.

**Request 3
Please cite your Supporting Information as outlined here:** [**https://journals.plos.org/plosmedicine/s/supporting-information**](https://journals.plos.org/plosmedicine/s/supporting-information)

Thank you. We have updated the naming convention and description in the text in line with the listed guidelines.

**Request 4
Please remove the sections ‘Role of Funding Source’, ‘Declaration of Interests’, ‘Data Sharing’ from the main manuscript. This information should only be included in the according section in the online submission form.**

Thank you. We have removed these from the manuscript as requested.

**Request 5**

**Please ensure consistency in your number format and revise your manuscript accordingly including the Supplementary Material (51·4% or 51.4%; 1000 or 1,000).**

Thank you. We have reviewed the text to ensure consistency in the number format as requested.

**Request 6
We have noticed that you mostly define the time periods examined in your study as "pandemic" and "pre-pandemic", whereas in some figure/table titles you refer to the time periods as "before and after the onset of the COVID-19 pandemic". We suggest that you adopt a consistent format for clarity for the reader.**

Thank you for highlighting this inconsistency. We have been more consistent with the use of prepandemic and pandemic periods.

**Request 6**

**Please comment on the definition of the age groups studied in your study. You have stated that "data were extracted on all adults aged ≥18 to ≤90 years [...]" but then define the oldest age group as "80-89". In some of the supplementary tables, '90' appears to be a single age group, but not, for example, in Table 1, which shows the baseline characteristics of the study population.**

Thank you for highlighting this inconsistency. The inclusion criteria was age ≥18 to ≤90 years. We have updated this on all tables, figures and in the text.

FINANCIAL DISCLOSURE

**Request 7**

**The funding statement should include: specific grant numbers, initials of authors who received each award, URLs to sponsors’ websites.**

Thank you for this comment. We have updated the financial disclosure section to reflect specific grant numbers and URLs to specific websites where available. This has been removed from the main manuscript as suggested by the editors.

ABSTRACT

**Request 8**

**Please structure your abstract using the PLOS Medicine headings (Background, Methods and Findings, Conclusions). Please combine the Methods and Findings sections into one section, “Methods and findings”.**

Thank you for this comment. We have changed the structure of the abstract as per the PLOS Medicine guidelines.

**Request 9**

**PLOS Medicine requests that main results are quantified with 95% Cis as well as p values. When reporting p values please report as p<0.001 and where higher as the exact p value p=0.002, for example. For the purposes of transparent data reporting, if not including the aforementioned please clearly state the reasons why not.**

Thank you. We have updated all results to show 95% CIs and p-values to three decimal places when a comparison is being made between two groups.

**Request 10**

**Please include any important dependent variables that are adjusted for in the analyses.**

Thank you. All regression models have been updated in response to the academic editor’s comments. We have stated in the abstract methods that regression models were adjusted for age and sex (line 63-65).

**Request 11**

**Throughout, suggest reporting statistical information as follows to improve clarity for the reader “22% (95% CI [13%,28%]; p
Please note the use of commas to separate upper and lower bounds, as opposed to hyphens as these can be confused with reporting of negative values.**

Thank you. We have updated all statistical information to follow this convention when a comparison is being made between groups.

**Request 12**

**Please define all abbreviations used for statistical reporting at first use.**

We have now ensured abbreviations are described in full. Notably ‘The OpenSAFELY platform inside TPP’ is the naming convention for the platform used and cannot be expanded. TPP is the name of a specific healthcare software provided. We have explained this briefly within the methods section in the manuscript (line 188).

**Request 13**

**When a p value is given, please specify the statistical test used to determine it.**

Thank you. We have clarified in the abstract methods that p-values were generated in regression models (line 65).

**Request 14**

**Please define 'NHS‘, ‚IQR‘, ‚aOR’, ‘IMD’.**

Thank you. We have defined these in the abstract (NHS: line 52, Interquartile Range: line 65, aOR: line 67. IMD: line 70). NHS England is the full name of an organisation which overseas National Health Service (NHS) activity England. We have, however, adapted the final sentence of the abstract background to define NHS at first use and further clarify the study question (line 52).

“We investigate which clinical and sociodemographic characteristics were associated with rapid weight gain and the greatest acceleration in rate of weight gain during the pandemic amongst adults registered with an English National Health Service (NHS) general practitioner (GP) during the COVID-19 pandemic.”

**Request 15**

**Abstract Background: Provide the context of why the study is important. The final sentence should clearly state the study question.**

Thank you. We updated the abstract background in line with this guidance (line 49 – 52).

“We investigated which clinical and sociodemographic characteristics were associated with rapid weight gain and the greatest acceleration in rate of weight gain during the pandemic amongst adults registered with an English National Health Service (NHS) general practitioner (GP) during the COVID-19 pandemic.”

Abstract Methods and Findings:

**Request 16
* Please ensure that all numbers presented in the abstract are present and identical to numbers presented in the main manuscript text.**

Thank you for highlighting the presence of inconsistencies. We have reviewed the main text, figures and tables to ensure the numbers are identical.

**Request 17
* Please include the population (age range should be defined for ‘adults’) and setting.**

Thank you. We have added this to the abstract methods and findings (line 57).

**Request 18**

*** In the last sentence of the Abstract Methods and Findings section, please describe the main limitation(s) of the study's methodology.**

Thank you. We have included a sentence on the main limitations in the abstract methods and findings (line 77-78).

“However, changes in healthcare activity during the pandemic may have introduced new bias to the data.”

Abstract Conclusions:

**Request 19
* Please avoid vague statements such as "these results have major implications for policy/clinical care". Mention only specific implications substantiated by the results.**

Thank you. we have removed this sentence and updated with a sentence on the utility of these results, including their utility in future pandemic planning (line 84-86). This reflects a suggestion by the editor on the need to consider the results in the context of future pandemic planning in the discussion.

“Our findings highlight the need to incorporate sociodemographic, physical, and mental health characteristics when formulating research, policies, and interventions targeting BMI in the period of post pandemic service restoration and in future pandemic planning.’

AUTHOR SUMMARY

**Request 20**

**At this stage, we ask that you include a short, non-technical Author Summary of your research to make findings accessible to a wide audience that includes both scientists and non-scientists. The Author Summary should immediately follow the Abstract in your revised manuscript. This text is subject to editorial change and should be distinct from the scientific abstract. Please see our author guidelines for more information: https://journals.plos.org/plosmedicine/s/revising-your-manuscript#loc-author-summary.

The summary should include 2-3 single sentence, individual bullet points under each of the questions. The last bullet point should describe the main limitation(s) of the study's methodology.

It may be helpful to review currently published articles for examples which can be found on our website here** [**https://journals.plos.org/plosmedicine/**](https://journals.plos.org/plosmedicine/)

Thank you. We have included a non-technical summary after the abstract in line with the guidelines above and on the PLOS Medicine website. We have removed the ‘summary box’ section from the manuscript as this followed a different format. (line 88 – 121).

INTRODUCTION

**Request 21**

**Please remove the study results and/or conclusion from the Introduction and conclude the Introduction with a clear description of the study question or hypothesis.**

Thank you. We have removed the last sentence and concluded with a description of the study question (p4, line 150 – 155).

“Using routinely collected English primary care records we conducted an observational cohort study to: (i) describe population-level changes in BMI recorded in healthcare records during the pandemic and (ii) estimate individual-level rates of weight gain before and during the pandemic to identify how (iia) risk of rapid weight gain (>0.5kg/m^2^/year) in the pre-pandemic and pandemic periods and (iib) risk of extreme acceleration in the rate of weight gain (≥1.84kg/m2/year) between the prepandemic and pandemic period varied by sociodemographic and clinical characteristics.”

METHODS AND RESULTS

**Request 22**

**Please ensure that the study is reported according to the RECORD guideline, and include the completed RECORD checklist as Supporting Information. When completing the checklist, please use section and paragraph numbers, rather than page numbers. Please add the following statement, or similar, to the Methods: "This study is reported as per the REporting of studies Conducted using Observational Routinely-collected health Data (RECORD) Statement (S1 Checklist)."**

Thank you for this suggestion. We have completed the RECORD checklist as S1 Checklist and included the following sentence in the Methods "This study is reported as per the REporting of studies Conducted using Observational Routinely-collected health Data (RECORD) Statement (S1 Checklist)." (line 178 – 179)

**Request 23**

**Did your study have a prospective protocol or analysis plan? Please state this (either way) early in the Methods section.**

The study did have a prospective protocol which we have included in the revised manuscript. (S1 protocol – line 169 -170).

**Request 24**

**a) If a prospective analysis plan (from your funding proposal, IRB or other ethics committee submission, study protocol, or other planning document written before analyzing the data) was used in designing the study, please include the relevant prospectively written document with your revised manuscript as a Supporting Information file to be published alongside your study, and cite it in the Methods section. A legend for this file should be included at the end of your manuscript.
b) If no such document exists, please make sure that the Methods section transparently describes when analyses were planned, and when/why any data-driven changes to analyses took place.
c) In either case, changes in the analysis-- including those made in response to peer review comments-- should be identified as such in the Methods section of the paper, with rationale.**

**For all observational studies, in the manuscript text, please indicate: (1) the specific hypotheses you intended to test, (2) the analytical methods by which you planned to test them, (3) the analyses you actually performed, and (4) when reported analyses differ from those that were planned, transparent explanations for differences that affect the reliability of the study's results. If a reported analysis was performed based on an interesting but unanticipated pattern in the data, please be clear that the analysis was data-driven.**

Thank you for these suggestions. We have included a new paragraph in the Methods section called ‘Analysis Plan’ which includes the study protocol and a written description of how the presented analysis differs to this, including the changes made in response to peer review (line 167 -179).

**“Analysis Plan**

We conducted an observational cohort study following the analysis plan set out in the prospective protocol (S1 protocol). The study aimed to evaluate, amongst adults living in the United Kingdom, how trends in measures of weight have changed since the onset of the COVID-19 pandemic using data routinely collected in primary care electronic health records (EHRs). The analysis was subsequently limited to adults living in England due to data availability through the analysis platform. Due to the size and complexity of the dataset the protocol was also adapted in order to facilitate the analysis within the computational capacity. Specifically, based on literature review and consensus from a clinical advisory panel, we refined the covariates included in the analysis and adjusted the age range of the population studied (from >18 to ≤110 years in the protocol to >18 to ≤90 years in the completed analysis). Following peer review, we have presented the results of minimally adjusted statistical models (adjusted for age and sex) in line with the descriptive focus of the analysis. This study is reported as per the REporting of studies Conducted using Observational Routinely-collected health Data (RECORD) Statement (S1 Checklist).”(lines 167 – 179)

As this was an exploratory descriptive study, the protocol states that the methodology used will be adapted based on the structure and availability of the data. To ensure transparency, the methods section presents specific paragraphs on each part of the analysis with clear paragraph headings for clarity. Each section defines how decisions related to the analysis were made and clarifies when decisions were based on data-driven approaches and when decisions were guided by a clinical consensus panel (lines 191 – 265).

**Request 25**

**PLOS Medicine requests that main results are quantified with 95% CIs as well as p values. When reporting p values please report as p<0.001 and where higher as the exact p value p=0.002, for example. For the purposes of transparent data reporting, if not including the aforementioned, please clearly state the reasons why not.**
Thank you. We have presented all adjusted odds ratios following these conventions. We have clarified how prevalence estimates are presented in the methods as follows.

“Due to the very large sample size of the study population, 95% confidence intervals for the prevalence estimates of each of the individual-level outcomes were often the same as the point estimate. We therefore present the point estimate for prevalence estimates, accompanied by the adjusted Odds Ratio (aOR, adjusted for age and sex) with 95% confidence intervals for the aOR when a comparison is being made between groups.” (lines 286 – 290).

**Request 26**

**Please include any important dependent variables that are adjusted for in the analyses.**
Thank you. We have clarified methods to state we have presented the findings of models adjusted for age and sex. “As a descriptive study we have present the findings of minimally adjusted multivariable models adjusted for age and sex.” (lines 286 – 287).

**Request 27
Suggest reporting statistical information as detailed above – see under ABSTRACT**

Thank you. We have reported statistical information as described above under ABSTRACT.

**Request 28**

**Please present numerators and denominators for percentages, at least in the Tables [not necessarily each time they're mentioned].**

Thank you. We have added the numerator and denominator information to all tables.

**Request 29**

**In your Methods section, please briefly explain what TPP is.**

Thank you. We have updated the Methods - Study Population to state that TPP is a healthcare technology provider (line 188).

**Requests 30 – 36**

Thank you for picking up these specific points. We have corrected them as specified below.

**p.4: Please define ‘GP’.** (line 198) **p.4: It appears that “(Supplementary Appendix 2)” was mistakenly cited following a period. Please revise throughout your entire manuscript.** Changed to S2 Appendix (line 238). **p.5: Please use a consistent spelling for the word ‘socio-demographic’ (versus ‘sociodemographic).** Thank you. We have revised the manuscript to be more consistent in using the term sociodemographic. **p.5: Please define ‘IMD’ at first use and briefly explain what IMD is.** Updated in line 251 “Index of Multiple Deprivation (IMD)” and line 257 “IMD is an area-based marker of deprivation based on an individual’s place of residence.”  **p.5: For ”δ-change >= 1·84kg/m2/year)”, please use a greater-than-or-equal sign and revise throughout your entire manuscript (including the Supplementary material).** Thank you we have revised this. **p.10: Please define ‘SD’, ‘aOR’.** Thank you these are defined in line 340 (Standard Deviation (SD)) and in line 290 (adjusted Odds Ratio (aOR, adjusted for age and sex)). **p.10: Please, following ‘younger adults’ add an age range in brackets for clarity.** Thank you, we have clarified this as younger adults (18-29 years) in lines 92, 102, 377, and 417.

DISCUSSION

**Request 37**

**p.13: Please define ‘MNAR’.**

Thank you. As we only use this term once we expand it to ‘Missing Not at Random’ (line 492).

TABLES

**Request 38**

**Please note the use of commas to separate upper and lower bounds, as opposed to hyphens as these can be confused with reporting of negative values. Suggest reporting statistical information as detailed above – see under ABSTRACT.**

Thank you. We have updated all the main and supplementary tables with these guidelines.

**Request 39
Please define abbreviations used in the tables (including those in Supporting Information files).
Table 1: Please define ‘IMD’, ‘TIA’.
Table 1: It appears you have included the table title and the definitions for the asterisks (table description) twice. Please check and revise.**

Thank you. We have updated all the tables in line with the academic editor and reviewer inputs. We have clarified these points in the updated tables.

**Request 40
Table 1: Why is the oldest age group defined as ’80-89’ but the table description mentions that “Data were extracted on all adults aged >18 to ≤ 90 years […]”? This would mean the oldest age group would need to be defined as ’80-90’. Please revise throughout your entire manuscript.**

Thank you. We have updated all the age categories in all tables to reflect the inclusion criteria of 80-90.

FIGURES

**Request 50**

**For all Figures, please ensure that you have complied with our figures requirements http://journals.plos.org/plosmedicine/s/figures.

Please define abbreviations used in the figure legend of each figure and/or table (including those in Supporting Information files).

Please consider avoiding the use of red and green in order to make your figure more accessible to those with colour blindness.**
Thank you. We have changed all the figures to comply with the PLOS figure requirements. This has involved reducing the size of all the figures which has meant we have had to alter the amount of information presented in each figure. Specifically we have had to remove the findings for individuals of self-reported mixed ethnicity from the figures as the figures would have been longer than allowed. This is discussed in the figure legends and all data is available in the supplementary tables. We have removed red and green.

**Request 51**
**Figure 1: Please define ‘GP’, ‘IMD’, ‘kg’, ‘m’.

Figure 2: Please add a unit for ‘age group’. Please define ‘TIA’, ‘IQR’.
Figure 2: Please indicate in the figure caption the meaning of the whiskers and the dashed vertical line. Please add an axis description.

Figure 3: Please define ‘IMD’, ‘CI’, ‘TIA’
Figure 3: For consistency, we suggest changing the title to “Prepandemic and pandemic estimated Risk of Rapid Weight Gain (> 0.5 kg/m2/year) amongst adults living in England” or similar. Please indicate in the figure caption the meaning of the whiskers.

Figure 4: Please add a unit for ‘age group’. Please define ‘TIA’, ‘IMD’, ‘IQR’.
Figure 4: Please check the figure title and legend for grammar and spelling (e.g. ‘prepandemic\n’).**

Thank you. All figures have been updated to address these points. Please note that figure 3 and 4 have been revised using data from minimally adjusted (age and sex) adjusted models .

REFERENCES

**Request 52
PLOS uses the numbered citation (citation-sequence) method and first six authors, et al.
Please ensure that journal name abbreviations match those found in the National Center for Biotechnology Information (NCBI) databases (http://www.ncbi.nlm.nih.gov/nlmcatalog/journals), and are appropriately formatted and capitalised.

Please also see https://journals.plos.org/plosmedicine/s/submission-guidelines#loc-references for further details on reference formatting.

Where website addresses are cited, please specify the date of access.**

Thank you. All references have been updated in line with these guidelines and websites include the data cited.

SUPPLEMENTARY MATERIAL

**Request 53**

**Please define abbreviations used in the supplementary material (including figures and tables).
Please see requirements for Tables and Figures.

Appendix 2: Please define ‘BMI’.

Supplementary Figure 1: Please define ‘BMI’, ‘kg’, ‘m’.

Appendix 3: Please check whether you meant to reference Supplementary Figure 1 here. Please use a greater-than-or-equal sign.

Supplementary Figure 2: Please change the axis label from “Cummulative Frequency in Study Population” to “Cumulative Frequency in Study Population”.

Supplementary Table 1: Please change “Study populatio” to ““Study population” (2nd column). Please define ‘IMD’, ‘TIA’.

Supplementary Table 2: Please add a unit for the age groups. Please define ‘SD’, ‘IMD’, ‘TIA’, ‘kg’, ‘m’.

Supplementary Table 3: Please write ‘female’ and ‘male’ instead of ‘F’ and ‘M’. Please define ‘IMD’, ‘TIA’, ‘CI’, ‘kg’, ‘m’, ‘CI’. Please note that preceding some words (absent, present) you have added a colon, please remove.

Supplementary Table 4: Please write ‘female’ and ‘male’ instead of ‘F’ and ‘M’. Please define ‘IMD’, ‘TIA’, ‘CI’, ‘kg’, ‘m’. Please change ‘C’ (column 4&7) to ‘CI’ and define ‘CI’.

Supplementary Table 5: Please define ‘IMD’, ‘TIA’, ‘SMI’, ‘CI’, ‘kg’, ‘m’. Please note that, unlike other supplementary tables, you have written 'IMD' instead of 'Patient IMD' and 'SMI' instead of 'Serious Mental Illness'. Please ensure consistency in the use of abbreviations/full words.

Supplementary Table 6: Please write ‘female’ and ‘male’ instead of ‘F’ and ‘M’. Please define ‘TIA’, ‘SMI’, ‘CI’, ‘kg’, ‘m’. Please note that, unlike other supplementary tables, you have written 'SMI' instead of 'Serious Mental Illness'. Please ensure consistency in the use of abbreviations/full words.**

Thank you. All the supplementary figures have been adapted in line with these guidelines.

**Comments from the reviewers:**

**Reviewer #1: Kindly see the attachment for the comments.**
Thank you for your kind and helpful comments which have helped us to improve the manuscript. we have addressed them as follows.

1) The reviewer queried the term ‘living in deprivation’. In the introduction we have changed the text from ‘living in deprivation’ to living in low income neighbourhoods for clarity (line 138).
2) The reviewer queried if there were exclusion criteria. The study had specific inclusion criterion which is outlined in the methods (line 187 – 188), but no separate exclusion criteria.
3) The reviewer queried how BMI is opportunistically recorded. We have clarified the methods section to be clear how BMI is recorded and removed the term opportunistically as follows. “BMI is recorded in primary care records during routine health checks, disease monitoring pathways for conditions such as diabetes, and opportunistically when weight or BMI recording is indicated during the primary care consultation based on clinical judgement.” (lines 192 – 194)
4) The reviewer queried how BMI were randomly selected. We have explained in the Methods that BMI were selected randomly using a function in the R dplyr package. (lines 221 – 222)
5) The reviewer queried the meaning of “if patients were losing weight prepandemic, the rate of weight loss slowed.” We have clarified the following sentence as follows: ‘a positive δ-change indicated the rate of weight gain increased or, if patients were losing weight prepandemic, the rate of weight loss during the pandemic became slower than the prepandemic period (e.g. if an individual was losing 1kg/m^2^/year prepandemic which slowed to losing 0.5kg/m^2^/year during the pandemic). (lines 239 – 241)
6) We have explained the abbreviation IMD at its first use (line 250).
7) In the title ‘Rapid weight gain (> 0·5kg/m2 /year) in prepandemic and pandemic time periods’ the reviewer has suggested removing time or period as they both reference the same thing. We agree this would be clearer and have removed ‘time’ from the following sentence as suggested: Rapid weight gain (> 0.·5kg/m^2^/year) in prepandemic and pandemic periods. (line 337)

**Reviewer #2: Reviewer's Comments**
Thank you for your insightful and thorough review of this work. We have outlined how we have addressed the points you have raised below.

**Abstract:**

**Comment 1**
**What is IMD? Please expand.**

Thank you. We have clarified that IMD stands for Index if Multiple Deprivation in the abstract. (line 69) We have also explained what the meaning of IMD in the methods. “IMD is an area-based marker of deprivation based on an individual’s place of residence” (line 256) with a reference.

**Introduction:**

**Comment 2**
**Check if this sentence is correct: "Studies using routinely collected healthcare records have been limited to populations without universal access to healthcare…"**

Thank you. We have updated the sentence to state the following: “Previous studies using routinely collected healthcare records have been limited to populations accessing healthcare via specific health insurance providers in the United States of America [11,12], or individuals with long term conditions (LTCs) [13].” (lines 135 – 137). This differs to the situation in England, where through the NHS all individuals have access to healthcare, free at the point of delivery, irrespective of insurance or residency status and all individuals living in England are entitled to be registered with a General Practitioner free of charge.

**Comment 3**

**BMI recorded in Electronic Health Records (EHRs) do not provide a source of "nationally representative" data, because people who do not have access to healthcare and those who do not use healthcare are excluded. This, in fact, should be considered a limitation.**

Thank you. We have removed the term nationally representative from the introduction as, although registration with a GP is almost universal in the UK, data are limited to those who have accessed care. We have referenced a study in the introduction that demonstrated that prepandemic BMI values recorded in primary care data could provide population level BMI estimates that are comparable to nationally representative surveys. “Body Mass Index (BMI) data, a measure of weight adjusted for height (BMI = weight in kilograms (kg)/height in metres squared(m2)), recorded in English primary care Electronic Health Records (EHRs) have been shown to provide population level BMI estimates that are comparable to large nationally representative population surveys, and have been used to estimate rates of weight gain (BMI trajectories) before the pandemic [[15,16]](https://paperpile.com/c/Ux2xxc/FbEdo+vldXl)” (lines 144 – 148). However, we have discussed potential new biases introduced during the pandemic throughout the manuscript. Specifically the abstract: “However, changes in health care activity during the pandemic, e.g. a reduction in routine activity and increased remote consultations, may have introduced new and uncharacterised biases to the data” (lines 76 – 78); Non technical summary: “Changes in patterns of healthcare activity during the pandemic may have affected our results and we would welcome future research in this field, including population health surveys, incorporating sociodemographic, physical, and mental health characteristics, to explore these questions further” (lines 117 – 120) and; discussion: “However, we report changes in BMI recording activity that may have introduced new bias, with a decline in BMI recording after the onset of the pandemic which had only partly recovered by the year beginning March 2021, reflecting wider patterns of primary care activity during the pandemic[[26]](https://paperpile.com/c/Ux2xxc/JNPTV)” (lines 465 – 467).

**Comment 4**
**Please add the criteria (exact definition) for extreme-accelerators to this sentence: "…..identify individuals who had the greatest acceleration in their rate of weight gain during the pandemic (extreme-accelerators)."**

Thank you. We have added the precise criteria to the final paragraph of the introduction. “Using routinely collected English primary care records we conducted an observational cohort study to: (i) describe population-level changes in BMI recorded in healthcare records during the pandemic and (ii) estimate individual-level rates of weight gain before and during the pandemic to identify how (iia) risk of rapid weight gain (>0.5kg/m^2^/year) in the pre-pandemic and pandemic periods and (iib) risk of extreme acceleration in the rate of weight gain **(≥1.·84kg/m^2^/year)** between the prepandemic and pandemic period varied by sociodemographic and clinical characteristics” (lines 150 – 155).

**Methods:**

**Comment 5**
**Please clarify this. Isn't there a category or categories for non-binary persons? "We extracted data on all male and female adults."**

Thank you for this important query. Unfortunately non-binary status is not coded well in English primary care records and the number of individuals who are not assigned to male or female in the database is very low (<500 – personal communication). The study would therefore be underpowered to look at rates of weight change in non-binary persons.

**Comment 6
Provide the BMI formula instead of this: "a measure of weight adjusted for height"**

Thank you. We have clarified this as follows. “Body Mass Index (BMI) data, a measure of weight adjusted for height (BMI = weight in kilograms (kg)/height in metres squared(m2))” (lines 144 – 145).

**Comment 7**

**What do you mean by "BMI is recorded……….. opportunistically"?**

Thank you for asking for this clarification. We have expanded the text to be clearer about the indications for BMI recording in the healthcare records as follows. “BMI is recorded in primary care records during routine health checks, disease monitoring pathways for conditions such as diabetes, and opportunistically (i.e., when weight or BMI recording is indicated during the primary care consultation based on clinical judgement).” (lines 192 – 194)

**Comment 8**
**This is a major limitation, and substantially affects the accuracy of findings: "Recordings taken within healthcare settings and self-reported values are included in EHRs and cannot be differentiated using clinical codes."**

**It's a good decision by authors to characterize how the pandemic had influenced BMI data availability. I also assume that the proportion of self-reported values changed (likely increased) from pre-pandemic to pandemic, which may have significantly affected the findings.**

Thank you for this important query. This is a limitation which cannot, as data are currently coded, be unpicked from the data. We have expanded the discussion to be clearer about the potential impact of this limitation including the risk of uncharacterised bias.

“Pandemic-associated changes in clinical activity are likely to have introduced biases that are harder to characterise, such as an increase in the number of self-reported weight or height measures entered into clinical record during remote consultations[[30]](https://paperpile.com/c/Ux2xxc/cx61Y). This could cause systematic bias if some sociodemographic or clinical groups were more likely to have self-reported values recorded than others during the pandemic. As there is no clinical code to indicate whether weight and height measures are self-reported, the impact of this bias cannot be assessed using the available data. Further research, including representative population health surveys, would be required to investigate this further” (lines 478 – 484).

**Comment 9**
**Please provide a reference for this: "Extreme values (BMI<15kg/m2 and BMI>65kg/m2) were omitted from all quantitative analyses."**

Thank you. We have clarified that these cut offs were based on input from a clinical consensus panel of two primary care physicians and two diabetologists. “Extreme values (BMI<15kg/m^2^ and BMI>65kg/m^2^) were omitted from all quantitative analyses related to BMI values to censor erroneous results and exclude extremely overweight and underweight individuals whose patterns of weight change may not reflect those seen in the general population. Cut off values were decided by consensus of a clinical advisory panel (two diabetologists and two primary care clinicians)” (lines 201 – 205)

**Comment 10**
**Please justify your decision to calculate the population-level median and interquartile range rather than the mean and standard deviation. Provide at least one reference as well.**

Thank you. We have presented data as median and interquartile range as is best practice for skewed data to reduce the impact of extreme values on the average. We have clarified this in the methods with a reference. “Median (IQR) values are presented rather than means with standard deviation as recommended for data that are not normally distributed [[18]](https://paperpile.com/c/Ux2xxc/0vRc)” (lines 213 – 215).

**Comment 11**
**Provide a reference for this: "Individuals with known cancer and those underweight (BMI<18·5kg/m2) in the pre-pandemic period were excluded from these analyses as their patterns of weight change were likely to differ from the general population."**

We have clarified that these cut offs were decided with clinical input. “Through the recommendations of a clinical advisory panel, individuals with known cancer and those underweight (BMI <18.5kg/m^2^) in the prepandemic period were excluded from these analyses as rapid weight gain in these groups may have reflected improved disease control or other positive health outcomes” (lines 225 – 227).

**Comment 12**

**There are health conditions other than cancer and underweight (e.g., eating disorders) as well as treatments/interventions (e.g., bariatric surgery) that can affect weight change pattern. Why weren't they excluded?**

We have clarified that these exclusions were based on input from a clinical consensus panel of two primary care physicians and two diabetologists. We have also discussed in the methods due to the size of the data set we had to limit the analysis to common conditions with national quality frameworks. “Due to the size of the data set, extraction of LTC data was limited to conditions for which there were established primary care pathways associated with enhanced payments (Quality Outcomes Framework or Pay for Performance). These conditions were chosen for two reasons: firstly they represent conditions which have been identified as having a significant population health impact, and secondly, as data on the management of these conditions is submitted for enhanced payments the quality of clinical coded data is high” lines 256 – 261.

We do accept other conditions could have had an impact on patterns of weight gain and have referred to this as a limitation in the discussion. “Due to the size of the dataset we had to limit the analysis to common conditions which have established care pathways associated with national quality frameworks. We would welcome further research into how the pandemic influenced patterns of weight gain amongst adults living with other clinical conditions that could impact rates of weight gain, such as eating disorders” (lines 454-458).

**Comment 13**
**Please provide a reference for this as well: The most extreme 0·05% of values of δ-pre-pandemic and δ-pandemic (a positive or negative change of >6kg/m2/year) were censored to reduce the impact of erroneous results.**

Thank you. We have clarified in the methods that this cut off was data driven with input from a clinical advisory team. “The most extreme 0.05% of values of δ-prepandemic and δ-pandemic (a positive or negative change of >6kg/m^2^/year) were censored to reduce the impact of erroneous results. This cut off was defined by a data driven approach and confirmed with input from a clinical advisory panel” (lines 227 – 230).

**Comment 14
"Index of Multiple Deprivation" should appear in the first place where you mention about IMD, not in covariates section.
Please expand LTC.**

Thank you. We have corrected these points. Index of Multiple Deprivation is defined in the abstract and then in the covariates section where it is next discussed. We have explained Long Term Conditions (LTC) in the introduction (line 137).

**Comment 15**
**Statistical analysis is nicely written.
Age group are 18-39 years, 40-59 years, and 60-79 years. How did you include the covariate data from persons who transitioned from one age group to another between periods 1 and 2 or periods 2 and 3?**
We have clarified in the methods that as a retrospective analysis individuals were assigned to age groups based on their age during the study period. “As a retrospective analysis individuals were assigned to age groups based on their age at the end of the study period. Age in March 2020 was used in the analysis of prepandemic BMI trajectories. Age in March 2022 was used in the pandemic BMI trajectories and trajectory change analysis. Individuals were assigned to subgroups in the stratified analysis based on their age in March 2022.” (line 262 – 266).

**Results:**

**Comment 16
Please add the year beginning March 2020 to the Table-1.**

Thank you. We have added this information to table 1.

**Comment 17**
**In Figure-2 and Figure-3, what are the asterisks (1, 2, and 3 asterisks) referring to?
Please clarify the reference group for "Long Term Condition" in Figure-3.**

Thank you. We have updated all the figures in view of the advice from the academic editor to present the findings from minimally adjusted models. We have taken corrected and clarified the figures based on these comments.

**Comment 18
Tables and figures are very informative and easy to follow.**
**How was the absolute risk calculated for Figure-3 and Figure-4? Please explain in the statistical analysis section. In figures, you have presented the percentages as absolute risk. Absolute risk is the "probability" of an event, which is usually the number of events that occurred in a group, divided by the number of people in that group, so cannot be greater than one.**
Thank you. We agree that the use of the term risk is unclear when we are presenting prevalence estimates of the outcome and odds ratios. We have changed the figures and tables to remove reference to the term ‘risk’. We have instead referred to prevalence and adjusted odds ratios.

**Conclusions:**

**Comment 19**
**I recommend omitting the term "nationally representative" because the data are from EHR.**

We have omitted this term and referred instead to a national database of health data. “ Therefore routinely collected Electronic Healthcare Records (EHRs) have been shown to provide a national database of health data available during the pandemic[[16]](https://paperpile.com/c/Ux2xxc/pNiIH)” (line 427-429).

Wasantha Jayawardene


**Reviewer #3:**

Thank you for your thoughtful and considered review of this work. I have addressed the specific points you have raised below.

**The manuscript by Samuel et al investigates socio-demographic factors and BMI change before and during the pandemic. This is a very large study, comprising over 17m subjects, and the authors should be congratulated for carrying this out. The manuscript is well written and clear.**

We thank the reviewer for their positive comments.

**Comment 1
My major point is that the models aren't, as far as I can tell, adjusted for 'baseline' (i.e. pre-pandemic) BMI. My recommendation is that models are adjusted for baseline and updated, or a justification given as to why this hasn't been done.**

Thank you. We did not adjust for baseline BMI in the final analysis for the following reasons. Firstly as the main outcome (rate of BMI change) was calculated using the baseline BMI, there was a risk that this could introduce complex bias into the analysis. Secondly, as baseline BMI is likely to be distal in the causal pathways to exposures of interest (such as deprivation and mental health concerns) and the studied outcomes, it may in fact mediate (rather than confound) these associations. In the revised manuscript we have taken on board the academic editor’s comments that fully adjusted models ‘might diminish the extend of disparities that are observed from a descriptive perspective’ and have therefore only presented minimally adjusted models, adjusted only for age and sex as such we do not believe it would be appropriate to adjust for baseline BMI at this time.

**Minor points:**

**Comment 3**
 **Why does the grouping of age change between the main analyses and the subgroup analysis?**

Thank you. We have clarified that we collapsed groups in the subgroup analysis to reduce risk of disclosure from rare events. “Age groups were also collapsed into larger groups (e.g. 18-29 and 30-39 years collapsed into 18-39 years) to reduce the risk of disclosure from rare events” (lines 298-300).

**Comment 4**
**Be clear which years correspond to which period. This is illustrated in the supplement but could be clearer in the main text.**
Thank you. We agree that this may have been unclear in points and have clarified the methods section using the text used in the supplementary material.

“We first estimated individual level BMI trajectories as rates of BMI change per year (δ) in kg/m2/year, before the onset of COVID-19 pandemic (δ-prepandemic) and after the onset of the pandemic (δ-pandemic). BMIs were classified into time periods: period-1: March 2015 - February 2018; period-2: March 2018 - February 2020; and period-3 March 2020 - February 2022. For patients with more than one BMI measure from each period, a random BMI measure was selected from each period using the slice_sample function from the R dplyr package. Where data were available, BMI data from period 1 and period 2 were used to calculate δ-prepandemic, while BMI data from period 2 and period 3 were used to calculate δ-pandemic. Rate of BMI change/year was calculated between these time points assuming a linear trend[[15]](https://paperpile.com/c/Ux2xxc/FbEdo)(S1 Appendix, S1 Figure)” (lines 217 – 224).
