# Supplementary material for: Trends in weight gain recorded in English primary care before and during the Coronavirus-19 pandemic: An observational cohort study using the OpenSAFELY platform
Source: PLoS Med. 2024 Jun 24;21(6):e1004398. doi: 10.1371/journal.pmed.1004398 (PMC11249215; doi:10.1371/journal.pmed.1004398)
Supplement: Attachment — Submitted filename: PLOS_revision_2_rebuttal.docx [file pmed.1004398.s018.docx]

Dear PLOS Medicine Editorial team,

Thank you for giving us the opportunity to further revise and improve the manuscript. We are pleased that our revisions satisfied the original reviewers. We appreciate the need for robust statistical review and appreciate Reviewer #4’s comments and suggestions for improvement. We have responded to each of these as outlined below.

We note that Reviewer #4 has discussed the risks of presenting minimally adjusted models in terms of over inflating the contribution of specific conditions such as Serious Mental Illness and we have addressed this concern in the response below. As we discussed in our email correspondence with Dr Schaefer, whereas we agree that individuals with Serious Mental Illness are at risk of multimorbidity and the associations between Serious Mental Illness and patterns of weight gain may be mediated through other conditions, we believe this strays into the territory of causality which, in line with the recommendations of the first academic editor, we have steered away from in this analysis. Our findings are primarily designed to describe which groups were at risk of unhealthy patterns of weight gain to guide clinicians and policy makers. Notably, we believe a complex analysis of how multimorbidity interacts and mediates associations between individual health conditions and patterns of weight gain was beyond the scope of this analysis. Due to the scale of this study, we only extracted data on common conditions outlined in the text. We are concerned that limiting the classification of multimorbidity to these conditions would under-represent the burden of multimorbidity in female and minority populations due to the exclusion of conditions such as anaemia and dysmenorrhoea (Eto, Samuel et al 2023, PLOSMedicine, https://doi.org/10.1101/2023.03.03.23286751). However, we would hope our analysis would stimulate further research which addresses these focussed causal questions. We have, as discussed in email correspondence with the Executive Editor, expanded our discussion to highlight these considerations and limitations and presented the models adjusted for age, sex, ethnicity and IMD as supplementary material.

We are truly appreciative of your continued input and believe our work has been further improved by the further suggestions made.

Thank you for your continued consideration.

Your Sincerely,

Dr Miriam Samuel

**Point 1.**

*“What is TPP?”*

Thank you for this query. ‘OpenSAFELY inside TPP’ is the naming convention for the platform used to access the data and naming it as such is a precondition of publishing research using this platform. TPP is a GP electronic healthcare software which includes the routine primary health records of 40% of the population living in England. Due to the strict word limits we have not been able to expand on this in the abstract. However, we have made this clearer in the Methods.

“Primary care health records, managed by the GP software provider TPP, were linked, stored, and analysed securely within the OpenSAFELY platform inside TPP, containing pseudonymised data on approximately 40% of the English population, including coded diagnoses, medications and physiological parameters. No free text data are included. Further details are available at <https://opensafely.org/>.” (line 154 -158)

**Point 2**
*“Page 2 Strictly speaking multivariable is meant here (Hidalgo and Good-man, 2013). Multivariate means multiple variables on the left hand side. I do not think that is what the authors did.”*

Thank you for this point of clarification. We have changed this. (line 64)

**Point 3**

*“For readers not familiar with the platform the data source section is rather scant. Where did all this primary care data come from? Is it from one of the existing primary care databases? What validation has been performed on it?”*

Thank you for this query. We have expanded the methods section to make this clearer and changed the structure of the paragraph to indicate further details on the data platform can be found on the opensafely.org website.

“Primary care health records, managed by the GP software provider TPP, were linked, stored, and analysed securely within the OpenSAFELY platform inside TPP, containing pseudonymised data on approximately 40% of the English population, including coded diagnoses, medications, and physiological parameters. No free text data are included. Further details are available at <https://opensafely.org/>.” (line 154 -158)

**Point 4**

*“I have only looked at a few of the files on GitHub and, obviously, not run them but they do appear appropriately commented.”*

We thank the reviewer for their positive comments

**Point 5**

*“How many were excluded as BMI outliers? This could go in Figure 1. Apologies if I have missed it somewhere else but it does belong in the flowchart.”*

Thank you for this request for clarification. We have added these details to figure 1.

**Point 6**

*“A citation to the package used (dplyr) would be appropriate. It is the only credit authors get (disclaimer: I am a package author but not of any packages involved here).”*

Thank you. We have included this citation.

**Point 7**

*“Why was it felt necessary to categorise age? Categorising an essentially continuous variable wastes information (Altman and Royston, 2006; Royston et al., 2006) and leads to models which are often implausible as they predict the effect remaining at within categories and then jumping to a new value at the category boundary. From the plots a simple function of age would have been sufficient.”*

Thank you. As we structured the study as a descriptive analysis, we categorised age based on the input from our clinical group that age categories would make the results easier to interpret and translate into recommendations for clinical practice. We do accept the flattening of effect across the age category is a limitation and have added the following line to the discussion.

“We categorised age for ease of interpretation and application to clinical practice. But this may have flattened effects within categories and over emphasised the step change in effect between categories.” (line 465-467)

**Point 7**

*“What does chronic cardiac conditions cover?”*

Thank you for this request for clarification. We used the term to describe diseases that affect the heart, but we have now updated the text to make that explicit.

“cardiovascular disease (CVD: conditions affecting the heart)” (line 253 – 254)

We have also added a link at the end of this paragraph to the codelists used to define each of the disease phenotypes. These contain detailed descriptions of all the clinical codes used to identify conditions in the routine healthcare records.

“The complete code lists used to define each LTC (hypertension, T1D, T2D, asthma, COPD, SMI, learning difficulties, dementia, CVD, stroke and TIA) in the health record are available for open access and review (https://github.com/opensafely/BMI-and-Metabolic-Markers).” (line 264 - 267)

**Point 8**

*“I do not understand why the authors used two different ways of assigning age. My experience is that researchers usually use age at cohort entry but age at study exit can be justified if it makes the interpretation more sensible.”*

Thank you for this request for clarification. We have tried to clarify that the prepandemic weight change analysis is effectively a separate analysis in a different cohort that has been included to give context to the main analysis which has been conducted in the pandemic cohort.

“Individuals were assigned to age groups based on their age at the end of the relevant study period. Age in March 2022 was used in the main analyses (pandemic BMI trajectories and trajectory change) and to assign individuals to subgroups in the stratified analysis. Age in March 2020 (the end of the prepandemic period) was used to assign age in the analysis of prepandemic BMI trajectories, which was conducted to contextualise the main findings.” (line 255 - 258)

**Point 9**

*“Where does the ethnic coding come from? Do general practitioners actually use the census categories? There is no mention of the database being linked to ONS census data. The White population of England seems to have been 85% in 2011 and 81% in 2021 both somewhat higher than that given here. Why is that?”*

Thank you for this query. Ethnicity is recorded in health data based on census categories. We have added a reference to the detailed information on how data are recorded in routine health data and categorised in the OpenSAFELY platform. Notably we have broken up the higher level ‘White’ category into “White British, White Irish and White Other’. This would explain why the proportion of White British adults (76.1%) is less than the ‘White’ population data from the census.

“ethnicity recorded in primary care records and categorized according to the 2001 UK Census definitions (White British, White Irish, Other White, Black African, Black Caribbean, Other Black, Indian, Pakistani, Bangladeshi, Chinese, Other Asian, Mixed White/Black African, Mixed White/Black Caribbean, Mixed White/Asian, Other)[18];” (line 245 - 248)

**Point 10**

*“Page 10 Why present decide to present a comparison of 18-29 year olds specifically versus 50-59?”*

Thank for this query. We have presented these age groups as an example for comparison. We have expanded the text to clarify this is an example.

“The prevalence and adjusted odds of rapid weight gain reduced with age, for example prevalence was 30.08% prevalence in 50-59-year-olds compared to 44.18% in 18-29-year-olds (aOR 0.60 [0.59, 0.60], p<0.001” (line 346 - 347)

**Point 11**

*“Page 11 In the caption to Figure 4 it is unnecessary to spell out kg or m.”*

Thank you for this advice. We have amended the caption. (line 387 – 388)

**Point 12**

*“Page 11 I could not find the sensitivity analysis explained in the methods and I can only understand this if there were in fact two analyses as I cannot otherwise grasp how the results for Black people could be modified by including people with missing ethnicity.”*

Thank you for asking for this clarification. It previously read as though the whole paragraph was referring to the sensitivity analysis. We have clarified that this paragraph discusses both the sensitivity and subgroup analyses, and signposted which sentence refers to which.

“In the sensitivity analysis, we compared our findings in the complete case sample to analyses conducted in the entire population, including those with missing ethnicity and deprivation data, and found our results to be consistent in both groups. In the subgroup analyses, we found that most of the estimated associations between the risk of extreme acceleration in rate of weight gain and age, sex, IMD and LTCs persisted in the analyses stratified by age, sex, ethnicity, and deprivation quintile. But there were some exceptions, e.g. amongst Black individuals, deprivation was not associated with odds (IMD5 vs IMD1: aOR 1.04[0.92,1.18], p = 0.496); and amongst those living in the least deprived quintiles, Black individuals were at higher risk than Whites (Black vs White: aOR 1.24 [1.10,1.39], P<0.001).” (line 399 - 406)

**Point 13**

*“Page 12 The authors claim that previous studies have used non-representative samples. For various reasons outlined by the authors themselves on page 13 the fact that people had to have their weight recorded must undermine any very strong claim to representativeness. Their sample may well approximate a random sample of adults living in England more closely than other studies. I am not blaming the authors here, when using routine data we can only analyse what other people have recorded not what we would have liked them to record.”*

Thank you for this comment. We are glad the reviewer has highlighted our extensive discussion on the limitations of using routine health data. We have aimed to be transparent about new bias that could have occurred due to changes in BMI recording during the pandemic. In response to this comment we have restructured the sentence on previous studies and removed the word ‘non-representative’ to prevent readers from inferring that our study is representative.

“Previous research in this area has been limited to small voluntary surveys [10], or routine health data from populations with specific comorbidities or in geographical regions where access to healthcare is stratified by structures of payment and insurance[11–14].” (line 427 - 429)

**Point 14**

*“Figure 1 It would be helpful to add the numbers still included in all the boxes down the main path to provide a basis for the importance of the exclusions.”*

Thank you for this useful suggestion. We have included more data in Figure 1 in line with this.

**Point 15**

*“Table S6. The column widths need adjusting here. Splitting the confidence interval over two table rows may be unavoidable but splitting in the middle of a number is super- ugly. Tables S7 to S9 do not share this problem.”*

Thank you for pointing this out. We believe this was an error in the upload of the table. We had not originally formatted it in this way and will review the submission carefully to check this does not happen again.

**Point 16**

*“I found the use of δ for change confusing as Δ is almost universal in mathematics for change.”*

Thank you for this comment. We would prefer to continue to use δ as this aligns with terminology we have used in an aligned analysis but can change this if required and will be mindful of this for future work.

**Point 17**

*“It might be helpful to on page 5 to clarify what original models the minimally adjusted models replaced.”*

Thank you for this comment. We have updated the text to be clear which models we presented in the initial submission and how these were adjusted to be in line with the suggestions of the academic editor.

“Following the first round of peer review, we have presented the results of minimally adjusted statistical models (adjusted for age and sex) in line with the descriptive focus of the analysis. This replaced models adjusted for age, sex, ethnicity and Index of Multiple Deprivation (IMD) quintile, which we have included in the supplementary material. This study is reported as per the Reporting of studies Conducted using Observational Routinely-collected health Data (RECORD) Statement (S1 Checklist).” (line 171 - 174)

**Point 18 and Point 19**

*“What model underlies the results on, for example, page 10. Is each just adjusted for age and sex or for all the others in the table? The captions seem to suggests that each is only adjusted for age and sex but not for the others. That seems a strange decision to me. If we just take the long term conditions it is now generally accepted to people with SMI have poor physical health and reduced life expectancy. So any coefficient for SMI also represents the effect of other long term conditions.”*

*“2.1 Adjustment of the covariates. I clearly disagree with some of the reviewers' comments over minor matters but the major point of issue is the decision to move from a fully adjusted model to one just using age and sex as covariates. If you have a number of potential covariates which are associated then presenting each unadjusted for the others means that you are potentially reporting an inated number of associations. There is also a danger that if you collapse over important covariates you run the risk of Simpson's paradox (Simpson, 1951). The issue seems particularly important with the long term conditions. As I mentioned in my original comments reporting the coefficient for SMI un-adjusted is also mixing in associations with other illnesses. The same is probably true for other conditions. I imagine similar concerns could be raised about area deprivation and ethnicity.”*

Thank you for these comments. We have tried to be clear throughout that this is a descriptive study. We discussed as a team the suggestion of the original academic editor that minimally adjusted models would clarify that this was the approach and steer the focus of the analysis away from ‘causal’ territory.

We agree with Reviewer #4’s comments that the associations between SMI and unhealthy patterns of weight gain are likely mediated through pathways involving deprivation and comorbidity. We have therefore added the following paragraph to the discussion to highlight this and to emphasise that we are not arguing that the observed associations are not causal.

“As a descriptive study we did not seek to assign a causal relationship between the exposures and the outcomes, nor identify the pathways that mediate observed associations. In line with this approach we have presented minimally adjusted models that have only been adjusted for age and sex. Therefore the observed relationship between exposures, such as mental health conditions, and unhealthy patterns of weight gain may have been mediated through pathways including comorbid physical health conditions and increased deprivation. Our findings emphasise the need for further research to unpick the complex relationships between physical health, mental health, sociodemographic characteristics, and patterns of weight gain.” (line 502 - 508)

Due to the scale of the analysis we only extracted data on a range of common conditions that were common and likely have high quality data due to their linkage with NHS payment frameworks. We did not design this study to assess the contribution of multimorbidity to the patterns of weight change described and would be very concerned that limiting our definition of multimorbidity to these conditions would under-represent the burden of multimorbidity experienced by women and minority populations. Due to the scale of this analysis we did not extract data on clinical phenotypes such as ‘painful conditions’ and ‘anaemia’ which are more prominent in clusters of multimorbidity stratified by sex and ethnicity [1]. Our aim was to conduct a large-scale study that describes health inequalities in patterns of weight gain experienced by a range of clinical and sociodemographic subgroups living in England during the COVID-19 pandemic. We hope this would stimulate conversations and further research on the causal pathways that underlie these observed associations. We do believe that describing these patterns is an important first stage in informing more focussed work and the need to include sociodemographic, physical and mental health conditions in research, policy and interventions in this field. We have highlighted the need for further research in our closing paragraph.

“As a descriptive analysis, our findings identified clear targets for health services and policy makers to target with preventative interventions. We would welcome further focussed research to understand the complex causal pathways that underlie the associations we describe in this analysis.” (line 514 - 517)

References

1. Eto F, Samuel M, Henkin R, Mahesh M, Ahmad T, et al. (2023) Ethnic differences in early onset multimorbidity and associations with health service use, long-term prescribing, years of life lost, and mortality: A cross-sectional study using clustering in the UK Clinical Practice Research Datalink. PLOS Medicine 20(10): e1004300. <https://doi.org/10.1371/journal.pmed.1004300>
