# Supplementary material for: Trends in weight gain recorded in English primary care before and during the Coronavirus-19 pandemic: An observational cohort study using the OpenSAFELY platform
Source: PLoS Med. 2024 Jun 24;21(6):e1004398. doi: 10.1371/journal.pmed.1004398 (PMC11249215; doi:10.1371/journal.pmed.1004398)
Supplement: Attachment — Submitted filename: PLOS_revision_3_rebuttal.docx [file pmed.1004398.s019.docx]

Dear PLOS Editorial team,

Thank you for the opportunity to further revise this work. We understand that statistical models can have different conceptual arguments and the importance of addressing Reviewer 4’s concerns. We have therefore presented the models adjusted for age, sex, ethnicity, and deprivation as suggested by Dr Schaefer. We have also addressed the specific points and suggestions made by the editorial team and indicated where these changes have been made in the response below.

Please note that changing the models from age and sex adjusted models to age, sex, ethnicity, and deprivation adjusted models has resulted in small changes to several adjusted Odds Ratios and p-values in the text which are not individually described below.

As we have previously discussed, the emphasis of this analysis is to describe sociodemographic and clinical subgroups at increased risk of unhealthy patterns of weight gain. We believe this large-scale study presents strong evidence of inequalities in pattens of weight gain that can guide policy makers on how weight management resources can be prioritised. We also hope that our findings will prompt further research unpicking the complex relationship between sociodemographic characteristics, physical and mental health, and patterns of weight gain.

We strongly believe this iterative process of revision has improved the work overall and thank the reviewers and editorial team for their continued input.

Yours Sincerely

Dr Miriam Samuel

**Requests from Editors:**

**ABSTRACT**
*Request 1*
*1) ll.66-70: Please change to: “Rapid pandemic weight gain was associated with sex, age, and Index of Multiple Deprivation (IMD). Male sex (male vs. female: adjusted Odds Ratio (aOR) 0.76 [95% Confidence Interval: 0.75, 0.76], p<0.001), older age (e.g. 50-59 years vs. 18-29 years: aOR 0. 60 [0.59, 0.60], p<0.001]) and living in less deprived areas (least deprived IMD quintile vs most deprived: aOR 0.80 [0.80, 0.81], p<0.001) reduced the odds of rapid weight gain.”*

Thank you. We have changed the text to align with these comments. (lines 34-37)

“Rapid pandemic weight gain was associated with sex, age, and IMD. Male sex (male vs female: adjusted Odds Ratio (aOR) 0.76 [95% Confidence Interval: 0.76, 0.76], p<0.001), older age (e.g. 50-59-years vs 18–29-years: aOR 0.60 [0.60, 0.61], p<0.001]); and living in less deprived areas (least-deprived-IMD-quintile vs most-deprived: aOR 0.77 [0.77, 0.78] p<0.001)) reduced the odds of rapid weight gain.”

*Request 2*
*2) ll.66-70: We removed "(n=3,214,155)" because it might mislead readers into thinking that this is the number of people with rapid weight gain, when in fact it is the number of people with available data for the calculation.*

Thank you. We have removed this number and also ‘n=2,768,695’ which referred to the number of individuals included in the acceleration in rate of weight gain analysis, for analogous reasons. (line 40-42)

*Request 3*
*3) ll.70-72: “Adults of White British ethnicity had higher odds of rapid weight gain than all other ethnicities (e.g. White British vs Indian: aOR X.XX  [X.XX, X.XX], p<X.XXX)” - Since you are presenting the results from a White British perspective, we request that the relevant data be presented the other way around, rather than from a perspective of lower odds, as is currently done. Also, please clarify that these values refer to pandemic weight gain, not prepandemic.*

Thank you. We understand the reason for the request, however as there are more than two ethnic groups in this regression model, we have received advice from a Professor of statistics that presenting the aOR from a White British perspective would not be as simple as 1/0.69 etc. Rerunning the analysis in the OpenSAFELY platform and waiting for the outputs to be approved for release would cause significant delays. We have therefore reworded the following sentence in the abstract to change the perspective and hope this aligns with the spirit of the comment.

“Compared to White British individuals, all other ethnicities had lower odds of rapid pandemic weight gain (e.g. Indian vs White British: aOR 0.69 [0.68, 0.70], p<0.001).” (line 37-39).

**AUTHOR SUMMARY**
*Requests 4 and 5
1) l.85: Please change to “Author Summary”.*
 *2) l.99: Please change to “White British” and revise throughout the main manuscript.*

Thank you. We have adjusted the text as requested.

*Request 6
3) Please rephrase the first bullet point under "What do these findings mean?" as it currently seems to repeat the results. Editorial suggestion: The COVID-19 pandemic appears to have had the greatest impact on women, young adults, and those living in the most deprived areas in terms of unhealthy patterns of weight gain.*

Thank you. We have adjusted the text in line with the editors’ suggestion. (line 72-73)

“The COVID-19 pandemic appears to have had the greatest impact on women, young adults, and those living in the most deprived areas in terms of unhealthy patterns of weight gain.”

*Request 7
4) The last bullet point should describe the main limitation(s) of the study's methodology only. Please revise.*

Thank you. We have updated the final bullet point as below. (line 80-81)

“Changes in healthcare activity during the pandemic affected patterns of weight monitoring in primary care, which may have introduced new bias to the analyses.”

**METHODS AND RESULTS**
*Request 8*

*1) We feel that there are currently too many subheadings that divide the text into unnecessarily many sections. Please revise. For example, “Population level trends in BMI recording activity and median BMI”, “Individual level BMI trajectories” and “Defining a population of extreme accelerators” should form one section under “BMI trajectories” or similar.*

Thank you. We have collapsed all these subheadings under ‘Study Outcomes: Body Mass Index (BMI) trends (line 148).

*Request 9*
*2) Similarly, please remove the subheadings under “Individual level BMI trajectory analyses” as we feel the heading is sufficient to guide the reader through the results.*

Thank you. We have removed these subheadings.

*Request 10*
*3) ll.171-174: Please revert the text describing the change in analysis to focus on the minimally adjusted model, as a result of the peer review process. Modification will also be required at ll.287-288.*

Thank you. We have removed reference to changes made as a result of peer review (lines 127-137). We have also modified the text under ‘Statistical Models’ as follows. (line 249)

“We present the results of multivariable models adjusted for age, sex, IMD and ethnicity.”

*Request 11
4) ll.321-322: Is it worth noting that differences between prepandemic and pandemic, e.g. in the 70-79 age group, were generally quite modest.*

Thank you. We have added the following sentence to highlight some increases were modest. (lines 283-285)

“Although for some groups, such as individuals living in the least deprived IMD quintile, this increase was quite modest (IMD 5: 27.1kg/m^2^ [23.9, 30.9] in 2019 to 27.1kg/m^2^ [23.9, 31.2] in 2021). “

*Request 12*
*5) l.346: Please specify in the first sentence that the results presented are for the pandemic period.*

Thank you. We have adjusted the sentence (line 308).

“ The prevalence and adjusted odds of rapid pandemic weight gain reduced with age”

*Request 13*
*6) l.380: Please define “Adults” and remove the duplicate “with”.*

Thank you. We have adjusted the sentence as follows (lines 343-344).

“Individuals with T1D had a lower prevalence and adjusted odds (9.38%, aOR 0.87[0.85, 0.90] p<0.001) of extreme acceleration than those without T1D. “

*Request 14*
*7) ll.403.404: Please change to “But there were some exceptions, e.g. among Black individuals, deprivation was not associated with weight gain (IMD5 vs IMD1: aOR 1.04[0.92,1.18], p = 0.496);…”*

Thank you. We have changed the sentence as follows (lines 366 – 368).

“But there were some exceptions, e.g. amongst Black individuals, deprivation was not associated with extreme acceleration in rate of weight gain (IMD5 vs IMD1: aOR 1.04[0.92,1.18], p = 0.496)”

*Request 15.
8) l.410: Please add “years” to “aged 60-79”.*

Thank you. We have added 60-79 years (line 374).

**DISCUSSION**

*Request 16*
*1) l.417: Please add “to our knowledge” or similar, i.e. “We report, to our knowledge, novel findings...”.*

Thank you. We have added this to the sentence (line 380).

“We report, to our knowledge, novel findings from our analyses of BMI data in the EHRs of over 17 million adults living in England.”

*Request 17*
*2) ll.478-480: “Alternatively this may be a true effect arising from the awareness that COVID-19 infection confers greater risk to people with obesity and LTCs such as T2D,…” – please provide reference.*

Thank you. We have added a reference (line 445).

*Request 18.*
*3) We suggest that the discussion should mention the problems (e.g. health problems) associated with weight gain/rapid weight gain and discuss initiatives that have been taken to address this issue (e.g., the introduction of the Soft Drink Industry Levy) in order to delve deeper into the implications and next steps for research, clinical practice, and/or public policy.*

Thank you. We have added the following to the closing paragraph (lines 478 – 485).

“Rapid weight gain is an independent risk factor for cardiovascular disease and mortality [1], and associated with an increased risk of progressions to obesity[14]. Overweight and obesity is one of the greatest global public health challenges of our time and projected to cost the global economy 3.3% of gross domestic product by 2060. In response, the World Health Organisation acceleration plan to stop obesity emphasises the need for country level interventions such as fiscal measures to reduce consumption of ultra-processed food and beverages, and health services for the preventions and management of obesity[35]. Our findings identified clear targets for such preventative interventions.”

**REFERENCES**

*Request 19*
1) Where website addresses are cited, please add the word “Accessed” when specifying the date of access (e.g. [accessed: 12th February 2024]).

Thank you. We have added this to the references.

**FIGURES**

*Request 20*
*1) Figure 1: In the first box, please change to “aged 18 to ≤90 years”.*

Thank you. We have updated this.

*Request 21 - 22*
*2) S1 Figure: Please be sure to define the difference between "aOR" and "aOR**" in the figure description. Assuming that the aOR** values represent the fully adjusted prepandemic values, we wonder why the minimally adjusted prepandemic aOR values are not shown, but the pandemic aOR values are? Also, the aOR** values for the prepandemic period appear to be identical to the aOR values listed under "Pandemic" - is this correct? Please revise.

3) Figure S2: The figure descriptions for Figure S2 and Figure 4 appear to be identical. Does Figure S2 show the fully adjusted values? Please revise and be sure to display two decimals in Figure S2 ("extreme acceleration") for consistency.*

Thank you. There was an error in S1 Figure which was uploaded which we apologise for not spotting. However, S1 and S2 Figures have been removed from the manuscript as they presented the ‘fully adjusted’ models when age and sex adjusted models were presented in the main text. As we are now presenting the ‘fully adjusted’ models in the main text, these figures are no longer needed.

**SOCIAL MEDIA**

*To help us extend the reach of your research, please provide any X (formerly known as Twitter) handle(s) that would be appropriate to tag, including your own, your co-authors’, your institution, funder, or lab. Please enter in the submission form any handles you wish to be included when we post about this paper.*

Thank you. We will create a list of authors’ social media handles and forward these to the editorial team.

**Comments from Reviewers:**

*Request 23.
Reviewer #4: My main point was the use of a model which did not adjust for other covariates. I seem to be in a small minority here but I am not convinced by the rebuttal. Let us look at examples to make concrete my concern.

The well known Berkeley admissions data* [*https://eur01.safelinks.protection.outlook.com/?url=https%3A%2F%2Fen.wikipedia.org%2Fwiki%2FSimpson%2527s_paradox%23UC_Berkeley_gender_bias&data=05%7C02%7Cm.samuel%40qmul.ac.uk%7C978f0f2ba8734b1baa0208dc43573827%7C569df091b01340e386eebd9cb9e25814%7C0%7C0%7C638459289085650829%7CUnknown%7CTWFpbGZsb3d8eyJWIjoiMC4wLjAwMDAiLCJQIjoiV2luMzIiLCJBTiI6Ik1haWwiLCJXVCI6Mn0%3D%7C0%7C%7C%7C&sdata=4zIPOZanTEuary76IywRT5ZxA6NO7sUBwVjIoJSC2AU%3D&reserved=0*](https://eur01.safelinks.protection.outlook.com/?url=https%3A%2F%2Fen.wikipedia.org%2Fwiki%2FSimpson%2527s_paradox%23UC_Berkeley_gender_bias&data=05%7C02%7Cm.samuel%40qmul.ac.uk%7C978f0f2ba8734b1baa0208dc43573827%7C569df091b01340e386eebd9cb9e25814%7C0%7C0%7C638459289085650829%7CUnknown%7CTWFpbGZsb3d8eyJWIjoiMC4wLjAwMDAiLCJQIjoiV2luMzIiLCJBTiI6Ik1haWwiLCJXVCI6Mn0%3D%7C0%7C%7C%7C&sdata=4zIPOZanTEuary76IywRT5ZxA6NO7sUBwVjIoJSC2AU%3D&reserved=0) *showed that women were less likely to be admitted. This was not evidence of bias since a more detailed analysis taking area of study into account, if anything, showed the reverse.

If the authors prefer examples using continuous variables we can note that men have larger brains than women (1345g v 1222 according to Wikipedia) a fact which men saw as justifying the continued subjugation of women. If we adjust for body weight (UK 85.4Kg v 72.1Kg, same source) we find the reverse.

One important thing here is that I do not think people saw these as anything other than descriptions so I find the authors insistence that they are only doing a descriptive analysis rather puzzling. Descriptions can mislead.

Michael Dewey


Any attachments provided with reviews can be seen via the following link:*
------------------------------------------------------------

Thank you for your continued input. In alignment with discussion with the editorial team the main manuscript has reverted to age, sex, ethnicity and IMD adjusted models.

**General Editorial Requests**

*Request 24
1) We ask every co-author listed on the manuscript to fill in a contributing author statement. If any of the co-authors have not filled in the statement, we will remind them to do so when the paper is revised. If all statements are not completed in a timely fashion this could hold up the re-review process. Should there be a problem getting one of your co-authors to fill in a statement we will be in contact. YOU MUST NOT ADD OR REMOVE AUTHORS UNLESS YOU HAVE ALERTED THE EDITOR HANDLING THE MANUSCRIPT TO THE CHANGE AND THEY SPECIFICALLY HAVE AGREED TO IT.*

Thank you. We have chased all authors to complete the necessary documentation.

*Request 25*
*2) Please ensure that the paper adheres to the PLOS Data Availability Policy (see* [*https://eur01.safelinks.protection.outlook.com/?url=http%3A%2F%2Fjournals.plos.org%2Fplosmedicine%2Fs%2Fdata-availability&data=05%7C02%7Cm.samuel%40qmul.ac.uk%7C978f0f2ba8734b1baa0208dc43573827%7C569df091b01340e386eebd9cb9e25814%7C0%7C0%7C638459289085659465%7CUnknown%7CTWFpbGZsb3d8eyJWIjoiMC4wLjAwMDAiLCJQIjoiV2luMzIiLCJBTiI6Ik1haWwiLCJXVCI6Mn0%3D%7C0%7C%7C%7C&sdata=52QuX3ip0%2B%2B9OQVTglyLcCbfIP1rM%2Fq0UIk1yn9f47E%3D&reserved=0)*](https://eur01.safelinks.protection.outlook.com/?url=http%3A%2F%2Fjournals.plos.org%2Fplosmedicine%2Fs%2Fdata-availability&data=05%7C02%7Cm.samuel%40qmul.ac.uk%7C978f0f2ba8734b1baa0208dc43573827%7C569df091b01340e386eebd9cb9e25814%7C0%7C0%7C638459289085659465%7CUnknown%7CTWFpbGZsb3d8eyJWIjoiMC4wLjAwMDAiLCJQIjoiV2luMzIiLCJBTiI6Ik1haWwiLCJXVCI6Mn0%3D%7C0%7C%7C%7C&sdata=52QuX3ip0%2B%2B9OQVTglyLcCbfIP1rM%2Fq0UIk1yn9f47E%3D&reserved=0))*, which requires that all data underlying the study's findings be provided in a repository or as Supporting Information. For data residing with a third party, authors are required to provide instructions with contact information for obtaining the data. PLOS journals do not allow statements supported by "data not shown" or "unpublished results." For such statements, authors must provide supporting data or cite public sources that include it.*

Thank you. Information on the data source is included in the main text as below (lines 118 – 125) and further details are included in S1 Appendix.

“Primary care health records, managed by the health record software provider TPP (<https://tpp-uk.com/>), were linked, stored, and analysed securely within the OpenSAFELY platform inside TPP, which contains pseudonymised data on approximately 40% of the English population, including coded diagnoses, medications and physiological parameters. No free text data are included. Further details are available at <https://opensafely.org/>. Detailed pseudonymised patient data is potentially re-identifiable and therefore not shared. The study was approved by the London School of Hygiene & Tropical Medicine (LSHTM) Ethics Board (reference 26536). An information governance statement is included (S1 Appendix).”

*Request 26
3) Please review your reference list to ensure that it is complete and correct. If you have cited papers that have been retracted, please include the rationale for doing so in the manuscript text, or remove these references and replace them with relevant current references. Any changes to the reference list should be mentioned in the rebuttal letter that accompanies your revised manuscript.*

Thank you. We have reviewed the reference list which is still current.

__________________________________________________
In compliance with data protection regulations, you may request that we remove your personal registration details at any time.  (Use the following URL: <https://eur01.safelinks.protection.outlook.com/?url=https%3A%2F%2Fwww.editorialmanager.com%2Fpmedicine%2Flogin.asp%3Fa%3Dr&data=05%7C02%7Cm.samuel%40qmul.ac.uk%7C978f0f2ba8734b1baa0208dc43573827%7C569df091b01340e386eebd9cb9e25814%7C0%7C0%7C638459289085669167%7CUnknown%7CTWFpbGZsb3d8eyJWIjoiMC4wLjAwMDAiLCJQIjoiV2luMzIiLCJBTiI6Ik1haWwiLCJXVCI6Mn0%3D%7C0%7C%7C%7C&sdata=oWhm7%2BFPSot0ZU6p0g4B3%2BOSzKD6mBMq3RadEgE31%2FE%3D&reserved=0)>. Please contact the publication office if you have any questions.
